# Screening methods for detection of ancient *Mycobacterium tuberculosis* complex fingerprints in next-generation sequencing data derived from skeletal samples

**DOI:** 10.1093/gigascience/giz065

**Published:** 2019-06-20

**Authors:** Paulina Borówka, Łukasz Pułaski, Błażej Marciniak, Beata Borowska-Strugińska, Jarosław Dziadek, Elżbieta Żądzińska, Wiesław Lorkiewicz, Dominik Strapagiel

**Affiliations:** 1 Department of Anthropology, Faculty of Biology and Environmental Protection, University of Lodz, 12/16 Banacha Street, 90–237 Łódź, Poland; 2 Department of Molecular Biophysics, Faculty of Biology and Environmental Protection, University of Lodz, 12/16 Banacha Street, 90-237 Łódź, Poland; 3 Institute of Medical Biology, Polish Academy of Sciences, 106 Lodowa Street, 93-232 Łódź, Poland; 4 Biobank Lab, Faculty of Biology and Environmental Protection, Department of Molecular Biophysics, University of Lodz, 14 Pilarskiego Street, 90-231 Łódź, Poland; 5 BBMRI.pl Consortium, 147 Stabłowicka Street, 54-066 Wrocław, Poland

**Keywords:** ancient DNA, aTB, ancient tuberculosis, NGS

## Abstract

**Background:**

Recent advances in ancient DNA studies, especially in increasing isolated DNA yields and quality, have opened the possibility of analysis of ancient host microbiome. However, such pitfalls as spurious identification of pathogens based on fragmentary data or environmental contamination could lead to incorrect epidaemiological conclusions. Within the *Mycobacterium* genus, *Mycobacterium tuberculosis* complex members responsible for tuberculosis share up to ∼99% genomic sequence identity, while other more distantly related *Mycobacteria* other than *M. tuberculosis* can be causative agents for pulmonary diseases or soil dwellers. Therefore, reliable determination of species complex is crucial for interpretation of sequencing results.

**Results:**

Here we present a novel bioinformatical approach, used for screening of ancient tuberculosis in sequencing data, derived from 28 individuals (dated 4400–4000 and 3100–2900 BC) from central Poland. We demonstrate that cost-effective next-generation screening sequencing data (∼20M reads per sample) could yield enough information to provide statistically supported identification of probable ancient disease cases.

**Conclusions:**

Application of appropriate bioinformatic tools, including an unbiased selection of genomic alignment targets for species specificity, makes it possible to extract valid data from full-sample sequencing results (without subjective targeted enrichment procedures). This approach broadens the potential scope of palaeoepidaemiology both to older, suboptimally preserved samples and to pathogens with difficult intrageneric taxonomy.

## Background

A rapid population growth initiated in the Neolithic period, connected with the domestication of animals and increase of human sedentism, played a key role in pathogen transmission within the so-called first epidaemiological transition [1–4]. The identification of infectious diseases and selection of unique fingerprints of their causative agents, especially those derived from skeletal elements, are still of great interest for palaeopathologists and anthropologists, which is evidenced by the range of available analysis methods. Members of the *Mycobacterium tuberculosis* complex (MTBC) are genetically very closely related and are causative agents for one of the oldest human infectious diseases—tuberculosis (TB). It is a disease that may leave lesions on patients' bones, enabling a diagnosis based on bone morphology [5]. The main problem of palaeopathological diagnoses based solely on dry bones is that TB-related bone changes are often nonspecific. The most reliable skeletal indicator of TB is destructive lesions in thoracic and lumbar spine sections, which can lead to destruction and collapse of vertebral bodies, resulting in spinal kyphosis, or gibbus, known as Pott's disease [5–7]. However, there are several pathological conditions that could mimic TB in dry bone, leading to erroneous diagnosis, especially when they affect the spine (Supplementary Table S1). Although their differential diagnosis from TB is well known in palaeopathology, it could be problematic to use it in analysis of often poorly preserved archaeological human remains [8, 9]. Diagnoses based on bone lesions in other regions of the skeleton are even more tentative; these are primarily based on osteomyelitis of the joints (most commonly the hip and knee but also ankle and elbow) and periosteal reactive lesions (mainly in the ribs or diaphysis of the long bones, including tubular bones of the hands and feet in children [6, 9]). Bone lesions from TB in nonspinal locations may be indistinguishable from those of other etiologies [5, 6]. Last, morphological studies of bones do not permit detection of many individuals affected with TB in past human populations: data from the pre-antibiotic era show that bone changes occur only in ∼3–7% of individuals with active TB [9].

Since the 1990s, new possibilities to diagnose TB in archaeological specimens have arisen, offered by the detection and analysis of mycobacterial DNA and other biomolecules specific to MTBC at the molecular level [10–21]. A common complication in molecular studies for ancient MTBC detection is the presence of DNA and other metabolites from the whole microbiome of the individual whose remains are being analysed, as well as from environmental bacteria that have colonized the skeleton post-mortem [22, 23]. These contaminants might include mycobacteria other than *M. tuberculosis* (MOTT), some of which are prevalent in the environment, while others are associated with clinical cases of non-tuberculosis diseases [22, 24–26]. It should be emphasized that members of MTBC themselves are characterized by a particularly high sequence similarity [27, 28], which often leads to unsurmountable difficulties in distinguishing them on the molecular level.

Detection of cell wall components such as mycolic, mycocerosic, and mycolipenic acids [13, 15, 18, 19] with matrix-assisted laser desorption/ionization tandem time of flight, which presents profiles specific for MTBC, is considered a reliable method to identify ancient causative agents in human archaeological samples. Initial attempts to use mass spectrometry to detect cell wall lipids were shown to yield erroneous results in some cases [15, 29, 30]. In more recent studies, the combination of cell wall lipid analysis with genetic markers showed significant improvement in discriminative ability for ancient mycobacteria [31, 32]. PCR, followed by gel electrophoresis, is still a popular method for detection of MTBC ancient DNA (aDNA) in human samples such as bones and teeth [32–34], mummified soft tissues [35, 36], or calcified pleura [10]. Known cases of TB disease proven on the basis of aDNA derived from human material are as old as 9000 BC [37], through the Iron Age [38] and up to modern times [39]. However, PCR-based methods have not been without controversy due to the possibility of cross-contamination, as well as limitations of selection of proper sequences. While repetitive insertion sequences, e.g., IS6110 and IS1081, are widely used and sometimes considered to be a biomarker specific to MTBC bacteria [34], the current consensus recommends strong caution in their use due to their presence in MOTT bacteria. Those commonly used markers have even been found to occur in soil mycobacteria [40–45], and even weak homology can cause false-positive PCR results for unrelated microbes [40, 46].

Recently, next-generation sequencing (NGS) methods were introduced for detection of causative agents of ancient diseases [47, 48], including MTBC, with or without pre-enrichment of MTBC aDNA [49–52]. The increasing quantity of data generated by NGS and the efficiency of non-Sanger-based sequencing platforms necessitates a new approach in processing tools: suitable bioinformatic pipelines are required for reliable DNA analysis of ancient causative agents. Similar to PCR, where the use of only short conserved regions considered to be specific for MTBC may lead to false-positive results, improper analysis of NGS data can misinterpret sequences from modern known or unknown environmental *Mycobacteria* that are present in ancient human skeletons [26]. New analytical tools for more unequivocal answers to questions of identification and differentiation of ante-mortem causative from post-mortem non-causative microbial agents are urgently needed. Application of specifically designed *in silico* (bioinformatical approach) verification methods for improved downstream processing of molecular fingerprint data from ancient samples is necessary for drawing conclusions on clinical prevalence and epidaemiology of pathogenic mycobacteria in history. Here we present an improved strategy for specific identification of bacteria from the MTBC in ancient non-enriched NGS data. The main purpose of this study was to design an unbiased genomic marker alignment query composed of sequences belonging strictly to MTBC members. Therefore, we present a workflow including appropriate bioinformatic alignment algorithms and statistical tools that allowed the identification of TB causative agents, using fragment length variation to balance selectivity (species specificity) with sensitivity of detection.

## Sample Description

Ancient bone samples came from the skeletal remains of 28 individuals representing 2 Neolithic populations from the Kujawy region in central Poland: the Middle Neolithic Brześć Kujawski Group of the Lengyel culture (BKG), dated to ∼4400–4000 BC (26 individuals), and the Late Neolithic Globular Amphora culture (GAC), dated to ∼3100–2900 BC (2 individuals), previously described by Borowska-Strugińska et al. [18] and Lorkiewicz et al. [53] (Supplementary Table S2). The skeletons come from 2 archaeological sites, BK 3 and BK 4, which represent relics of a settlement and cemetery of the BKG culture with some secondary objects within them, like the GAC grave. Both sites overlap each other; thus, soil conditions and diagenetic agents were similar for all skeletal remains analysed. Bone material was taken mainly from vertebral bodies of individuals with well-preserved skeletons. One of 2 individuals belonging to the GAC revealed bone lesions consistent with Pott's disease. BKG samples provided more ambiguous evidence of skeletal lesions. One individual showed destructive lesions of the thoracic and lumbar vertebrae with central collapse of the vertebral bodies, which may indicate tuberculous spondylodiscitis. Three other individuals of this population revealed only relatively mild and nonspecific inflammatory bone changes in the postcranial skeleton, which were located on the internal surface of the ribs, tibia and femur shafts, as well as foot bones.

## Analyses

### Reference target construction (alignment target)

As our main reference sequence, we used the most commonly applied modern laboratory strain of *M. tuberculosis* (MTB), H37Rv, for which the whole genomic sequence is available. To select a subset of this reference sequence as an alignment target providing enhanced specificity for TB-causing agents (MTBC members), we first derived a set of all coding DNA sequences (CDSs) from the H37Rv genome using the RAST tool [54]. These 4,360 sequences were screened using the BLAST tool (Megablast) at the National Library of Medicine sequentially against 12 available genomic sequences of selected MOTT: *M. kansasii, M. avium subsp. paratuberculosis, M. ulcerans, M. smegmatis, M. fortuitum, M. haemophilum, M. marinum, M. simiae, M. asiaticum, M. xenopi, M. phlei*,and *M. abscessus*. Any detected similarities (gapless alignments >10 bp) between an H37Rv CDS and any MOTT genomic sequences resulted in the exclusion of this CDS from the result dataset, which was therefore restricted to sequences fully specific for MTBC, having no homologs in any MOTT genome. The resulting set of sequences, hereafter the Borówka et al. alignment target, consisted of 1,534 CDSs with total sequence length of 0.814 Mb. Because no sequences from other MTBC species were used at this stage, and it is known that they exhibit up to 99.9% nucleotide sequence similarity [55], the constructed alignment target cannot be considered specific only for MTB, but rather for the whole MTBC; this is justified in epidaemiological studies on ancient samples by the need to include all clinically equivalent causative agents for the same disease entity: TB. For comparison purposes, we prepared and used 2 literature-derived, knowledge-based H37Rv sequence subsets as alternative alignment targets: the ∼0.046 Mb sequence used for capture enrichment in Bouwman et al. [52] for sequencing mycobacterial samples from a 19th century skeleton, hereafter called the Bouwman et al. alignment target, and the 2 genes (*katG* and *mpt40*, total length 0.004 Mb) listed as MTBC-specific among the capture enrichment probes used by Bos et al. [50] for sequencing mycobacterial samples from 11th−13th century Peruvian skeletons, hereafter the Bos et al. alignment target. All the reference sequences were prepared for alignment by indexing with the suffix array−induced sorting algorithm, implemented in the BWA software package.

Because the construction of the Borówka et al. alignment target was based on elimination of sequences similar to other mycobacterial species, we reasoned that the performance of an alignment target is directly linked to the number of similarities between the MTB genome and other potentially interfering mycobacterial species (both ancient and environmental) present in the ancient host-derived sample. In order to quantify this, we subjected the publicly available genome sequences of *Mycobacterium* species to an *in silico* procedure to generate collections of short sequences broadly analogous to authentic NGS reads. Including reads below a certain length in similarity analysis of ancient microbial DNA leads to non-specific matches (for both evolutionary and statistical reasons); this threshold is usually arbitrarily set to ∼30 bp, but a broader analysis might make it easier to construct a reliable algorithm for detection of specific ancient pathogens. Therefore, in our further analysis of both reference and authentic ancient NGS sequences we extracted groups (bins) of non-human sequences over several length thresholds: ≥20, ≥25, ≥30, and ≥35 bp, to enable a thorough analysis of specificity gain upon increase in minimal sequence length. For reference *Mycbacterium* genomes, k-mers of specified length (corresponding to the lower limit of read length for NGS bins: 20, 25, 30, or 35) were filtered against the human genome assembly hg19, and the resulting “short read” collections were aligned to the full MTB reference genome or its selected subsets (Borówka et al., Bouwman et al., and Bos et al. alignment targets). Table 1 shows the respective number of genomic k-mers from MTBC and MOTT species that match the MTBC alignment targets, as well as the total lengths of assayed genomes for comparison. Because the various subsets of the MTB genome differ in length and thus the probability of random match increases with target length, we standardized the obtained data by presenting it as a percentage of k-mers from a given mycobaterial genome that match the alignment target, divided by the ratio of target length to the full MTB genome length (genomic coverage of the target). These values, which are an inverse measure of alignment target specificity (they increase if more “reads” from a species that is not MTB or MTBC can be mistaken for MTBC), are presented in Table 1. As a reference, the MTB genome itself was also subjected to this procedure—obviously, the match percentage values are almost 100% here. Several conclusions can be drawn from these data: first, it is obvious that selecting longer reads (in this case longer k-mers) for comparison increases specificity, with reads ≥30 bp long optimal for specific identification of the MTBC, reflecting a common consensus in the field. However, it is important to note that shorter reads still add important information to the analysis because the rate of specificity increase (decrease in matching read percentage with increase in read length) varies between species (i.e., some species have longer stretches of highly similar sequence to MTB). For example, while *M. smegmatis* has a very high match percentage to the Borowka et al. alignment target at low read length, this is rapidly lost at longer (more genuine) read lengths; the opposite is true, e.g., for *M. marinum*. It is a derivation of the evolutionary history of the genus, but in this case also a practical caveat for further interpretation of sequence matches in actual aDNA samples. Moreover, the specificity of various alignment targets varies, with the Borówka et al. target being consistently the most specific (for longer k-mers) for distinguishing MOTT, while it is (by design) not well suited to distinguishing other members of the MTBC from MTB itself.

**Table 1:** Number of genomic k-mers from MTBC and MOTT members after initial hg19 clearing step matching selected targets, with k-mer length distinction (≥20, ≥25, ≥30, ≥35 bp), with estimation of percentage of k-mers from a given mycobaterial genome matching the *M. tuberculosis* genome for query length **≥**30 and **≥**35

			Query k-mer length ≥20					Query k-mer length ≥25					Query k-mer length ≥30					Query k-mer length ≥35				
**Species group**	Alignment target	Genome length (bp)	Sequences mapped to MTB genome (%)	Full genome	Borowka et al.	Bos et al.	Bouwman et al.	Sequences mapped to MTB genome (%)	Full genome	Borowka et al.	Bos et al.	Bouwman et al.	Sequences mapped to MTB genome (%)	Full genome	Borowka et al.	Bos et al.	Bouwman et al.	Sequences mapped to MTB genome (%)	Full genome	Borowka et al.	Bos et al.	Bouwman et al.
**MOTT**	*M. leprae*	3,268,203	3.19	140,922	19,736	101	1,683	4.88	215,257	4,349	85	2,240	2.61	115,138	1,430	26	1,201	1.45	63,860	543	6	715
	*M. abscessus*	5,067,172	5.26	232,228	46,530	158	2,915	2.87	126,769	2,816	103	1,890	1.39	61,160	283	46	1,065	0.75	33,175	62	14	644
	*M. smegmatis*	6,988,209	11.19	493,570	107,339	543	6,917	5.68	250,537	7,793	340	2,919	2.88	127,219	1,187	162	1,610	1.64	72,286	262	65	944
	*M. fortuitum*	6,254,616	8.48	374,030	77,785	291	5,208	5.22	230,382	5,940	131	2,774	2.69	118,483	958	40	1,534	1.53	67,463	236	16	916
	*M. phlei*	5,349,645	8.98	396,255	88,582	391	5,909	6.57	289,788	9,912	157	3,597	3.45	152,331	1,665	97	1,951	2.03	89,593	377	56	1,176
	*M. simiae*	5,938,797	9.33	411,677	80,142	339	5,414	9.51	419,641	12,734	197	4,578	5.35	235,800	3,904	93	2,702	3.15	139,050	1,450	33	1,575
	*M. asiaticum*	5,910,436	9.00	396,854	76,829	413	5,597	10.69	471,493	19,780	392	5,022	0.00	265,638	5,366	186	2,806	3.54	156,188	1,531	71	1,706
	*M. xenopi*	4,434,836	7.14	314,850	60,482	262	4,336	8.17	360,395	11,534	207	4,105	0.00	200,395	3,233	120	2,126	2.62	115,687	1,060	68	1,235
	*M. marinum*	6,660,144	9.48	418,304	82,499	466	5,715	14.08	621,166	52,438	707	6,366	7.88	347,459	16,301	266	3,465	4.49	198,076	4,208	88	2,046
	*M. ulcerans*	5,805,761	8.26	364,492	71,682	339	4,800	12.26	540,893	36,626	448	5,543	6.94	306,075	10,994	160	3,094	4.04	178,217	3,088	61	1,886
	*M. kansasii*	6,402,301	10.51	463,445	89,051	472	6,353	15.93	702,577	39,990	596	7,181	9.54	420,814	13,458	278	4,032	5.82	256,893	4,132	129	2,373
	*M. avium*	4,829,781	8.07	356,159	71,620	322	5,128	12.08	532,953	16,610	194	5,331	7.31	322,606	4,752	110	3,271	4.58	202,232	1,475	65	2,095
	*M. haemophilum*	4,235,765	7.08	312,375	52,214	274	4,137	13.05	575,862	22,641	540	6,284	7.98	352,034	8,023	374	3,703	4.94	217,744	2,893	254	2,322
**MTBC**	*M. caprae*	4,288,871	17.53	773,238	181,627	598	9,935	94.85	4,184,378	734,742	2,306	37,814	96.27	4,245,996	725,608	2,253	35,935	96.21	4,244,109	713,211	2,214	34,394
	*M. microti*	4,370,115	17.81	785,606	188,016	825	10,498	96.71	4,266,542	772,527	3,989	40,576	98.17	4,330,722	771,950	3,873	38,507	98.12	4,328,596	758,572	3,785	36,841
	*M. africanum*	4,389,314	17.87	788,161	186,939	850	10,494	97.15	4,285,645	764,554	4,038	40,740	98.63	4,350,937	764,150	3,893	38,685	98.60	4,349,503	751,103	3,796	37,018
	*M. bovis*	4,345,492	17.72	781,857	184,148	592	10,161	96.31	4,248,729	750,458	2,304	39,042	97.79	4,313,964	749,050	2,252	36,990	97.76	4,312,566	735 993	2,213	35,367
	*M. tuberculosis*	4,411,532	18.07	797,099	192,022	833	10,844	98.41	4,341,179	791,071	3,947	42,253	99.97	4,410,355	792,717	3,851	40,180	100	4,411,458	779,771	3,777	38,435

Because we intended to develop a highly specific screening test (based on low depth sequencing strategy) for verification of MTBC infection in Neolithic samples with an a priori relatively low degree of aDNA preservation, we decided on a statistical approach. Because any preserved ancient mycobacterial DNA would be only a fraction of total aDNA and it, in turn, would only be a fraction of total reads (the balance being the modern environmental metagenome), a balance between sensitivity and specificity in verifying this very low number of reads must be struck. In sedentary, communal populations MTBC infection tends to be epidemic in character, but in most individuals with latent infection the microbial load (and thus the probability of DNA survival in ancient samples) is relatively low and constant. Any similarity analysis based on sequence alignment will also invariably generate false-positive alignment hits; thus, it would be impossible to construct a test with sufficient statistical power to distinguish individuals genuinely free of ancient MTBC and those with average/modest latent infection. Therefore, we concentrated on the detection of outlier individuals with high microbial load (which may be later selected for enrichment-based further genetic analysis, such as phylogenetic studies or genome reconstruction), measured by the positive read ratio (the intrinsically very low ratio of reads matching the MTBC alignment target to all eligible reads). Based on the epidaemiology of MTBC infection, we assumed a quasi-normal distribution of positive read ratios in a randomly selected sample of ancient individuals, with outliers as candidates for active TB and for selection for more in-depth studies. Thus, our method was based on standardizing read ratio values to normal distribution parameters (arithmetic mean and standard deviation) and, as a further step in the detection algorithm for ancient tuberculosis (aTB), we applied a typical cut-off value of 1.5 × SD to detect outliers.

As a first stage of testing our screening approach on actual NGS data from ancient material, we used a control dataset based on published NGS results of confirmed TB-infected individuals: 18th/19th-century mummified bodies from a crypt in Vác, Hungary, described by Kay et al. [48]. The aim of that study was to reconstruct and analyse historical genome sequences of MTB, which resulted in sequencing results with high coverage. Because all these samples (26 bodies) were previously demonstrated by PCR to come from infected individuals [56], application of our screening procedure did not aim at distinguishing “positive” from “negative” samples but at validating the selection of individuals with highest microbial load (especially because some of them were sampled from 1–3 different parts of the body), at the same time enhancing specificity (vs MOTT). We used the Kay et al. dataset for verification of specificity of all applied alignment targets: Borówka et al., Bouwman et al., Bos et al., and the whole-genome sequence of *M. tuberculosis* H37Rv, with our algorithm aimed at detection of strongest aTB outliers. While application of the Borówka et al. target sequence (with 30 bp read length cut-off) detected 4 samples as outliers, they turned out to belong only to 2 individuals (bodies 68 and 92) (Supplementary Table S3). This validated our approach as a suitable method for selecting ancient samples with highest MTBC genetic material content, especially because, despite our alignment target consisting only of sequences specific exclusively for MTBC, it turned out that those 4 samples were also those that showed the highest ratio of aligned reads to the full MTB reference sequence (and thus the highest number of reads used to reconstruct the ancient genome) in the original study by Kay et al. (shown there in Supplementary Table S3). Moreover, only the 2 alignment targets prepared with both specificity and sensitivity in mind (Borówka et al. and Bouwman et al.) led to identification of all 3 samples from body 68 as outliers.

**Figure 1:**
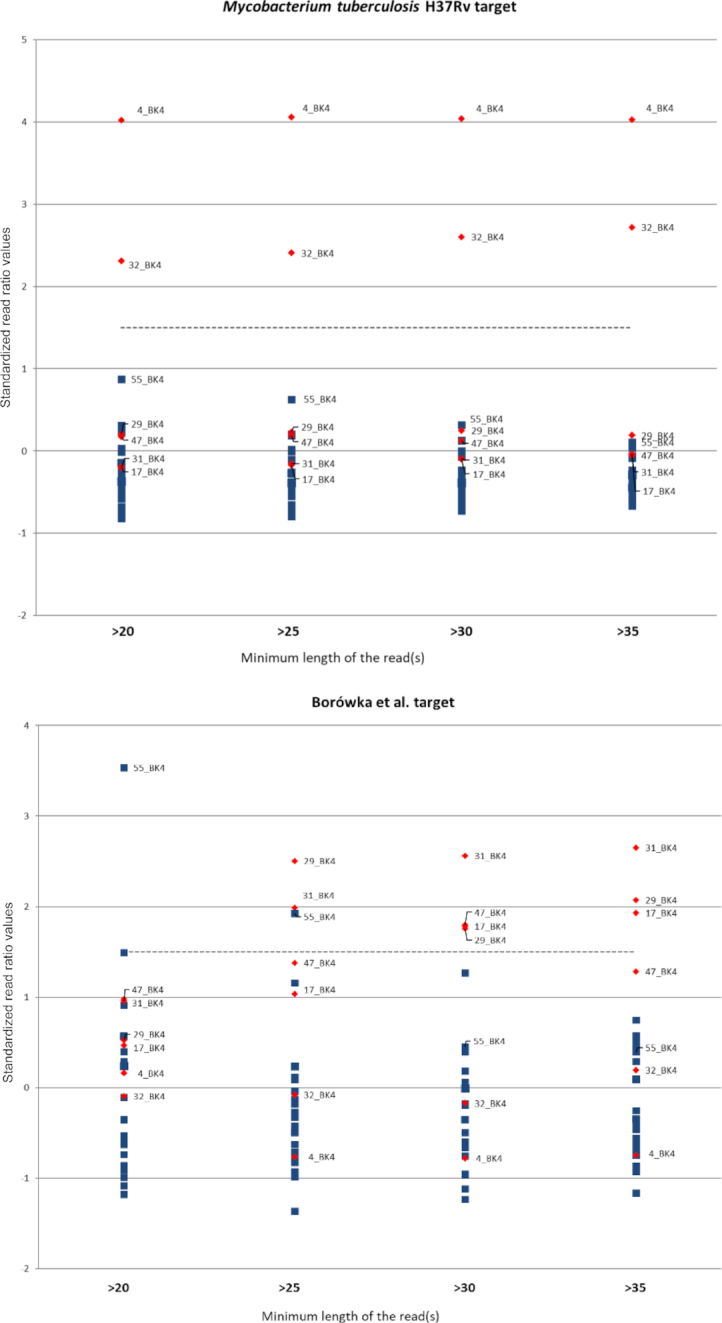

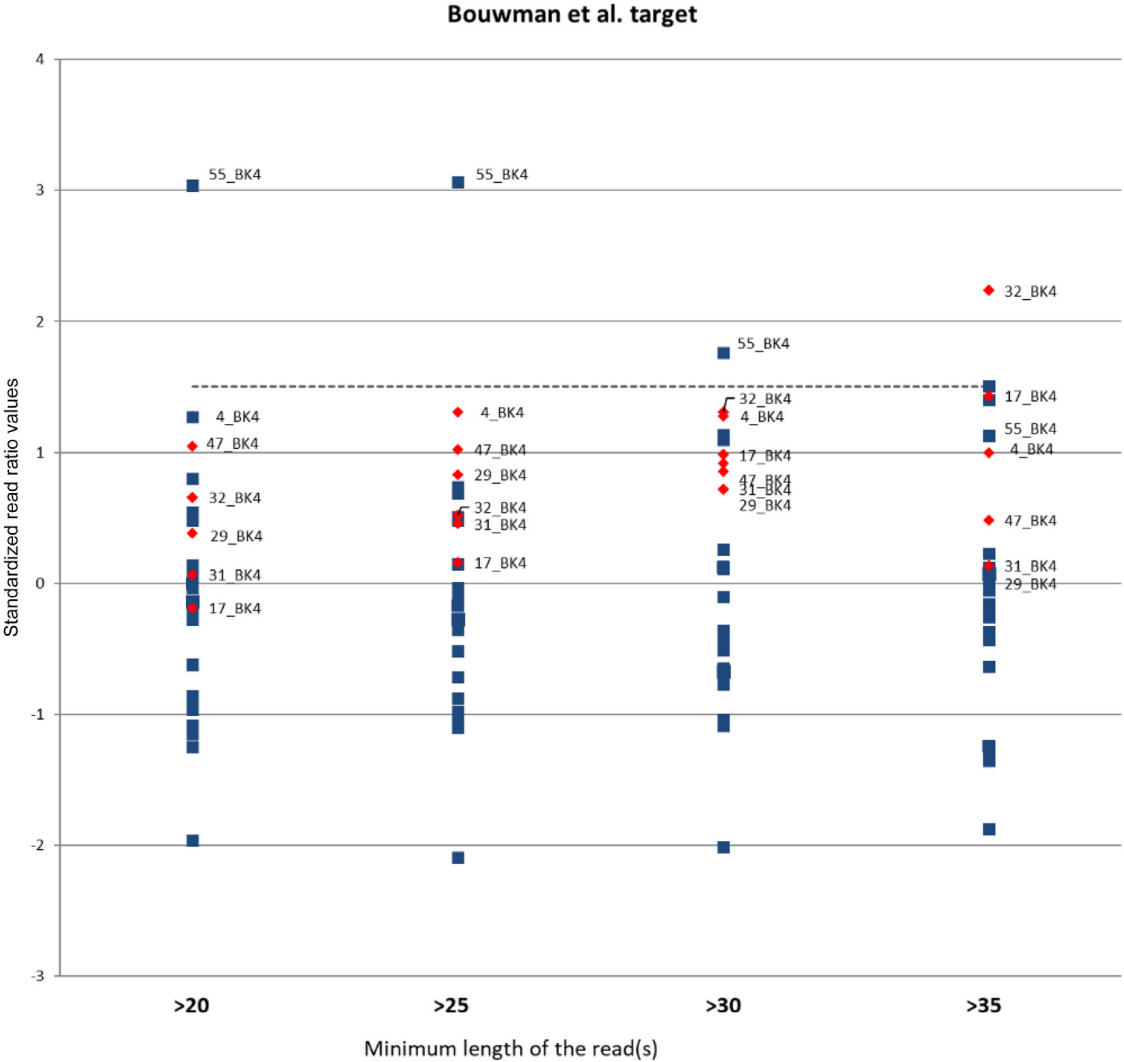
Changes in standardized ratio values in different read length bins and targets (red diamonds indicate outliers in *Mycobacterium tuberculosis* H37Rv and Borówka et al. targets in bin of reads ≥30 bp long).

Subsequently, we applied the full statistical approach (with all 4 NGS read length bins) and the 4 selected genomic alignment targets: full reference *M. tuberculosis* H37Rv genome (broadest possible target), 2 published targets consisting of rationally selected genes (applied previously to enrichment-based sequencing: Bouwman et al. and Bos et al.), as well as the novel specificity-tailored target (Borówka et al.), to the Neolithic samples from Brześć Kujawski. Table 2 presents the number of reads in each read length bin used for alignment with targets and statistical analysis, while Supplementary Tables S4–S7 show the alignment results as numbers and ratios of matching reads. Fig. 1 presents the results of statistical analysis as outlying standardized ratio values in different read length bins. Overall, the expected population structure of a majority of individuals with few positive reads and outlier individuals with an exceptional number of positive reads was confirmed. However, it is immediately obvious that the composition of outlier individuals depends strongly not only on the genomic alignment target but also on the minimum length of reads used for the alignment. There are individuals who remain positive (with a high relative ratio of reads aligning to the respective target) for all 4 length bins (e.g., 4_BK4 for the *M. tuberculosis* H37Rv target); i.e., the share of putative MTBC-derived sequences remains constant despite the decrease in number of analysed sequences and increase in sequence complexity. There are individuals who, despite being outliers for the bins including shorter reads, lose this status for the more restrictive bins (e.g., 55_BK4 for the Borówka et al. target); i.e., the majority of their MTBC-like sequences were of low complexity. In contrast, in some individuals the share of MTBC-like sequences increases above the cut-off value only for bins with longer reads (e.g., 31_BK4 for the Borówka et al. target); i.e., most specifically aligned fragments are relatively long. It is again apparent that because most of this change concerns reads between 20 and 29 bp in length, the optimal threshold for read aligning to a genomic target for specificity towards MTBC is ≥30 bp. Thus, the 3 individuals that exceed the threshold of 1.5× SD for the MTBC-specific Borówka et al. target (17_BK4, 29_BK4, and 31_BK4) are considered with high probability to be ancient cases of MTBC infection and merit selection for further in-depth studies by a more cost-intensive approach.

**Table 2:** Number of reads (per individual) used for alignment and statistical processing

Sample ID	Raw reads	Trimmed reads	Average read length	Non-human reads			
				>20	>25	>30	>35
1_BK4	17,507,911	17,038,725	57.6	16,977,024	16,902,603	16,378,765	15,191,086
4_BK4	18,816,573	18,215,498	51.7	18,095,660	17,960,494	17,086,604	15,246,279
6_BK4	16,322,105	15,815,995	55.0	15,551,094	15,427,193	14,682,610	13,220,243
7_BK4	2,231,650	2,160,395	59.7	2,102,955	2,095,297	2,047,913	1,936,435
9_BK4	14,974,057	14,503,433	53.5	14,240,738	14,085,752	13,149,549	11,600,503
11A_BK4	16,432,267	16,000,777	58.0	15,766,313	15,695,767	15,172,161	14,034,604
11B_BK4	18,522,995	18,078,222	55.7	725,913	718,941	674,747	597,601
12_BK4	23,116,936	22,273,434	55.6	21,272,850	21,151,065	20,156,692	18,073,071
14_BK4	17,849,685	17,383,629	58.8	17,310,864	17,235,014	16,752,835	15,595,926
15_BK4	16,062,102	15,607,381	58.2	15,539,859	15,460,941	14,915,585	13,881,414
17_BK4	14,980,797	14,496,468	58.1	14,426,404	14,372,805	14,078,235	13,247,545
18_BK4	24,217,412	23,575,201	59.1	23,370,869	23,281,268	22,704,123	21,306,454
21_BK4	11,890,953	11,500,254	60.1	11,271,958	11,237,968	11,021,676	10,439,448
22_BK4	17,996,717	17,498,339	58.8	17,417,850	17,365,274	17,013,067	16,007,094
25_BK4	17,560,698	16,997,518	57.7	16,888,515	16,816,770	16,375,850	15,237,575
29_BK4	8,994,172	8,724,285	58.1	8,683,928	8,642,230	8,393,680	7,800,006
31_BK4	20,427,813	19,941,632	58.4	19,741,741	19,684,774	19,309,226	18,187,574
32_BK4	35,100,769	33,926,405	54.9	33,754,943	33,623,260	32,780,233	30,194,531
33_BK4	24,501,712	23,719,299	58.3	21,669,095	21,595,959	21,031,538	19,569,420
34_BK4	16,453,473	16,047,224	57.3	14,901,123	14,842,998	14,421,402	13,376,818
47_BK4	18,736,966	18,155,651	55.6	17,998,648	17,903,991	17,174,561	15,478,180
55_BK4	17,435,264	16,904,284	48.0	16,768,595	16,530,082	14,886,541	12,170,210
65_BK3	17,465,925	16,921,732	50.6	16,735,483	16,587,671	15,466,034	13,185,810
71_BK4	17,919,758	17,434,181	50.4	17,086,979	17,017,135	16,549,441	15,441,174
72_BK4	16,355,009	15,952,974	57.9	15,874,302	15,812,384	15,444,022	14,541,576
73_BK4	17,050,731	16,578,547	57.8	16,270,896	16,212,738	15,778,509	14,632,126
77_BK4	14,044,420	13,478,859	56.0	13,390,126	13,322,735	12,866,845	11,763,625
78_BK4	17,004,599	16,352,717	60.1	16,250,859	16,164,585	15,758,397	15,027,226

Because the cut-off−based detection algorithm, while robust for the presented dataset, may be less suitable for other, less homogenous groups of ancient individuals, we also set out to construct an objective, parametric testing−based outlier detection algorithm. Because the main objective of our overall study is specificity of MTBC detection, we applied this algorithm to the original Borówka et al. genomic alignment target. On the basis of the observation that positive read ratio tends to depend monotonically on read length bin—either consistently increasing or decreasing for outlier individuals—we decided to calculate a monotonicity parameter. We first standardized positive read ratios as a percentage of average positive read ratio (without assumptions towards normal distribution; Supplementary Fig. S1) and then calculated ratios of these values for adjoining read length bins (≥25/≥20, ≥30/≥25, and ≥35/≥30 bp). The arithmetic mean of these values (Supplementary Table S8) depended on monotonicity of the studied relationship and had a normal distribution among individuals in our study. For outlier detection, we applied a 1-tailed critical *z*-value test on both tails on the sample. We consider the positive outliers (individuals with consistently increasing share of positively aligned reads with increasing read length) to be potential individuals with high MTBC loads, suitable for further analysis both by virtue of good mycobacterial genomic material preservation and high certainty of this material belonging to ancient MTBC. On the other hand, negative outliers may be either individuals with ancient MOTT infection (we suggest this as highly probable for 4_BK4) or samples with a high proportion of short, non-specific alignments, probably due to environmental contamination (most probably 55_BK4); to distinguish these 2 groups, a comparison with the more *Mycobacterium*-generic whole-genome alignment target is necessary (see below). This approach, while retaining the strong specificity of the cut-off approach, gains increased sensitivity due to inclusion of individuals with a high background of environmental sequences (low initial positive alignments in the short-read bin), which nevertheless retain specific long positively aligned sequences upon read length restriction, e.g., 21_BK4.

**Figure 2:**
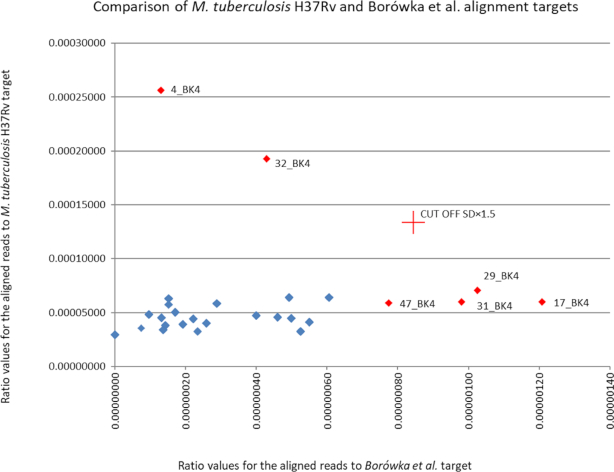
Comparison of alignment targets constructed with different assumptions (red diamonds indicate outliers in *Mycobacterium tuberculosis* H37Rv and Borówka et al. targets in bin of reads ≥35 bp long).

An immediately obvious result of our analysis was that the comparison of alignment targets constructed with different assumptions leads to surprisingly large differences in assignment of individuals. Aligning aDNA sequences vs the whole MTB genome resulted in identification of 2 strong outliers (4_BK4 and 32_BK4). The same 2 individuals were identified, albeit with a smaller divergence, by using the enrichment bait sequence set used by Bouwman et al. as alignment target. Because this subset of genomic sequences was originally selected for enrichment of lineage-distinguishing polymorphisms rather than for MTBC specificity, this result was expected and confirms the efficiency of the outlier detection method and ≥30 bp as optimal read length. On the other hand, our Borówka et al. genomic subset selected on the basis of MTBC specificity led to identification of 3 different individuals as outliers (17_BK4, 29_BK4, and 31_BK4), while 4_BK4 and 32_BK4 had positive read values close to average. This is even more conspicuous when positive ratio values for the 2 different alignment targets (whole-genome and specific subset Borówka et al.) are plotted against each other (Fig. 2). In our opinion this points to the broadly recognized risk of mistakenly identifying ancient infections caused by MOTT as TB based on the extensive similarity between the respective mycobacterial genomes. While restricting the alignment target leads to loss of sensitivity due to unavoidable an decrease in the absolute number of aligned reads, which is a significant problem for aDNA, it is offset by the increase in specificity of detection. This distinction is crucial for epidaemiological hypotheses where elimination of false-positive results is of paramount importance. We further show this by aligning our reads to the purportedly MTBC-specific target sequences selected by Bos et al. (sequences of only 2 MTB-specific genes), where increase of specificity leads to detection of the 29_BK4 individual, but the extreme loss of sensitivity linked to minuscule absolute number of reads (the highest number of positive reads in the ≥30 bp bin is 13; see Supplementary Table S7) leads to high experimental noise and low reliability of assignment of individuals, and it is not recommended.

Because for 2 individuals that were strongly enriched in mycobacterial sequences (4_BK4 and 32_BK4) we posit the existence of an ancient MOTT infection (because they do not score highly in comparison with the specific Borówka et al. alignment target), we decided to verify whether this assumption is supported by aligning the optimal read bin (≥30 bp) to full genomes of other mycobacterial species as targets. Indeed, as seen in Supplementary Fig. S2, those 2 individuals are also strong outliers in read ratio values after aligning to the *M. marinum* genome—moreover, when plotted against read ratio values for the MTB genome, it is apparent that they show higher similarity to *M. marinum* because they are located on the *M. marinum* side of the read ratio regression line. This finding validates our workflow in that it corroborates the usefulness of read length binning while further demonstrating the advantages of read aligning to targets selected for species discrimination (such as the Borówka et al. target) that allow for immediate flagging of suspicious samples with spuriously high absolute similarity to the MTB genome. We have also attempted to verify the possibility of distinguishing samples with predominantly ancient mycobacterial sequences from samples with recent environmental MOTT contamination by performing mapDamage analysis. MapDamage analysis shows that the low absolute number of reads that map to all MTB alignment targets (including the full MTB genome) in the case of our samples prevents us from drawing meaningful conclusions in this regard (even for the samples with highest read numbers, 4_BK4, 32_BK4, 17_BK4, 29_BK4, and 31_BK4). For general confirmation of ancient status of analysed reads, MapDamage analysis was performed for human sequences (aligning to the human genome build 37) and is presented in Supplementary Fig. S3 for all 6 individuals with potential MOTT and MTBC infections. Because the samples with potential MOTT infection (4_BK4 and 32_BK4) included a substantial number of reads that aligned to the *M. marinum* genome (Supplementary Table S9), we were also able to perform MapDamage analysis for these reads (Supplementary Fig. S4), confirming the ancient character of mycobacterial sequences.

## Discussion

The evolutionary and ecological complexity of mycobacteria, including the existence of a group of closely related pathogens known as the MTBC, consists of a large number of more distantly related human and animal pathogens causing diseases other than TB, and an abundance of free-living (including soil- and water-borne) mycobacterial species in the environment. These all contribute to the difficulty in the unequivocal determination of aTB on the basis of MTBC aDNA. Present-day palaeoepidaemiology uses tools of classical biological anthropology as well as modern clinical diagnostics at the molecular level. Morphological diagnosis of tuberculosis is based on certain bone changes, especially those described as Pott's disease. This approach is not optimal from the point of view of sensitivity because bone lesions are present only in 2% of all cases of TB infection and 10–20% of cases of extrapulmonary TB [41, 57]. The specificity of this tool is also relatively low: even in the case of Pott's disease, which is regarded by palaeopathologists as the pathognomonic skeletal signature of TB, there are several lesions that may be difficult to differentiate from TB in archaeological skeletal remains. In spite of these limitations, osteological analysis is often the main starting point of a study and cannot be disregarded. However, in our study the occurrence of bone lesions that could be linked in any way with TB did not correlate with the results of our genetic analyses. There are 2 possible explanations for this fact. First, the bone changes were not caused by TB, which is in accordance with a lack of pathognomonic characteristics of the disease on the skeleton alone, as was clarified before; it applies primarily to the graves 12_BK4, 18_BK4, 47_BK4, and 73_BK4. It may also be that the preservation of MTBC aDNA was too poor to pass the sensitivity/specificity threshold of the method proposed here.

Among molecular techniques that are used for diagnosis of aTB cases, both biochemical methods based on mass spectrometry and PCR amplification of marker sequences have been successfully used in the literature, e.g., for preliminary description of the Hungarian mummies used subsequently to reconstruct aTB genomes [48, 56]. However, both these groups of methods display a number of drawbacks that make them less useful in an ancient epidaemiological context than in a contemporary one: environmental contamination from modern soil mycobacteria can overwhelm both traces of ancient MTBC mycolic acids and less specific PCR amplicons, while strong care must be taken to prevent in-laboratory cross-contamination with genuine MTBC samples. Therefore, NGS has a number of advantages in diagnosis of aTB, having the potential to be both highly sensitive and highly specific; but the balance between sensitivity and specificity depends on the selection of reference genomic sequences and crucially on the method of alignment. A large quantity of generated data allows ancient mycobacteria to potentially be detected selectively, unequivocally, and semi-quantitatively, while making possible additional analyses such as preservation period−related DNA damage pattern detection (e.g., mapDamage [58, 59], phylogenetic analysis of genetic kinship [50], or even full genome reconstruction [48]). Owing to small absolute amounts of actual ancient pathogen DNA in most types of human body samples, a common approach is to use pre-sequencing enrichment (usually using probe capture, e.g., [50]). Only in bodies preserved in exceptional, isolated conditions, such as the Hungarian mummies from an 18th century crypt, was a non-enriched metagenomics approach used [48]. Use of enrichment techniques strongly increases sensitivity but comes with its own drawbacks (apart from increased cost), the most relevant of which is the need to pre-design a set of sequences (probes or primers) that will define and limit the scope of subsequently obtained NGS data. A full metagenome approach is often more relevant when dealing with a highly ancient sample like in the present study, when neither the infection prevalence nor the pathogen identity are known to any precision and a preliminary NGS study is needed for formulation of specific hypotheses and pre-selection of individuals for further analysis.

However, in the case of ancient MTBC (especially samples >1,000 years old), specificity is a more important consideration than sensitivity. While modern MTBC contamination in the laboratory is a risk factor, it would not mask ancient data in a semi-quantitative study and would be obvious if DNA damage analysis were performed. A more important consideration is the possible presence of ancient MOTT, which can be unpredictably genetically similar to MTBC. The sources of these MOTT can be either soil contamination (including dead animals), which could have happened at any time since inhumation (preventing reliable elimination by DNA damage analysis), or actual ancient MOTT that were pathogenic/infectious/commensal to ancient humans. Thus, the design of the sequencing analysis workflow has to take into account the need to filter out unknown related sequences that are not derived from MTBC; this was the main rationale behind the design of our study. While contamination with mycobacterial sequences within the laboratory (amplicons, genuine *Mycobacterium* DNA) can be prevented by correct workflow (e.g., separation of pre- and post-PCR areas), equipment, and strict procedures, contamination by environmental DNA is inescapable and has to be taken into account in the case of archaeological bone samples preserved by inhumation. Because for ancient samples direct contact of bones with the environment has lasted for a very long time (unlike more recent samples from vault inhumation), mycobacterial DNA derived from environmental (soil) MOTT can have undergone accretion in bones throughout this period, with some of it ancient enough to be indistinguishable in terms of location and state of preservation from DNA of infectious microbes buried with the body. All MTBC are obligate pathogens and thus are an unlikely source of environmental contamination of ancient samples. Therefore, for preliminary identification of potentially interesting samples in ancient inhumated bones, specificity in methods of detection of ancient infectious agents from this group should be developed towards exclusion of MOTT, with distinction between members of MTBC as a secondary, much less important goal. Because MTBC also share a very high proportion of coding sequences, achieving specificity for MTB species could occur only by drastically limiting the size of the reference marker sequence, thus leading to very low sensitivity, especially for usually highly degraded aDNA. Moreover, the division of MTBC into lineages is not entirely concordant with classical taxonomic division into species, so attempting an artificial distinction between some lineage groups based on accumulated NGS data would not be recommended. Our approach is designed as a relatively low-cost, first-pass classification of ancient samples based on whole-metagenome NGS data. When a highly specific method like the one we propose is used to identify likely ancient MTBC infection, potential lineage determination or any other phylogenetic studies (in pre-selected samples) should proceed by other methods developed specifically for this purpose, based on the presence of lineage-specific polymorphisms (with the caveat that enrichment for specificity-related sequences before NGS will certainly lead to loss of the majority of phylogenetically important loci, so a full metagenomic sequencing round with sufficient coverage is inevitable).

We postulate that a combination of read length−based genomic alignment analysis and a careful knowledge-based selection of the alignment target makes it possible to achieve relatively high specificity of aTB detection against all potential false-positive sources. Therefore, a robust tool for specifically identifying NGS-derived sequences that belong to ancient MTBC with high confidence is a priority task in molecular palaeoanthropology. Even more relevant to palaeoanthropological studies, confusion between MOTT and MTBC can lead to spurious identification of ancient individuals as TB sufferers or carriers, invalidating conclusions relevant to palaeoepidaemiology. We demonstrate that read length selection is not only highly relevant (as has been shown before and by us, only reads above ∼30 bp can be used with high confidence), but when a statistics-based approach to multiple length thresholds is used, it can yield a substantial increase in specificity of MTBC identification. At the same time, selection of a pre-filtered alignment target, with combined knowledge-based (selection of transcribed sequences) and automated (exclusion of sequences aligning with MOTT genomes) delineation of MTBC-specific sequences (which we call the Borówka et al. target), makes it possible to perform in-depth specificity analysis by comparing the alignments of *in silico* fragmented mycobacterial genomes (mimicking actual NGS data). Combining the novel alignment target and the read length binning approach, we were able to select with high confidence 3 ancient individuals with probable ancient MTBC infection and 2 further individuals with highly probably ancient mycobacteriosis caused by MOTT (which would be misidentified as TB if another alignment target or excessively short reads were taken into account). Of course the limitations of our data make these identifications preliminary and another round of directed (e.g., enrichment-based) sequencing would be required both for positive identification of the infectious agent and for potential phylogenetical analysis of its spatial and/or temporal kinship. However, in our case read length analysis allowed us to suggest *M. marinum* as the potential ancient infectious agent based on statistical analysis; obviously, positive confirmation of this diagnosis would require tools that are currently unavailable such as proven *M. marinum*−specific enrichment probes, as well as a much better sequence coverage than could be achieved in a preliminary study (Supplementary Fig. S2). Still, this possible pathogen identification is not at odds with the archaeological context because the inhumation site is next to a lake (Smetowo) and within a geographical region rich in post-glacial lakes (Kujawy), so some individuals could have had habitual contact with fish. Our combined procedures used robust tools but cannot be treated as definite proof. Our samples are relatively old (in comparison to most other aTB cases studied by molecular means before), and thus the absolute read numbers from an unbiased NGS approach are low. We demonstrate that this disadvantage makes it relatively difficult to perform DNA damage analysis (except for samples with a very high absolute number of reads). However, we provide a consistent proof of concept for a tool that allows relatively cheap and unbiased selection of samples (e.g., individuals) for further analysis, e.g., by enrichment capture NGS. Thus, we suggest that it is possible to use global NGS results from ancient samples as an economical pre-screening tool for more complex methods, while applying bioinformatic tools to maximize the number of reliable conclusions that can be drawn from a limited dataset.

## Methods

### Ancient DNA extractions

A dedicated aDNA sample preparation facility at the University of Lodz was used, taking standard precautions to avoid any contamination. All disposable materials, buffers, water, clean room surfaces, and bone material were UV-irradiated for ≥30 minutes before any subsequent steps were taken. The fragments of bone material were isolated using Dremel disks (Mount Prospect, Illinois, USA), surface-cleaned, UV-irradiated for 7.5 minutes on each side, and ground into a fine powder, and further used for DNA extraction procedures following the protocol of Dabney et al. with modifications [60–62]. Ancient DNA was successfully isolated from all bone samples (See Supplementary Fig. S3). Illumina libraries were prepared in a separate facility, according to the Meyer et al. protocol [63] with modifications proposed by Gamba et al. [60] without UDG (uracil DNA glycosylase) treatment of the samples. All libraries were subjected to the screening NGS on the Illumina Nextseq 500 platform (100 bp single-end sequencing), yielding between 2.2 and 33.9 million reads per individual (median number of reads after incomplete and truncated read trimming, 16.9 million reads per individual; Table 2). This dataset contains ancient human sequences from the deceased individuals, ancient microbial sequences from parasites, pathogens, commensals, or symbionts of the deceased individuals, as well as genomic sequences from environmental organisms (mainly microbes but also potentially higher eukaryotes), to which the skeletal remains were exposed post-mortem.

### Bioinformatical procedures

Raw NGS reads were subjected to standard quality processing such as trimming and adapter sequence removal (–q 30 –phred33 –illumina –length 20), using the Trim Galore! software package [64]. Because the predominant expected type of sequence in skeletal samples is ancient human genomic DNA and its presence would unnecessarily complicate our analysis, the read datasets were subsequently subjected to filtering by alignment to the standard (hg19) human genome reference sequence. This alignment was performed using the BWA_aln algorithm (–n 0.04, –l 1000), with duplicate removal, using the AGAT software tool—ocwrapper3mt.py script [65]. Any read that aligned without gaps within the default mismatch rate (dependent on sequence length, e.g., 2 mismatches per 17 bp) was eliminated from the sample dataset. Subsequently, separate sub-datasets (bins) of reads were generated on the basis of (trimmed) read length: minimal read length threshold ≥20, ≥25, ≥30, and ≥35 bp. These datasets were used for alignment to reference targets. These procedures were applied also to the Kay et al. dataset, used for the Borówka et al. method verification.

Estimation of terminal base deamination damage pattern was done by using mapDamage2.0 analysis with specifying a length (–l) of 75  bp (Supplementary Figs S3 and S4).

### Query sequence preparation

Eighteen selected reference *Mycobacterial* genomes were used: *M. abscessus,M. africanum, M. asiaticum, M. avium, M. bovis, M. caprae, M. fortuitum, M. haemophilum, M. kansasii, M. leprae, M. marinum, M. microti, M. phlei, M. simiae, M. smegmati, M. tuberculosis, M. ulcerans*,and*M. xenopi*. Among these, 5 were of MTBC: *M. africanum,M. bovis, M. caprae,M. microti*, and *M. tuberculosis*. Nucleotide sequences of each organism were subjected to fragmentation with FA_TOOL script (small_tool.py) [66], respectively, for 20, 25, 30, and 35 bp-long fragments and allocated in the same manner to length bins. Furthermore, fragmented genomes were used for specificity testing of each constructed target, which solved the problem of very short and non-specific fragments with threshold estimation.

### Verification of specificity and sensitivity of NGS screening method

Due to the lack of available NGS data of positive MTB cases, we tested *in silico* methods by using the Kay et al. dataset (PRJEB7454), derived from Hungarian mummy tissue microbiome sequencing. SRA files for each sample were identified and downloaded, and further fastq files were passed through trimming with deprivation of the adapter sequences [65]. Raw sequencing files were conducted to human genome reference sequence (hg19) filtration in spite the fact that host DNA material could be dominant in the sample. Alignment was performed to the tested targets *M. tuberculosis* H37Rv, Borówka et al., Bos et al., and Bouwman et al. using the AGAT software tool [65]. Statistics for each individual are presented in Supplementary Table S3. Summarized results of aTB cases from Brześć Kujawski are included in Supplementary Tables S4–S7.

### Statistical processing and parametric testing–based outlier detection algorithm

Collected unmapped sequences from the original dataset, as well as from the Kay et al. dataset, were aligned to constructed marker sequences: *M. tuberculosis*H37Rv, Borówka et al. (Supplementary Table S10), Bos et al., and Bouwman et al. with application of experimentally determined minimal read length threshold ≥17, ≥20, ≥25, ≥30, and ≥35 bp for detection of potential ancient MTBC cases. For detection of outlier individuals with high microbial load/positive read ratio, we standardized read ratio values to normal distribution parameters (arithmetic mean and standard deviation) and, as a further step in the aTB detection algorithm, applied a typical cut-off value of 1.5 × SD to detect outliers, postulating these to be candidates for active TB.

Based on the observation that positive read ratio tends to depend monotonically on read length bin—either consistently increasing or decreasing for outlier individuals—we decided to calculate a monotonicity parameter. We first standardized positive read ratios as a percentage of average positive read ratio and then calculated ratios of these values for adjoining read length bins (≥25/≥20, ≥30/≥25, and ≥35/≥30 bp). For outlier detection, we applied a 1-tailed critical *z*-value test on both tails of the sample. We consider the positive outliers (individuals with consistently increasing share of positively aligned reads with increasing read length) to be confirmed cases of active aTB infection (See Supplementary Tables S3–S7).

## Availability of supporting data and materials

The datasets supporting the conclusions of this article are available under the NCBI repository project “Identification of ancient tuberculosis in human archaeological remains” (accession No. PRJNA422903) including Biosamples and related SRA data. Other supporting data are available via the *Gigascience* database, GigaDB [67].

## Additional files

Supplementary Table 1. Differential diagnosis of tuberculosis-like lesions of the spine in paleopathology (after [5, 6]).

Supplementary Table 2. Summarized information about 28 individuals (BK4 and BK3 – Brześć Kujawski site 4 and 3; F- female, M – male), with detailed information about bone samples derived for aDNA analysis in column 5. Column 6 describe presence of observed lesions in the analyzed skeletons.

Supplementary Table 3. Summarized information about alignment results for Kay et al. (2015) M. tuberculosis positive Individuals, used as a verification of Borówka et al. bioinformatical approach in comparision to the other used targets.

Supplementary Table 4. Summarized information about number of reads within each read length bin that align to the Borówka et al. genomic target. AVG - arithmetic mean; STDEV - standard deviation; CUTOFF 1.5 - AVG×(1.5×STDEV).

Supplementary Table 5. Summarized information about number of reads within each read length bin that align to the Mycobacterium tuberculosis H37Rv genomic target. AVG - arithmetic mean; STDEV - standard deviation; CUTOFF 1.5 - AVG×(1.5×STDEV).

Supplementary Table 6. Summarized information about number of reads within each read length bin that align to the Bouwman et al. (2012) genomic target. AVG - arithmetic mean; STDEV - standard deviation; CUTOFF 1.5 - AVG×(1.5×STDEV).

Supplementary Table 7. Summarized information about number of reads within each read length bin that align to the Bos et al. (2015) genomic target. AVG - arithmetic mean; STDEV - standard deviation; CUTOFF 1.5 - AVG×(1.5×STDEV).

Supplementary Table 8. Arithmetic mean for calculated ratios (read ratios as percentage of average positive read ratio) for adjoining read length bins (≥25bp/≥20bp, ≥30bp/≥25bp and ≥35bp/≥30bp) for each individual.

Supplementary Table 9. Summarized information about number of reads aligned to the Mycobacterium tuberculosis H37Rv and Mycobacterium marinum (NC010612.1) genomic target.

Supplementary Table 10. Borówka et al. 1534 query sequences presented and used in this study for detection of Mycobacterium tuberculosis complex infected individuals.

Supplementary Fig. 1 Standardized positive read ratios as percentage of average positive read ratio for Borówka et al. target.

Supplementary Fig. 2. Comparison of alignment targets M. tuberculosis H37Rv and M. marinum genomes (red diamonds indicate outliers in Mycobacterium tuberculosis H37Rv and M. marinum all targets in bin of reads equal or longer than 30).

Supplementary Fig. 3. MapDamage patterns for samples indicated as a possible MOTT infection (a4_BK4, b32_BK4), and potential M. tuberculosis sufferers (c17_BK4, d29_BK4, e31_BK4, with additional sample f47_BK4) , based on human reads (aligning to human genome build 37) with length ≥30. Due to low number of non-human reads attributed to each sample after mapping step, it was not useful to analyse MapDamage patterns on reads aligning to M. tuberculosis genome. Blue color indicates G to A transitions, red color indicates C to T transitions per sample.

Supplementary Fig. 4. MapDamage patterns for samples indicated as a possible MOTT infection (a4_BK4, b32_BK4), based on reads with length ≥30 aligning to the M. marinum genome. Blue color indicates G to A transitions, red color indicates C to T transitions per sample.

giz065_GIGA-D-19-00014_Original_Submission

giz065_GIGA-D-19-00014_Revision_1

giz065_Response_to_Reviewer_Comments_Original_Submission

giz065_Reviewer_1_Report_Original_Submission Tanvi Honap -- 2/27/2019 Reviewed

giz065_Reviewer_1_Report_Revision_1 Tanvi Honap -- 4/8/2019 Reviewed

giz065_Reviewer_2_Report_Original_Submission Helen Donoghue -- 2/28/2019 Reviewed

giz065_Supplemental_Files

## Abbreviations

aDNA: ancient DNA; AGAT: Ancient Genomes Analysis Tool; aTB: ancient tuberculosis; BKG: Brześć Kujawski Group; bp: base pairs; CDS: coding DNA sequence; GAC: Globular Amphora culture; Mb: megabase pairs; MTBC: *Mycobacterium tuberculosis* complex; MOTT: Mycobacteria other than tuberculosis; NCBI: National Center for Biotechnology Information; NGS: next-generation sequencing; RAST: Rapid Annotation Using Subsystem Technology; SRA: Sequence Read Archive; TB: tuberculosis.

## Competing interests

The authors declare that they have no competing interests.

## Funding

The study was financed by the Polish Ministry of Science and Higher Education grant DIR/WK/2017/01 and by the Polish POPC Grant 02.03.01–00-0012/17–00 from the European Regional Development Fund.

## Authors' contributions

P.B. and D.S. conceived the study and were responsible for extraction of aDNA, preparation of NGS libraries, and NGS of samples. P.B., D.S., and Ł.P. analysed the data, discussed the results, and wrote the manuscript. Ł.P. participated in the statistical analysis and figure preparation. B.M. wrote and ran the AGAT primary analysis. B.B-S. participated in sample selection and preparation for the laboratory phase. J.D. and W.L. analysed the samples for pathological changes, participated in the study design, analysed and discussed the data, and participated in drafting the manuscript. E.Ż. participated in the study design, analysed and discussed the data, and participated in drafting the manuscript. D.S. coordinated studies and was responsible for the final version of the manuscript; all authors read and approved the final manuscript.
