# Supplementary material for: Screening methods for detection of ancient Mycobacterium tuberculosis complex fingerprints in next-generation sequencing data derived from skeletal samples
Source: Gigascience. 2019 Jun 20;8(6):giz065. doi: 10.1093/gigascience/giz065 (PMC6586198; doi:10.1093/gigascience/giz065)
Supplement: giz065_GIGA-D-19-00014_Original_Submission [file giz065_giga-d-19-00014_original_submission.pdf]

GigaScience

Screening methods for detection of ancient Mycobacterium tuberculosis complex fingerprints in NGS data derived from skeletal samples

--Manuscript Draft--

**Manuscript Number:**	GIGA-D-19-00014
**Full Title:**	Screening methods for detection of ancient Mycobacterium tuberculosis complex fingerprints in NGS data derived from skeletal samples
**Article Type:**	Research
**Funding Information:**	Ministerstwo Nauki i Szkolnictwa Wyższego (DIR/WK/2017/01) — Dr Dominik Strapagiel<br>Operational Programme Digital Poland (02.03.01-00-0012/17-00) — Mr Błażej Marciniak

Abstract:

Background

Recent advances in ancient DNA (aDNA) studies, especially in increasing isolated DNA yields and quality, opened the possibility of analysis of ancient host microbiome. However, this analysis could lead to numerous pitfalls, including spurious identification of pathogens based on fragmentary data or environmental contamination, leading to incorrect epidemiological conclusions. Within the Mycobacterium genus, MTBC (Mycobacterium tuberculosis complex) members responsible for tuberculosis share up to ~99% genomic sequence identity, while other more distantly related MOTT (Mycobacteria other than tuberculosis) can be causative agents for pulmonary diseases or soil dwellers. Therefore, reliable determination of species complex is highly relevant for interpretation of sequencing results.

Results

Here we present a novel bioinformatical approach, used for screening of ancient tuberculosis in sequencing data, derived from 28 individuals (dated 4400 - 4000 BC and 3100 - 2900 BC) from Central Poland. We demonstrate that cost effective next generation screening sequencing data (c.a 20M reads per sample) could yield enough information to provide statistically supported identification of probable ancient disease cases.

Conclusions

Application of appropriate bioinformatic tools, including an unbiased selection of genomic alignment targets for species specificity, makes it possible to extract valid data from full-sample sequencing results (without subjective targeted enrichment procedures). This approach broadens the potential scope of paleoepidemiology both to older, suboptimally preserved samples and to pathogens with difficult intrageneric taxonomy.

**Corresponding Author:**	Dominik Strapagiel<br>University of Lodz<br>Lodz, POLAND
**Corresponding Author Secondary Information:**	
**Corresponding Author's Institution:**	University of Lodz
**Corresponding Author's Secondary Institution:**	
**First Author:**	Paulina Borówka
**First Author Secondary Information:**	
**Order of Authors:**	Paulina Borówka

Powered by Editorial Manager® and ProduXion Manager® from Aries Systems Corporation

	Lukasz Pułaski
	Błażej Marciniak
	Beata Borowska-Strugińska
	Jarosław Dziadek
	Elżbieta Żądzińska
	Wiesław Lorkiewicz
	Dominik Strapagiel
Order of Authors Secondary Information:	
Additional Information:	
**Question**	**Response**
Are you submitting this manuscript to a special series or article collection?	No
**Experimental design and statistics**<br><br>Full details of the experimental design and statistical methods used should be given in the Methods section, as detailed in our Minimum Standards Reporting Checklist. Information essential to interpreting the data presented should be made available in the figure legends.<br><br>Have you included all the information requested in your manuscript?	Yes
**Resources**<br><br>A description of all resources used, including antibodies, cell lines, animals and software tools, with enough information to allow them to be uniquely identified, should be included in the Methods section. Authors are strongly encouraged to cite Research Resource Identifiers (RRIDs) for antibodies, model organisms and tools, where possible.<br><br>Have you included the information requested as detailed in our Minimum Standards Reporting Checklist?	Yes
**Availability of data and materials**	Yes

Powered by Editorial Manager® and ProduXion Manager® from Aries Systems Corporation

All datasets and code on which the conclusions of the paper rely must be either included in your submission or deposited in publicly available repositories (where available and ethically appropriate), referencing such data using a unique identifier in the references and in the "Availability of Data and Materials" section of your manuscript.<br><br>Have you have met the above requirement as detailed in our Minimum Standards Reporting Checklist?	

Powered by Editorial Manager® and ProduXion Manager® from Aries Systems Corporation

Manuscript
Click here to download Manuscript renamed_23fc8.docx

Screening methods for detection of ancient *Mycobacterium tuberculosis* complex fingerprints in NGS data derived from skeletal samples

Paulina Borówka[1], Łukasz Pułaski[2,3], Błażej Marciniak[4,5], Beata Borowska-Strugińska[1], Jarosław Dziadek[3], Elżbieta Żądzińska[1], Wiesław Lorkiewicz[1,#], Dominik Strapagiel[4,5,#]

1 Department of Anthropology, Faculty of Biology and Environmental Protection, University of Lodz, 90-237 Łódź, Poland;

2 Department of Molecular Biophysics, Faculty of Biology and Environmental Protection,University of Lodz, 90-237 Łódź, Poland;

3 Institute of Medical Biology, Polish Academy of Sciences, 93-232 Łódź, Poland;

4 Biobank Lab, Faculty of Biology and Environmental Protection, Department of Molecular Biophysics, University of Lodz, 90-231 Łódź, Poland;

5 BBMRI.pl Consortium, 54-066 Wrocław, Poland.

Corresponding author:

Dominik Strapagiel, Biobank Lab, Department of Molecular Biophysics, Faculty of Biology and Environmental Protection, University of Lodz, Pilarskiego 14, 90-231 Łódź, Poland, +48426655702, dominik.strapagiel@biol.uni.lodz.pl

Wiesław Lorkiewicz, Department of Anthropology, Faculty of Biology and Environmental Protection, University of Lodz, 90-237 Łódź, Poland, +48426354456, wieslaw.lorkiewicz@biol.uni.lodz.pl

Abstract

Background: Recent advances in ancient DNA (aDNA) studies, especially in increasing isolated DNA yields and quality, opened the possibility of analysis of ancient host microbiome. However, this analysis could lead to numerous pitfalls, including spurious identification of pathogens based on fragmentary data or environmental contamination, leading to incorrect epidemiological conclusions. Within the *Mycobacterium* genus, MTBC (*Mycobacterium tuberculosis complex*) members responsible for tuberculosis share up to ~99% genomic sequence identity, while other more distantly related MOTT (*Mycobacteria* other than *tuberculosis*) can be causative agents for pulmonary diseases or soil dwellers. Therefore, reliable determination of species complex is highly relevant for interpretation of sequencing results.

Results: Here we present a novel bioinformatical approach, used for screening of ancient tuberculosis in sequencing data, derived from 28 individuals (dated 4400 - 4000 BC and 3100 - 2900 BC) from Central Poland. We demonstrate that cost effective next generation screening sequencing data (c.a 20M reads per sample) could yield enough information to provide statistically supported identification of probable ancient disease cases.

Conclusions: Application of appropriate bioinformatic tools, including an unbiased selection of genomic alignment targets for species specificity, makes it possible to extract valid data from full-sample sequencing results (without subjective targeted enrichment procedures). This approach broadens the potential scope of paleoepidemiology both to older, suboptimally preserved samples and to pathogens with difficult intrageneric taxonomy.

Keywords

ancient DNA, aTB, ancient tuberculosis, NGS

Background

A rapid population growth initiated in Neolithic period, connected with domestication of animals and increase of human sedentism, played a key role in pathogen transmission within the so-called first epidemiological transition[1-4]. The identification of infectious diseases and selection of unique fingerprints of their causative agents, especially those derived from skeletal elements, are still of the greatest interest for paleopathologists and anthropologists, which is evidenced by the range of available analysis methods. Members of *Mycobacterium tuberculosis* complex (MTBC) are genetically very closely related and are causative agents for one of the oldest human infectious diseases – tuberculosis (TB). It is a disease that may leave lesions on patients' bones, enabling a diagnosis based on bone morphology [5]. The main problem of paleopathological diagnoses based solely on dry bones is that there are no pathognomonic skeletal indicators of TB. The most reliable skeletal indicator of TB are destructive lesions in thoracic and lumbar spine sections, which in advanced disease stage lead to destruction and collapse of vertebral bodies, resulting in spinal kyphosis, or gibbus, known as Pott's disease [5, 6]. However, many other conditions, like chronic pyogenic osteomyelitis, *Brucella* osteomyelitis, fungal infections, typhoid spine, vertebral fractures, septic, traumatic, and rheumatoid arthritis, malignant bone tumors can all affect the spine and produce similar pathological conditions which are difficult to distinguish from tuberculosis in paleopathological practice [7, 8]. Diagnoses based on other types of bone lesions are even more tentative; these are primarily based on osteomyelitis of the joints (most commonly the hip and knee, but also ankle and elbow) and periosteal reactive lesions (mainly in the ribs or diaphysis of the long bones, including tubular bones of the hands and feet in children [6, 8]. Lastly, morphological studies of bones do not permit detection of many individuals affected with TB in past human populations: data from the pre-antibiotic era show that bone changes occur only in about 3–7% of individuals with active TB [8].

Since the 1990s, new possibilities to diagnose TB in archaeological specimens have arisen, offered by the detection and analysis of mycobacterial DNA and other biomolecules specific to MTBC at the molecular level [9-20]. A common complication in molecular studies for ancient MTBC detection is the presence of DNA and other metabolites from the whole microbiome of the individual whose remains are being analysed as well as from environmental bacteria that have colonised the skeleton *post-mortem* [21, 22]. These contaminants might include Mycobacteria other than *M. tuberculosis* (MOTT), some of which are prevalent in the environment, while others are associated with clinical cases of non-tuberculosis diseases [21, 23-25]. It should be emphasized that members of *Mycobacterium tuberculosis* complex themselves are characterized by a particular high sequence similarity [26, 27], which leads to often unsurmountable difficulties in distinguishing them on the molecular level.

Detection of cell wall components such as mycolic, mycocerosic and mycolipenic acids [12, 14, 17, 18] with matrix-assisted laser desorption/ionization tandem time of flight (MALDI-TOF) which present profiles specific for MTBC is considered a reliable method to identify ancient causative agents in human archaeological samples. On the other hand, initial attempts to use mass spectrometry to detect cell wall lipids were shown to be erroneous in some cases [14, 28, 29]. Polymerase chain reaction, followed by gel electrophoresis, is still a popular method for detection of MTBC ancient DNA in human samples such as bones and teeth [30-32], mummified soft tissues [33, 34], or calcified pleura [9]. Known cases of tuberculosis disease proven on the basis of ancient DNA derived from human material are old as 9000 BC [35], through Iron Age [36] and up to modern times [37]. However, PCR-based methods have not been without controversy due to the possibility of cross-contamination as well as limitations of selection of proper sequences. While repetitive insertion sequences, e.g. IS6110 and IS1081, are widely used and sometimes considered as a biomarker specific to MTBC bacteria [32], the current consensus recommends strong caution in their use due to their presence in MOTT bacteria. Those commonly used markers have even been found to occur in soil mycobacteria [38-43], and even weak homology can cause false-positive PCR results for unrelated microbes [38, 44].

Recently, next generation sequencing (NGS) methods were introduced for detection of causative agents of ancient diseases [45, 46], including MTBC, with or without pre-enrichment of MTBC aDNA [47-50]. The increasing amount of data generated by NGS and efficiency of non-Sanger-based sequencing platforms requires a new approach in processing tools: suitable bioinformatic pipelines are required for reliable DNA analysis of ancient causative agents. Similar to PCR, where the use of only short conserved regions considered as specific for MTBC may lead to false positive results, improper analysis of NGS data can misinterpret sequences from modern known or unknown environmental *Mycobacteria* which are present in ancient human skeletons [25]. New analytical tools for more unequivocal answers to questions of identification and differentiation of *ante-mortem* causative from *post-mortem* non-causative microbial agents are urgently needed. Application of specifically designed *in silico* (bioinformatical approach) verification methods for improved downstream processing of molecular fingerprint data from ancient samples is necessary for drawing conclusions on clinical prevalence and epidemiology of pathogenic mycobacteria in history. Here we present an improved strategy for specific identification of bacteria from the *M. tuberculosis* complex in ancient non-enriched NGS data. Main purpose of this study was to design an unbiased genomic marker alignment query composed of sequences belonging strictly to MTBC members. Subsequently, appropriate bioinformatic alignment algorithms and statistical tools allow the identification of tuberculosis causative agents, using fragment length variation to balance selectivity (species specificity) with sensitivity of detection.

Sample Description

Ancient bone samples come from skeletal remains of 28 individuals representing two Neolithic populations from the Kujawy region in Central Poland: the Middle Neolithic Brześć Kujawski Group of the Lengyel culture (BKG), dated to ca. 4400-4000 BC (26 individuals) and the Late Neolithic Globular Amphora culture (GAC), dated to ca. 3100-2900 BC (2 individuals), previously described in [17, 51] (Supplementary Tab. 1). The skeletons come from two archaeological sites, BK 3 and BK 4, which represent relics of a settlement and cemetery of the BKG culture with some secondary objects within them, like the GAC grave. Both sites overlap each other, thus soil conditions and diagenetic agents were similar for all skeletal remains analyzed. Bone material was taken mainly from vertebral bodies of individuals with well-preserved skeletons. One of two individuals belonging to GAC reveals bone lesions which are consistent with Pott's disease. BKG samples provided more ambiguous evidences of skeletal lesions. One individual shows destructive lesions of the thoracic and lumbar vertebrae with central collapse of the vertebral bodies which may indicate tuberculous spondylodiscitis. Three other individuals of this population reveal only relatively mild and nonspecific inflammatory bone changes in the postcranial skeleton which were located on the internal surface of the ribs, tibia and femur shafts, as well as foot bones.

Analyses

Reference target construction (alignment target)

As our main reference sequence, we used the most commonly applied modern laboratory strain of *M. tuberculosis* (MTB), H37Rv, for which the whole genomic sequence is available. In order to select a subset of this reference sequence as an alignment target providing enhanced specificity for tuberculosis-causing agents (MTBC members), we first derived a set of all protein-coding sequences (CDS) from the H37Rv genome using the RAST tool [52]. These 4,360 sequences were screened using the BLAST tool (Megablast) at the National Library of Medicine sequentially against 12 available genomic sequences of selected MOTT: *M. kansasii*, *M. avium subsp. paratuberculosis, M. ulcerans, M. smegmatis, M. fortuitum, M. haemophilum, M. marinum, M. simiae, M. asiaticum, M. xenopi, M. phlei, M. abscessus*. Any detected similarities (gapless alignments >10 bp) between a H37Rv CDS and any MOTT genomic sequences resulted in the exclusion of this CDS from the result dataset, which was therefore restricted to sequences fully specific for MTBC, having no homologs in any MOTT genome. The resulting set of sequences was subsequently called the <u>Borówka et al. alignment target</u> and consisted of 1,534 coding sequences with total sequence length of 0.814 Mbp. Since no sequences from other MTBC species were used at this stage, and it is known that they exhibit up to 99.9% nucleotide

sequence similarity [53], the constructed alignment target cannot be considered specific only for *M. tuberculosis*, but rather for the whole MTBC; this is justified in epidemiological studies on ancient samples by the need to include all clinically equivalent causative agents for the same disease entity: tuberculosis. For comparison purposes, we prepared and used two literature-derived, knowledge-based H37Rv sequence subsets as alternative alignment targets: the c. 0.046 Mbp sequence used for capture enrichment in Bouwman et al. (2012) [50] for sequencing mycobacterial samples from a 19th century skeleton, subsequently called the <u>Bouwman et al. alignment target</u>, and the two genes (*katG* and *mpt40*, total length 0.004 Mbp) listed as MTBC-specific among the capture enrichment probes used by Bos et al. (2014) [48] for sequencing mycobacterial samples from 11th-13th century Peruvian skeletons, subsequently called the <u>Bos et al. alignment target</u>. All the reference sequences were prepared for alignment by indexing with the suffix array - induced sorting algorithm, implemented in the BWA software package (BWA).

Since the construction of the Borówka et al. alignment target was based on elimination of sequences similar to other mycobacterial species, we reasoned that the performance of an alignment target is directly linked to amount of similarities between the MTB genome and other potentially interfering mycobacterial species (both ancient and environmental) present in the ancient host-derived sample. In order to quantify this, we subjected the publicly available genome sequences of *Mycobacterium* species to an *in-silico* procedure to generate collections of short sequences broadly analogous to authentic NGS reads. Since including reads below a certain length threshold in similarity analysis of ancient microbial DNA leads to non-specific matches (for both evolutionary and statistical reasons); this threshold is usually arbitrarily assumed around 30 bp, but a broader analysis might make it easier to construct a reliable algorithm for detection of specific ancient pathogens. Therefore, in our further analysis both of reference and authentic ancient NGS sequences we extracted groups (bins) of non-human sequences over several length thresholds: ≥20bp, ≥25bp, ≥30bp and ≥35bp, to enable a thorough analysis of specificity gain upon increase in minimal sequence length. For reference *Mycbacterium* genomes, k-mers of specified length (corresponding to the lower limit of read length for NGS bins: 20, 25, 30 or 35) were filtered against the human genome assembly hg19, and the resulting "short read" collections were aligned to the full MTB reference genome or its selected subsets (Borówka et al., Bouwman et al. and Bos et al. alignment targets). Table 1 shows the respective number of genomic k-mers from MTB complex and MOTT species which match the MTBC alignment targets as well as the total lengths of assayed genomes for comparison. Since the various subsets of the MTB genome differ in length and thus the probability of random match increases with target length, we standardised the obtained data by presenting it as percentage of k-mers from a given mycobaterial genome that match the alignment target, divided by the ratio of target length to the full MTB genome length (genomic coverage of the target). These values, which are an inverse measure of alignment target specificity (they increase if more "reads" from a species which is not MTB or MTBC can be mistaken for MTBC), are shown in Table 1. As a reference, the MTB genome itself was also subjected to this procedure - obviously, the match percentage values are almost 100% here. Several conclusions can be drawn from these data: firstly, it is obvious that selecting longer reads (in this case longer k-mers) for comparison increases specificity, with reads 30 bp long or longer optimal for specific identification of the MTB complex, reflecting a common consensus in the field. However, it is important to note that shorter reads still add important information to the analysis, as the rate of specificity increase (decrease in matching read percentage with increase in read length) varies between species (i.e. some species have longer stretches of highly similar sequence to MTB). For example, while *M. smegmatis* has a very high match percentage to the Borowka et al. alignment target at low read length, this is rapidly lost at longer (more genuine) read lengths; the opposite is true e.g. for *M. marinum*. It is a derivation of the evolutionary history of the genus, but in this case also a practical caveat for further interpretation of sequence matches in actual aDNA samples. Moreover, the specificity of various alignment targets varies, with the Borówka et al. target being consistently the most specific (for longer k-mers) for distinguishing MOTT, while it is (by design) not well suited to distinguishing other members of the MTB complex from MTB itself.

Since we intended to develop a highly specific screening test (based on low depth sequencing strategy) for verification of MTBC infection in Neolithic samples with *a priori* relatively low degree of aDNA preservation, we decided on a statistical approach. Since any

preserved ancient mycobacterial DNA would be only a fraction of total aDNA, and it in turn would only be a fraction of total reads (the balance being modern environmental metagenome), a balance between sensitivity and specificity in verifying this very low number of reads must be struck. In sedentary, communal populations MTBC infection tends to be epidemic in character, but in most individuals with latent infection the microbial load (and thus the probability of DNA survival in ancient samples) is relatively low and constant. Any similarity analysis based on sequence alignment will also invariably generate false positive alignment hits, thus, it would be impossible to construct a test with sufficient statistical power to distinguish individuals genuinely free of ancient MTBC and those with average/modest latent infection. Therefore, we concentrated on the detection of outlier individuals with high microbial load (which may be later selected for enrichment-based further genetic analysis, such as phylogenetic studies or genome reconstruction), measured by the positive read ratio (the intrinsically very low ratio of reads matching the MTBC alignment target to all eligible reads). Based on the epidemiology of MTBC infection, we assumed a quasi-normal distribution of positive read ratios in a randomly selected sample of ancient individuals, with outliers as candidates for active tuberculosis and for selection for more in-depth studies. Thus, our method was based on standardising read ratio values to normal distribution parameters (arithmetic mean and standard deviation) and, as a further step in the detection algorithm for aTb, we applied a typical cutoff value of 1.5xSD to detect outliers.

As a first stage of testing our screening approach on actual NGS data from ancient material, we used a control dataset based on published NGS results of confirmed tuberculosis-infected individuals - 18th/19th-century mummified bodies from a crypt in Vác, Hungary, described by Kay et al. (2015) [46]. The aim of Kay et al. was to reconstruct and analyse historical genome sequences of *M. tuberculosis*, which resulted in sequencing results with high coverage. Since all these samples (26 bodies) were previously demonstrated by PCR to come from infected individuals [54], application of our screening procedure did not aim at distinguishing "positive" from "negative" samples, but at validating the selection of individuals with highest microbial load (especially since some of them were sampled from 1-3 different parts of the body), at the same time enhancing specificity (vs. MOTT). We used the Kay et al. dataset for verification of specificity of all applied alignment targets: Borówka et al., Bouwman et al., Bos et al. and the whole genome sequence of *M. tuberculosis* H37Rv, with our algorithm aimed at detection of strongest aTb outliers. While application of the Borówka et al. target sequence (with 30 bp read length cutoff) detected four samples as outliers, they turned out to belong only to two individuals (bodies 68 and 92) (Supplementary Tab. 2). This validated our approach as a suitable method for selecting ancient samples with highest MTBC genetic material content, especially since, despite our alignment target consisting only of sequences specific exclusively for MTBC, it turned out that those four samples were also those that showed the highest ratio of aligned reads to the full *M. tuberculosis* reference sequence (and thus the highest number of reads used to reconstruct the ancient genome) in the original study by Kay et al. (shown there in Supplementary Tab. 2). Moreover, only the two alignment targets prepared with both specificity and sensitivity in mind (Borówka et al. and Bouwman et al.) led to identification of all three samples from body 68 as outliers.

Subsequently, we applied the full statistical approach (with all four NGS read length bins) and the four selected genomic alignment targets: full reference *Mycobacterium tuberculosis* H37Rv genome (broadest possible target), two published targets consisting of rationally selected genes (applied previously to enrichment-based sequencing: Bouwman et al. and Bos et al.) as well as the novel specificity-tailored target (Borówka et al.), to the Neolithic samples from Brześć Kujawski. Table 2 presents the number of reads in each read length bin used for alignment with targets and statistical analysis, while Supplementary Tables 3-6 show the alignment results as numbers and ratios of matching reads. Fig. 1 presents the results of statistical analysis as outlying standardised ratio values in different read length bins. Overall, the expected population structure of majority of individuals with few positive reads and outlier individuals with an exceptional number of positive reads is confirmed. However, it is immediately obvious that the composition of outlier individuals depends strongly not only on the genomic alignment target, but also on minimum length of reads used for the alignment. There are individuals who remain positive (with a high relative ratio of reads aligning to the respective target) for all four length bins (e.g. 4_BK4 for the *Mycobacterium tuberculosis* H37Rv target), i.e. the share of putative MTBC-derived sequences remains constant despite the decrease in number of analysed sequences and increase in sequence

complexity. There are individuals who, despite being outliers for the bins including shorter reads, lose this status for the more restrictive bins (e.g. 55_BK4 for the Borówka et al. target), i.e. the majority of their MTBC-like sequences were of low complexity. Contrastingly, in some individuals the share of MTBC-like sequences increases above the cut off value only for bins with longer reads (e.g. 31_BK4 for the Borówka et al. target), i.e. most specifically aligned fragments are relatively long. It is again apparent that since most of this change concerns reads between 20 and 29 bp in length, the optimal threshold for read aligning to a genomic target for specificity towards MTBC is ≥30 bp. Thus, the three individuals which exceed the threshold of 1.5xSD for the MTBC-specific Borówka et al. target (17_BK4, 29_BK4 and 31_BK4) are considered with high probability to be ancient cases of MTBC infection and merit selection for further in-depth studies by a more cost-intensive approach.

Since the cut off-based detection algorithm, while robust for the presented dataset, may be less suitable for other, less homogenous groups of ancient individuals, we also set out to construct an objective, parametric testing-based outlier detection algorithm. Since the main objective of our overall study is specificity of MTBC detection, we applied this algorithm to the original Borówka et al. genomic alignment target. Based on the observation that positive read ratio tends to depend monotonically on read length bin – either consistently increasing or decreasing for outlier individuals – we decided to calculate a monotonicity parameter. We first standardised positive read ratios as percentage of average positive read ratio (without assumptions towards normal distribution, Supplementary Fig. 1) and then calculated ratios of these values for adjoining read length bins (≥25bp/≥20bp, ≥30bp/≥25bp and ≥35bp/≥30bp). The arithmetic mean of these values (Supplementary Tab. 7) depended on monotonicity of the studied relationship and had a normal distribution among individuals in our study. For outlier detection, we applied a one-tailed critical z value test on both tails on the sample. We consider the positive outliers (individuals with consistently increasing share of positively aligned reads with increasing read length) to be potential individuals with high MTBC loads, suitable for further analysis both by virtue of good mycobacterial genomic material preservation and high certainty of this material belonging to ancient MTBC. On the other hand, negative outliers may either be individuals with ancient MOTT infection (we suggest this as highly probable for 4_BK4) or samples with high proportion of short, non-specific alignments, probably due to environmental contamination (most probably 55_BK4) - to distinguish these two groups, a comparison with the more Mycobacterium-generic whole-genome alignment target is necessary (see below). This approach, while retaining the strong specificity of the cut off approach, gains increased sensitivity due to inclusion of individuals with high background of environmental sequences (low initial positive alignments in the short-read bin) which nevertheless retain specific long positively aligned sequences upon read length restriction, e.g. 21_BK4.

One of immediately obvious results of our analysis was that the comparison of alignment targets constructed with different assumptions leads to surprisingly large differences in assignation of individuals. Aligning aDNA sequences versus the whole MTB genome results in identification of two strong outliers (4_BK4 and 32_BK4). The same two individuals are identified, albeit with a smaller divergence, by using the enrichment bait sequence set uses by Bouwman et al. as alignment target. Since this subset of genomic sequences was originally selected for enrichment of lineage-distinguishing polymorphisms rather than for MTB complex specificity, this result is expected and confirms the efficiency of the outlier detection method and ≥30bp as optimal read length. On the other hand, our Borówka et al. genomic subset selected on the basis of MTB complex specificity led to identification of three different individuals as outliers (17_BK4, 29_BK4 and 31_BK4), while 4_BK4 and 32_BK4 had positive read values close to average. This is even more conspicuous when positive ratio values for the two different alignment targets (whole genome and specific subset Borówka et al.) are plotted against each other (Fig. 2). In our opinion this points to the broadly recognized risk of mistakenly identifying ancient infections caused by MOTT as tuberculosis based on the extensive similarity between the respective mycobacterial genomes. While restricting the alignment target leads to loss of sensitivity due to unavoidable decrease of absolute number of aligned reads, which is a significant problem for ancient DNA, it is offset by the increase in specificity of detection. This distinction is crucial for epidemiological hypotheses where elimination of false positives is of paramount importance. We further show this by aligning our reads to the purportedly MTBC-specific target sequences selected by Bos et al. (sequences of only two *M. tuberculosis* specific genes), where increase of specificity leads to detection of the 29_BK4 individual, but the extreme loss of sensitivity linked to minuscule absolute number of

reads (the highest number of positive reads in the ≥30bp bin is 13 – see Supplementary Tab. 6) leads to high experimental noise and low reliability of assignment of individuals, and it is not recommended.

Since for two individuals which were strongly enriched in mycobacterial sequences (4_BK4 and 32_BK4) we posit the existence of an ancient MOTT infection (as they do not score highly in comparison with the specific Borówka et al. alignment target), we decided to verify if this assumption is supported by aligning the optimal read bin (≥30bp) to full genomes of other mycobacterial species as targets. Indeed, as seen in Supplementary Fig. 2, those two individuals are also strong outliers in read ratio values after aligning to the *M. marinum* genome - moreover, when plotted against read ratio values for the MTB genome, it is apparent that they show higher similarity to *M. marinum*, since they are located on the *M. marinum* side of the read ratio regression line. This finding validates our workflow in that it corroborates the usefulness of read length binning while further demonstrating the advantages of read aligning to targets selected for species discrimination (like the Borówka et al. target) which allow for immediate flagging of suspicious samples with spuriously high absolute similarity to the MTB genome. We have also attempted to verify the possibility of distinguishing samples with predominantly ancient mycobacterial sequences from samples with recent environmental MOTT contamination by performing mapDamage analysis. MapDamage analysis shows that the low absolute number of reads that map to all alignment targets (including the full MTB genome) in the case of our samples prevents us from drawing meaningful conclusions in this regard (even for the samples with highest read numbers - 4_BK4, 32_BK4, 17_BK4, 29_BK4, 31_BK4). For confirmation of ancient status of analysed reads Mapdamage analysis were also performed and is presented in Supplementary Fig. 3 for individuals with potential MOTT and MTBC infections.

Discussion

Evolutionary and ecological complexity of mycobacteria, including the existence of a group of closely related pathogens known as *Mycobacterium tuberculosis* complex, a large number of more distantly related human and animal pathogens causing diseases other than tuberculosis, and an abundance of free-living (including soil- and water-borne) mycobacterial species in the environment all contribute to difficulty in unequivocal determination of ancient tuberculosis on the basis of MTBC aDNA. Present-day paleoepidemiology uses tools of classical biological anthropology as well as modern clinical diagnostics at the molecular level. Morphological diagnosis of tuberculosis is based on certain bone changes, especially those described as Pott's disease. This approach is not optimal from the point of view of sensitivity, since bone lesions are present only in 2% of all cases of tuberculosis infection and 10-20% of cases of extrapulmonary tuberculosis [39, 55]. Specificity of this tool is also relatively low - bone lesions that mimic Pott's disease occurs in many other unrelated conditions. In spite of that limitations, osteological analysis is often the main starting point of a study and cannot be disregarded. However, in our study the occurrence of bone lesions that could be linked in any way with tuberculosis did not correlate with the results of our genetic analyses. There are two possible explanations for this fact. First, the bone changes were not caused by tuberculosis, which is in accordance with a lack of pathognomonic characteristics of the disease on the skeleton alone, as was clarified before; it applies primarily to the graves 12_BK4, 18_BK4, 47_BK4, and 73_BK4. It may also be that the preservation of MTBC aDNA was too poor to pass the sensitivity/specificity threshold of the method proposed here.

Among molecular techniques which are used for diagnosis of ancient tuberculosis cases, both biochemical methods based on mass spectrometry and PCR amplification of marker sequences have been successfully used in literature, e.g. for preliminary description of the Hungarian mummies used subsequently to reconstruct aTB genomes [46, 54]. However, both these groups of methods suffer from a number of drawbacks which make them less useful in an ancient epidemiological context than in a contemporary one: environmental contamination from modern soil mycobacteria can overwhelm both traces of ancient MTBC mycolic acids and less specific PCR amplicons, while strong care must be taken to prevent in-lab cross-contamination with genuine MTBC samples. Therefore, NGS has a number of advantages in diagnosis of ancient tuberculosis, having the potential to be both highly sensitive and highly specific; but the balance between sensitivity and specificity depends on

the selection of reference genomic sequences and crucially on the method of alignment. Large amount of generated data allows potentially to detect ancient mycobacteria selectively, unequivocally and semi-quantitatively, while making possible additional analyses such as preservation period-related DNA damage pattern detection (e.g. mapDamage [56, 57], phylogenetic analysis of genetic kinship [48] or even full genome reconstruction [46]. Due to small absolute amounts of actual ancient pathogen DNA in most types of human body samples, a common approach is to use pre-sequencing enrichment (usually using probe capture, e.g. [48]). Only in bodies preserved in exceptional, isolated conditions, such as the Hungarian mummies from a 18th century crypt, was a non-enriched metagenomics approach used [46]. Use of enrichment techniques strongly increases sensitivity, but comes with its own drawbacks (apart from increased cost), the most relevant of which is the need to pre-design a set of sequences (probes or primers) that will define and limit the scope of subsequently obtained NGS data. A full metagenome approach is often more relevant when dealing with a highly ancient sample like in the present study, when neither the infection prevalence nor the pathogen identity are known to any precision and a preliminary NGS study is needed for formulation of specific hypotheses and pre-selection of individuals for further analysis.

However, in the case of ancient MTBC (especially samples as old as our material is), specificity is a more important consideration than sensitivity – in this case not so strongly with regard to modern MTBC contamination in the laboratory (which would not mask ancient data in a semi-quantitative study and would be obvious if DNA damage analysis were performed), but mainly with regard to ancient MOTT which can be unpredictably genetically similar to MTBC. The sources of these MOTT can be either soil contamination (including dead animals) which could have happened at any time since inhumation (preventing reliable elimination by DNA damage analysis), or actual ancient MOTT which were pathogenic/infectious/commensal to ancient humans. Thus, the design of sequencing analysis workflow has to take into account the necessity to filter out unknown related sequences that are not derived from MTBC - this was the main rationale behind the design of our study. While contamination with mycobacterial sequences within the laboratory (amplicons, genuine Mycobacterium DNA) can be prevented by correct workflow (separation of pre- and post-PCR areas etc.), equipment and strict procedures, contamination by environmental DNA is inescapable and has to be taken into account in the case of archaeological bone samples preserved by inhumation. Since for ancient samples direct contact of bones with the environment has lasted for a very long time (unlike more recent samples from vault inhumation), mycobacterial DNA derived from environmental (soil) MOTT can have undergone accretion in bones throughout this period, with some of it ancient enough to be indistinguishable in terms of location and state of preservation from DNA of infectious microbes buried with the body. All MTBC are obligate pathogens and thus are an unlikely source of environmental contamination of ancient samples. Therefore, for preliminary identification of potentially interesting samples in ancient inhumated bones, specificity in methods of detection of ancient infectious agents from this group should be developed towards exclusion of MOTT, with distinction between members of MTBC as a secondary, much less important goal. Since MTBC also share a very high proportion of coding sequences, achieving specificity for *M. tuberculosis* s.s. could occur only by drastically limiting the size of the reference marker sequence, thus leading to very low sensitivity, especially for usually highly degraded aDNA. Moreover, the division of MTBC into lineages is not entirely concordant with classical taxonomic division into species, so attempting an artificial distinction between some lineage groups based on accumulated NGS data would not be recommended. Our approach is designed as a relatively low-cost, first-pass classification of ancient samples based on whole-metagenome NGS data. When a highly specific method like the one we propose is used to identify likely ancient MTBC infection, potential lineage determination or any other phylogenetic studies (in pre-selected samples) should proceed by other methods developed specifically for this purpose, based on the presence of lineage-specific polymorphisms (with the caveat that enrichment for specificity-related sequences before NGS will certainly lead to loss of the majority of phylogenetically important loci, so a full metagenomic sequencing round with sufficient coverage is inevitable).

We postulate that a combination of read length-based genomic alignment analysis and a careful knowledge-based selection of the alignment target makes it possible to achieve relatively high specificity of aTB detection against all potential false positive sources. Therefore, a robust tool for specifically identifying NGS-derived sequences that belong to ancient MTBC with high confidence is a priority task in molecular

paleoanthropology. Even more relevant to paleoanthropological studies, confusion between MOTT and MTBC can lead to spurious identification of ancient individuals as tuberculosis sufferers or carriers, invalidating conclusions relevant to paleoepidemiology. We demonstrate that read length selection is not only highly relevant (as has been shown before and by us, only reads above ca. 30 bp can be used with high confidence), but when a statistics-based approach to multiple length thresholds is used, it can yield a substantial increase in specificity of MTBC identification. At the same time, selection of pre-filtered alignment target, with combined knowledge-based (selection of transcribed sequences) and automated (exclusion of sequences aligning with MOTT genomes) delineation of MTBC-specific sequences (which we call the Borówka et al. target), makes it possible to perform in-depth specificity analysis by comparing the alignments of *in silico* fragmented mycobacterial genomes (mimicking actual NGS data). Combining the novel alignment target and the read length binning approach, we were able to select with high confidence three ancient individuals with probable ancient MTBC infection and two further individuals with highly probably ancient mycobacteriosis caused by MOTT (which would be misidentified as tuberculosis if another alignment target or to short reads were taken into account). Of course the limitations of our data make these identifications preliminary and another round of directed (e.g. enrichment-based) sequencing would be required both for positive identification of the infectious agent and for potential phylogenetical analysis of its spatial and/or temporal kinship. However, in our case read length analysis allowed us to suggest *M. marinum* as the potential ancient infectious agent based on statistical analysis; obviously, positive confirmation of this diagnosis would require tools that are currently unavailable such as proven *M. marinum*-specific enrichment probes as well as a much better sequence coverage that could be achieved in a preliminary study (Supplementary Fig. 3). Still, this possible pathogen identification is not at odds with the archeological context as the inhumation site is next to a lake (Smetowo) and within a geographical region rich in post-glacial lakes (Kujawy), so some individuals could have had routine professional contact with fish. Our combined procedures are a robust specificity-boosting tools, but obviously cannot be treated as ultimate proof (neither for disproval or confirmation of tuberculosis infection). Our samples are relatively old (in comparison to most other ancient tuberculosis cases studied by molecular means before) and thus the absolute read numbers from an unbiased NGS approach is low. We demonstrate that this disadvantage makes it e.g. difficult to perform DNA damage analysis. However, we provide a consistent proof of concept for a tool which would allow relatively cheap and unbiased selection of samples (e.g. individuals) for further analysis, e.g. by enrichment capture NGS. Thus, we suggest that it is possible to use global NGS results from ancient samples as an economical pre-screening tool for more complex methods, while applying bioinformatic tools to maximise the number of reliable conclusions that can be drawn from a limited dataset.

Methods

Ancient DNA extractions

The procedures of sample preparation were conducted in sterile and dedicated ancient DNA sample preparation facility at University of Lodz, with all standard precautions taken to avoid sample contamination. All disposable materials, buffers, water, clean room surfaces and bone material, were UV-irritated for min. 30 minutes before any proceeding steps. The fragments of bone material were isolated using Dremel disks, (USA), surface-cleaned, UV-irradiated for 7.5 minutes on each side, and ground into a fine powder, further used for DNA extraction procedures following the protocol of Dabney et al. with modifications [58-60]. Ancient DNA was successfully isolated from all bone samples (See Supplementary Fig. 3). Illumina libraries were prepared in separate facility, according to Meyer et al. protocol [61] without UDG treatment of the samples. All libraries were subjected to the screening next-generation sequencing on the Illumina Nextseq 500 platform (100bp single-end sequencing), yielding between 2.2 and 33.9 million reads per individual (median number of reads after incomplete and truncated read trimming – 16.9 million reads per individual, Tab. 2). This dataset contains ancient human sequences from the deceased individuals, ancient microbial

sequences from parasites, pathogens, commensals or symbionts of the deceased individuals, as well as genomic sequences from environmental organisms (mainly microbes, but also potentially higher Eukaryotes), to which the skeletal remains were exposed *post-mortem*.

Bioinformatical procedures

Raw NGS reads were subjected to standard quality processing such as trimming and adapter sequence removal (-q 30 --phred33 --illumina --length 20), using the Trim Galore! software package [62]. Since the predominant expected type of sequence in skeletal samples is ancient human genomic DNA and its presence would unnecessarily complicate our analysis, the read datasets were subsequently subjected to filtering by alignment to the standard (hg19) human genome reference sequence. This alignment was performed using the BWA_aln algorithm (-n 0.04, -l 1000), with duplicate removal, using the AGAT software tool - ocwrapper3mt.py script [63]. Any read which aligned without gaps within the default mismatch rate (dependent on sequence length, e.g. 2 mismatches per 17 bp) was eliminated from the sample dataset. Subsequently, separate sub-datasets (bins) of reads were generated on the basis of (trimmed) read length: minimal read length threshold ≥20bp, ≥25bp, ≥30bp and ≥35bp. These datasets were used for alignment to reference targets. These procedures were applied also to the Kay et al dataset, used for the Borówka et al. method verification.

Estimation of terminal base deamination damage pattern was done by using mapDamage2.0 analysis with specifying a length (-l) of 75 bp and library build preparation type (--single-stranded) (Supplementary Fig.3).

Query sequence preparation

Selected 18 reference *Mycobacterial* genomes, including 5 of *M. tuberculosis* complex (underlined): *M. abscessus, <u>M. africanum</u>, M. asiaticum, M. avium, <u>M. bovis</u>, <u>M. caprae</u>, M. fortuitum, M. haemophilum, M. kansasii, M. leprae, M. marinum, <u>M. microti</u>, M. phlei, M. simiae, M. smegmati, <u>M. tuberculosis</u>, M. ulcerans, M. xenopi* were used. Nucleotide sequences of each organism have been subjected to fragmentation with FA_TOOL script (small_tool.py) [64] respectively for 20 bp, 25 bp, 30 bp and 35 bp-long fragments and allocated in same manner to length bins. Further, fragmented genomes were used for specificity testing of each constructed target which allowed to overcome the problem of very short and non-specific fragments with threshold estimation.

Verification of specificity and sensitivity of NGS screening method

Due to the lack of available NGS data of positive M. tuberculosis cases, we tested in-silico methods by the using Kay et al. (2015) dataset (PRJEB7454), derived from Hungarian mummies tissue microbiome sequencing. SRA files for each sample were identified and downloaded, further fastq files passed through trimming with deprivation of the adapter sequences [65]. Raw sequencing files were conducted to human genome reference sequence (hg19) filtration in spite the fact that host DNA material could be dominant in the sample. Alignment was performed to the tested targets M. tuberculosis H37Rv, Borówka et al., Bos et al., and Bouwman et al. using the AGAT software tool [63]. Statistics for each individual are presented in Supplementary Table 2. Summarized results of aTB cases from Brześć Kujawski are included in Supplementary Tables 3-6.

Statistical processing and parametric testing-based outlier detection algorithm

Collected unmapped sequences from original dataset, as well as from Kay et al dataset, were aligned to constructed marker sequences: *M. tuberculosis H37Rv*, Borówka et al. **(**Supplementary Table 9**)**, Bos et al., and Bouwman et al. with application of experimentally determined minimal read length threshold ≥17 bp, ≥20bp, ≥25bp, ≥30bp and ≥35bp for detection of potential ancient MTBC cases. For detection of outlier individuals with high microbial load/positive read ratio, we standardised read ratio values to normal distribution parameters (arithmetic mean

and standard deviation) and, as a further step in the aTb detection algorithm, applied a typical cut off value of 1.5xSD to detect outliers, postulating these to be candidates for active tuberculosis.

Based on the observation that positive read ratio tends to depend monotonically on read length bin – either consistently increasing or decreasing for outlier individuals – we decided to calculate a monotonicity parameter. We first standardised positive read ratios as percentage of average positive read ratio and then calculated ratios of these values for adjoining read length bins (≥25bp/≥20bp, ≥30bp/≥25bp and ≥35bp/≥30bp). For outlier detection, we applied a one-tailed critical z value test on both tails of the sample. We consider the positive outliers (individuals with consistently increasing share of positively aligned reads with increasing read length) to be confirmed ancient tuberculosis sufferers (See Supplementary tables 2-4).

Availability of supporting data and materials

The datasets supporting the conclusions of this article are available under the NCBI repository project "Identification of ancient tuberculosis in human archaeological remains" (acc. num. PRJNA422903) including Biosamples and related Sequence Read Archive (SRA).

Additional files

Borówka_et_al_Supplemetary_Tables.xls

Borówka_et_al_Supplementary_Tables_legends.doc

Borówka_et_al_Supplementary_Figures.pdf

Declarations

Abbreviations

aDNA – Ancient DNA

aTB – Ancient tuberculosis

NGS – Next Generation Sequencing

MTBC – *Mycobacterium Tuberculosis* Complex

MOTT – Mycobacteria other than tuberculosis

SRA - Sequence Read Archive

Ethics approval and consent to participate

Not applicable.

Consent for publication

Not applicable

Competing interests

The authors declare that they have no competing interests.

Acknowledgments

We would like to thank Anna Brzostek for additional laboratory work.

Funding

The study was financed by the Polish Ministry of Science and Higher Education grant DIR/WK/2017/01 and by the Polish POPC Grant 02.03.01-00-0012/17-00 from the European Regional Development Fund.

Author's contributions

P.B. and D.S. conceived the study, were responsible for extraction of aDNA, preparation of NGS libraries and Next Generation Sequencing of samples. P.B, D.S and Ł.P analyzed the data, discussed the results, and wrote the manuscript. Ł.P. participated in the statistical analysis and figure preparation. B.M wrote and ran AGAT primary analysis. B.B-S. precipitated in sample selection and preparation for laboratory phase. J.D., WL analyzed the samples for pathological changes, participated in the study design, analyzed and discussed the data, and participated in drafting the manuscript. E.Ż. participated in the study design, analyzed and discussed the data, and participated in drafting the manuscript. D.S. coordinated studies and was responsible for the final version of the manuscript; all authors read and approved the final manuscript.

References

1. Barrett, R., et al., *Emerging and re-emerging infectious diseases: the third epidemiologic transition.* Annual review of anthropology, 1998. **27**(1): p. 247-271.
2. Armelagos, G.J. and M.N. Cohen, *Paleopathology at the Origins of Agriculture*. 1984: Academic Press Orlando (FL).
3. Armelagos, G.J., A.H. Goodman, and K.H. Jacobs, *The origins of agriculture: Population growth during a period of declining health.* Population & Environment, 1991. **13**(1): p. 9-22.
4. Weiss, R.A. and A.J. McMichael, *Social and environmental risk factors in the emergence of infectious diseases.* Nature medicine, 2004. **10**(12s): p. S70.
5. Ortner, D.J. and W. Putschar, *Identification of Pathological Conditions in Human Skeletal Remains.* Smithsonian Contributions to Anthropology, 1985. **28**.
6. Aufderheide, A.C., Rodriguez-Martin, Conrado and O. Langsjoen, *The Cambridge encyclopedia of human paleopathology*. Vol. 478. 1998: Cambridge University Press Cambridge.
7. Holloway, K.L., et al., *Skeletal lesions in human tuberculosis may sometimes heal: an aid to palaeopathological diagnoses.* PLoS One, 2013. **8**(4): p. e62798.
8. Steinbock, R.T., *Paleopathological diagnosis and interpretation: bone diseases in ancient human populations*. 1976: Charles C Thomas Pub Limited.
9. Donoghue, H., et al., *Mycobacterium tuberculosis complex DNA in calcified pleura from remains 1400 years old.* Letters in Applied Microbiology, 1998. **27**(5): p. 265-269.
10. Donoghue, H.D., *Palaeomicrobiology of tuberculosis*, in *Paleomicrobiology*. 2008, Springer. p. 75-97.
11. Donoghue, H.D., *Human tuberculosisis-an ancient disease, as elucidated by ancient microbial biomolecules.* Microbes and infection, 2009. **11**(14): p. 1156-1162.
12. Redman, J.E., et al., *Mycocerosic acid biomarkers for the diagnosis of tuberculosis in the Coimbra Skeletal Collection.* Tuberculosis (Edinb), 2009. **89**(4): p. 267-77.
13. Mark, L., et al., *High-throughput mass spectrometric analysis of 1400-year-old mycolic acids as biomarkers for ancient tuberculosis infection.* Journal of Archaeological Science, 2010. **37**(2): p. 302-305.
14. Minnikin, D.E., et al., *The interplay of DNA and lipid biomarkers in the detection of tuberculosis and leprosy in mummies and other skeletal remains*. 2011, Verlag Dr. Friedrich Pfeil.
15. Tran, T., et al., *Beyond ancient microbial DNA: nonnucleotidic biomolecules for paleomicrobiology.* Biotechniques, 2011. **50**(6): p. 370-380.
16. Masson, M., et al., *Osteological and biomolecular evidence of a 7000-year-old case of hypertrophic pulmonary osteopathy secondary to tuberculosis from neolithic hungary.* PLoS One, 2013. **8**(10): p. e78252.
17. Borowska-Strugińska, B., et al., *Mycolic acids as markers of osseous tuberculosis in the Neolithic skeleton from Kujawy region (central Poland).* AnthropologicAl review, 2014. **77**(2): p. 137-149.
18. Gernaey, A.M., et al., *Mycolic acids and ancient DNA confirm an osteological diagnosis of tuberculosis.* Tuberculosis (Edinb), 2001. **81**(4): p. 259-65.
19. Boros-Major, A., et al., *New perspectives in biomolecular paleopathology of ancient tuberculosis: a proteomic approach.* Journal of Archaeological Science, 2011. **38**(1): p. 197-201.
20. Harkins, K.M., et al., *Screening ancient tuberculosis with qPCR: challenges and opportunities.* Philos Trans R Soc Lond B Biol Sci, 2015. **370**(1660): p. 20130622.
21. Campana, M.G., et al., *False positives complicate ancient pathogen identifications using high-throughput shotgun sequencing.* BMC research notes, 2014. **7**(1): p. 111.
22. Andam, C.P., et al., *Microbial Genomics of Ancient Plagues and Outbreaks.* Trends Microbiol, 2016. **24**(12): p. 978-990.
23. Bouwman, A.S. and T.A. Brown, *The limits of biomolecular palaeopathology: ancient DNA cannot be used to study venereal syphilis.* Journal of Archaeological Science, 2005. **32**(5): p. 703-713.
24. Tsangaras, K. and A.D. Greenwood, *Museums and disease: using tissue archive and museum samples to study pathogens.* Ann Anat, 2012. **194**(1): p. 58-73.
25. Müller, R., C.A. Roberts, and T.A. Brown, *Complications in the study of ancient tuberculosis: Presence of environmental bacteria in human archaeological remains.* Journal of Archaeological Science, 2016. **68**: p. 5-11.
26. Frothingham, R., H.G. Hills, and K.H. Wilson, *Extensive DNA sequence conservation throughout the Mycobacterium tuberculosis*

complex. Journal of clinical microbiology, 1994. **32**(7): p. 1639-1643.
27. Brites, D. and S. Gagneux, *Co-evolution of Mycobacterium tuberculosis and Homo sapiens.* Immunol Rev, 2015. **264**(1): p. 6-24.
28. Minnikin, D.E., et al., *Molecular biomarkers for ancient tuberculosis*, in *Understanding tuberculosis-deciphering the secret life of the bacilli*. 2012, InTech.
29. Minnikin, D.E., et al., *Essentials in the use of mycolic acid biomarkers for tuberculosis detection: response to â€šHigh-throughput mass spectrometric analysis of 1400-year-old mycolic acids as biomarkers for ancient tuberculosis infectionâ€ť by.* Journal of Archaeological Science, 2010. **37**(10): p. 2407-2412.
30. Spigelman, M. and E. Lemma, *The use of the polymerase chain reaction (PCR) to detect Mycobacterium tuberculosis in ancient skeletons.* International Journal of Osteoarchaeology, 1993. **3**(2): p. 137-143.
31. Donoghue, H.D., et al., *Ancient DNA analysis - An established technique in charting the evolution of tuberculosis and leprosy.* Tuberculosis (Edinb), 2015. **95 Suppl 1**: p. S140-4.
32. Müller, R., C.A. Roberts, and T.A. Brown, *Complications in the study of ancient tuberculosis: non-specificity of IS6110 PCRs.* STAR: Science & Technology of Archaeological Research, 2015. **1**(1): p. 1-8.
33. Pääbo, S., *Molecular cloning of ancient Egyptian mummy DNA.* Nature, 1985. **314**(6012): p. 644-645.
34. Salo, W.L., et al., *Identification of Mycobacterium tuberculosis DNA in a pre-Columbian Peruvian mummy.* Proceedings of the National Academy of Sciences, 1994. **91**(6): p. 2091-2094.
35. Hershkovitz, I., et al., *Detection and molecular characterization of 9,000-year-old Mycobacterium tuberculosis from a Neolithic settlement in the Eastern Mediterranean.* PLoS One, 2008. **3**(10): p. e3426.
36. Mays, S. and G.M. Taylor, *A first prehistoric case of tuberculosis from Britain.* International Journal of Osteoarchaeology, 2003. **13**(4): p. 189-196.
37. Zink, A.R., W. Grabner, and A.G. Nerlich, *Molecular identification of human tuberculosis in recent and historic bone tissue samples: The role of molecular techniques for the study of historic tuberculosis.* Am J Phys Anthropol, 2005. **126**(1): p. 32-47.
38. Dziadek, J. and A. Sajduda, *Specificity of insertion sequence-based PCR assays for Mycobacterium tuberculosis complex.* The International Journal of Tuberculosis and Lung Disease, 2001. **5**(6): p. 569-574.
39. Teo, H.E. and W.C. Peh, *Skeletal tuberculosis in children.* Pediatric radiology, 2004. **34**(11): p. 853-860.
40. Kent, L., et al., *Demonstration of homology between IS6110 of Mycobacterium tuberculosis and DNAs of other Mycobacterium spp.?* Journal of clinical microbiology, 1995. **33**(9): p. 2290-2293.
41. McHugh, T., L. Newport, and S. Gillespie, *IS6110 homologs are present in multiple copies in mycobacteria other than tuberculosis-causing mycobacteria.* Journal of clinical microbiology, 1997. **35**(7): p. 1769-1771.
42. Picardeau, M., et al., *Genotypic characterization of five subspecies of Mycobacterium kansasii.* J Clin Microbiol, 1997. **35**(1): p. 25-32.
43. Picardeau, M., et al., *Mycobacterium xenopi IS1395, a novel insertion sequence expanding the IS256 family.* Microbiology, 1996. **142**(9): p. 2453-2461.
44. Savelkoul, P.H., et al., *Detection of Mycobacterium tuberculosis complex with real time PCR: comparison of different primer-probe sets based on the IS6110 element.* Journal of microbiological methods, 2006. **66**(1): p. 177-180.
45. Rasmussen, S., et al., *Early divergent strains of Yersinia pestis in Eurasia 5,000 years ago.* Cell, 2015. **163**(3): p. 571-82.
46. Kay, G.L., et al., *Eighteenth-century genomes show that mixed infections were common at time of peak tuberculosis in Europe.* Nature communications, 2015. **6**.
47. Chan, J.Z., et al., *Metagenomic analysis of tuberculosis in a mummy.* N Engl J Med, 2013. **369**(3): p. 289-90.
48. Bos, K.I., et al., *Pre-Columbian mycobacterial genomes reveal seals as a source of New World human tuberculosis.* Nature, 2014. **514**(7523): p. 494-7.
49. Bos, K.I., et al., *Parallel detection of ancient pathogens via array-based DNA capture.* Philos Trans R Soc Lond B Biol Sci, 2015. **370**(1660): p. 20130375.
50. Bouwman, A.S., et al., *Genotype of a historic strain of Mycobacterium tuberculosis.* Proceedings of the National Academy of Sciences, 2012. **109**(45): p. 18511-18516.
51. Lorkiewicz, W., et al., *Between the Baltic and Danubian worlds: the genetic affinities of a middle neolithic population from central Poland.* PLoS One, 2015. **10**(2): p. e0118316.
52. Aziz, R.K., et al., *The RAST Server: rapid annotations using subsystems technology.* BMC Genomics, 2008. **9**(1): p. 75.
53. Djelouadji, Z., D. Raoult, and M. Drancourt, *Palaeogenomics of Mycobacterium tuberculosis: epidemic bursts with a degrading genome.* Lancet Infect Dis, 2011. **11**(8): p. 641-50.
54. Fletcher, H.A., et al., *Widespread occurrence of Mycobacterium tuberculosis DNA from 18thâ€“19th century Hungarians.* American Journal of Physical Anthropology: The Official Publication of the American Association of Physical Anthropologists, 2003. **120**(2): p. 144-152.
55. Peto, H.M., et al., *Epidemiology of extrapulmonary tuberculosis in the United States, 1993-2006.* Clinical Infectious Diseases, 2009. **49**(9): p. 1350-1357.
56. Ginolhac, A., et al., *mapDamage: testing for damage patterns in ancient DNA sequences.* Bioinformatics, 2011. **27**(15): p. 2153-5.
57. Jonsson, H., et al., *mapDamage2.0: fast approximate Bayesian estimates of ancient DNA damage parameters.* Bioinformatics, 2013. **29**(13): p. 1682-4.
58. Gamba, C., et al., *Genome flux and stasis in a five millennium transect of European prehistory.* Nature communications, 2014. **5**: p. 5257.
59. Pinhasi, R., et al., *Optimal ancient DNA yields from the inner ear part of the human petrous bone.* PLoS One, 2015. **10**(6): p. e0129102.
60. Fernandes, D., et al., *A genomic Neolithic time transect of hunter-farmer admixture in central Poland.* Scientific reports, 2018. **8**(1): p. 14879.
61. Meyer, M. and M. Kircher, *Illumina sequencing library preparation for highly multiplexed target capture and sequencing.* Cold Spring Harbor Protocols, 2010. **2010**(6): p. pdb. prot5448.
62. Krueger, F., *Trim Galore!: A wrapper tool around Cutadapt and FastQC to consistently apply quality and adapter trimming to FastQ files*. 2015.
63. Marciniak, B., P. Borówka, and D. Strapagiel, *AGAT tool - Ancient Genomes Analysis Tool. https://github.com/BiobankLab/AGAT;* 2016.
64. Marciniak, B., P. Borówka, and D. Strapagiel, *FA_TOOL-simple command line tool for fasta file editing. https://github.com/BiobankLab/FA_TOOL;* 2016.

Tables and Figure legends

Table 1. Number of genomic k-mers from MTBC and MOTT members after initial hg19 clearing step matching selected targets, with k-mer length distinction (≥20bp, ≥25bp, ≥30bp, ≥35bp). with estimation of percentage of k-mers from a given mycobaterial genomes matching the *M. tuberculosis* genome for query length ≥30 and ≥35.

	**k-mer length**		**Query length ≥20**					**Query length ≥25**					**Query length ≥30**					**Query length ≥35**				
	**Alignment target**	**Genome length (bp)**	**% of sequences mapped to M. tuberculosis genome**	**Full genome**	**Borowka et al.**	**Bos et al.**	**Bouwman et al.**	**% of sequences mapped to M. tuberculosis genome**	**Full genome**	**Borowka et al.**	**Bos et al.**	**Bouwman et al.**	**% of sequences mapped to M. tuberculosis genome**	**Full genome**	**Borowka et al.**	**Bos et al.**	**Bouwman et al.**	**% of sequences mapped to M. tuberculosis genome**	**Full genome**	**Borowka et al.**	**Bos et al.**	**Bouwman et al.**
**Species group**																						
	M. leprae	3268203	3.19%	140922	19736	101	1683	4.88%	215257	4349	85	2240	2.61%	115138	1430	26	1201	1.45%	63860	543	6	715
**MOTT**	*M. abscessus*	5067172	5.26%	232228	46530	158	2915	2.87%	126769	2816	103	1890	1.39%	61160	283	46	1065	0.75%	33175	62	14	644
	M. smegmatis	6988209	11.19%	493570	107339	543	6917	5.68%	250537	7793	340	2919	2.88%	127219	1187	162	1610	1.64%	72286	262	65	944
	M. fortuitum	6254616	8.48%	374030	77785	291	5208	5.22%	230382	5940	131	2774	2.69%	118483	958	40	1534	1.53%	67463	236	16	916
	M. phlei	5349645	8.98%	396255	88582	391	5909	6.57%	289788	9912	157	3597	3.45%	152331	1665	97	1951	2.03%	89593	377	56	1176
	M. simiae	5938797	9.33%	411677	80142	339	5414	9.51%	419641	12734	197	4578	5.35%	235800	3904	93	2702	3.15%	139050	1450	33	1575
	M. asiaticum	5910436	9.00%	396854	76829	413	5597	10.69%	471493	19780	392	5022	0.00%	265638	5366	186	2806	3.54%	156188	1531	71	1706
	M. xenopi	4434836	7.14%	314850	60482	262	4336	8.17%	360395	11534	207	4105	0.00%	200395	3233	120	2126	2.62%	115687	1060	68	1235
	M. marinum	6660144	9.48%	418304	82499	466	5715	14.08%	621166	52438	707	6366	7.88%	347459	16301	266	3465	4.49%	198076	4208	88	2046
	M. ulcerans	5805761	8.26%	364492	71682	339	4800	12.26%	540893	36626	448	5543	6.94%	306075	10994	160	3094	4.04%	178217	3088	61	1886
	M. kansasii	6402301	10.51%	463445	89051	472	6353	15.93%	702577	39990	596	7181	9.54%	420814	13458	278	4032	5.82%	256893	4132	129	2373
	M. avium	4829781	8.07%	356159	71620	322	5128	12.08%	532953	16610	194	5331	7.31%	322606	4752	110	3271	4.58%	202232	1475	65	2095
	M. haemophilum	4235765	7.08%	312375	52214	274	4137	13.05%	575862	22641	540	6284	7.98%	352034	8023	374	3703	4.94%	217744	2893	254	2322
**MTBC**	*M. caprae*	4288871	17.53%	773238	181627	598	9935	94.85%	4184378	734742	2306	37814	96.27%	4245996	725608	2253	35935	96.21%	4244109	713211	2214	34394
	M. microti	4370115	17.81%	785606	188016	825	10498	96.71%	4266542	772527	3989	40576	98.17%	4330722	771950	3873	38507	98.12%	4328596	758572	3785	36841
	M. africanum	4389314	17.87%	788161	186939	850	10494	97.15%	4285645	764554	4038	40740	98.63%	4350937	764150	3893	38685	98.60%	4349503	751103	3796	37018
	M. bovis	4345492	17.72%	781857	184148	592	10161	96.31%	4248729	750458	2304	39042	97.79%	4313964	749050	2252	36990	97.76%	4312566	735993	2213	35367
	M. tuberculosis	4411532	18.07%	797099	192022	833	10844	98.41%	4341179	791071	3947	42253	99.97%	4410355	792717	3851	40180	100.00%	4411458	779771	3777	38435

Table 2. Number of reads (per individual) used for alignment and statistical processing.

**Sample ID**	**Raw reads**	**Trimmed reads**	**Average read length**	**Non-human reads**	**Non-human reads**	**Non-human reads**	**Non-human reads**

				**(>20)**	**(>25)**	**(>30)**	**(>35)**
1_BK4	17507911	17038725	57.6	16977024	16902603	16378765	15191086
4_BK4	18816573	18215498	51.7	18095660	17960494	17086604	15246279
6_BK4	16322105	15815995	55.0	15551094	15427193	14682610	13220243
7_BK4	2231650	2160395	59.7	2102955	2095297	2047913	1936435
9_BK4	14974057	14503433	53.5	14240738	14085752	13149549	11600503
11A_BK4	16432267	16000777	58.0	15766313	15695767	15172161	14034604
11B_BK4	18522995	18078222	55.7	725913	718941	674747	597601
12_BK4	23116936	22273434	55.6	21272850	21151065	20156692	18073071
14_BK4	17849685	17383629	58.8	17310864	17235014	16752835	15595926
15_BK4	16062102	15607381	58.2	15539859	15460941	14915585	13881414
17_BK4	14980797	14496468	58.1	14426404	14372805	14078235	13247545
18_BK4	24217412	23575201	59.1	23370869	23281268	22704123	21306454
21_BK4	11890953	11500254	60.1	11271958	11237968	11021676	10439448
22_BK4	17996717	17498339	58.8	17417850	17365274	17013067	16007094
25_BK4	17560698	16997518	57.7	16888515	16816770	16375850	15237575
29_BK4	8994172	8724285	58.1	8683928	8642230	8393680	7800006
31_BK4	20427813	19941632	58.4	19741741	19684774	19309226	18187574
32_BK4	35100769	33926405	54.9	33754943	33623260	32780233	30194531
33_BK4	24501712	23719299	58.3	21669095	21595959	21031538	19569420
34_BK4	16453473	16047224	57.3	14901123	14842998	14421402	13376818
47_BK4	18736966	18155651	55.6	17998648	17903991	17174561	15478180
55_BK4	17435264	16904284	48.0	16768595	16530082	14886541	12170210
65_BK3	17465925	16921732	50.6	16735483	16587671	15466034	13185810
71_BK4	17919758	17434181	50.4	17086979	17017135	16549441	15441174
72_BK4	16355009	15952974	57.9	15874302	15812384	15444022	14541576
73_BK4	17050731	16578547	57.8	16270896	16212738	15778509	14632126
77_BK4	14044420	13478859	56.0	13390126	13322735	12866845	11763625
78_BK4	17004599	16352717	60.1	16250859	16164585	15758397	15027226

Fig 1. Changes in standardised ratio values in different read length bins and targets (red diamonds - outliers in *Mycobacterium tuberculosis* H37Rv and Borówka et al. targets in bin of reads equal or longer than 30).

Fig 2. Comparison of alignment targets constructed with different assumptions (red diamonds indicate outliers in *Mycobacterium tuberculosis* H37Rv and Borówka et al. targets in bin of reads equal or longer than 35).

Fig 1 Click here to download Figure Fig 1.docx

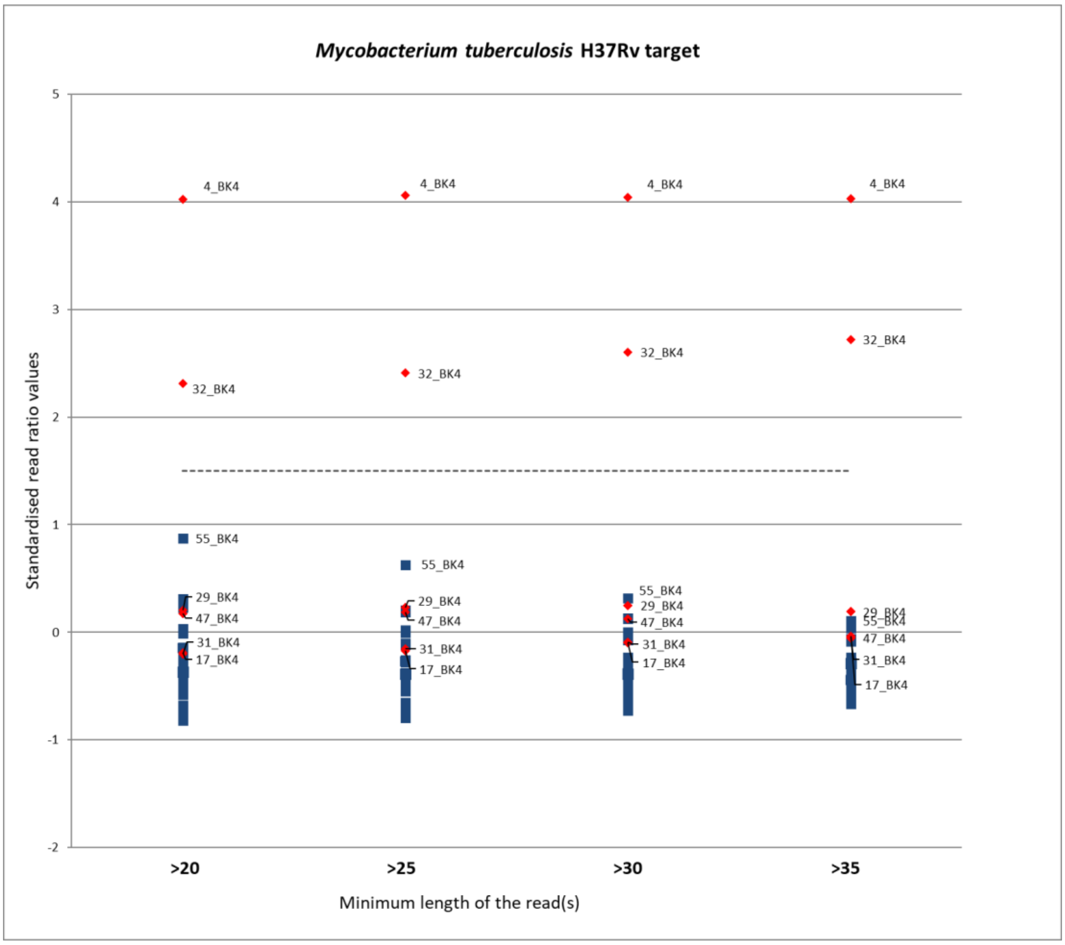

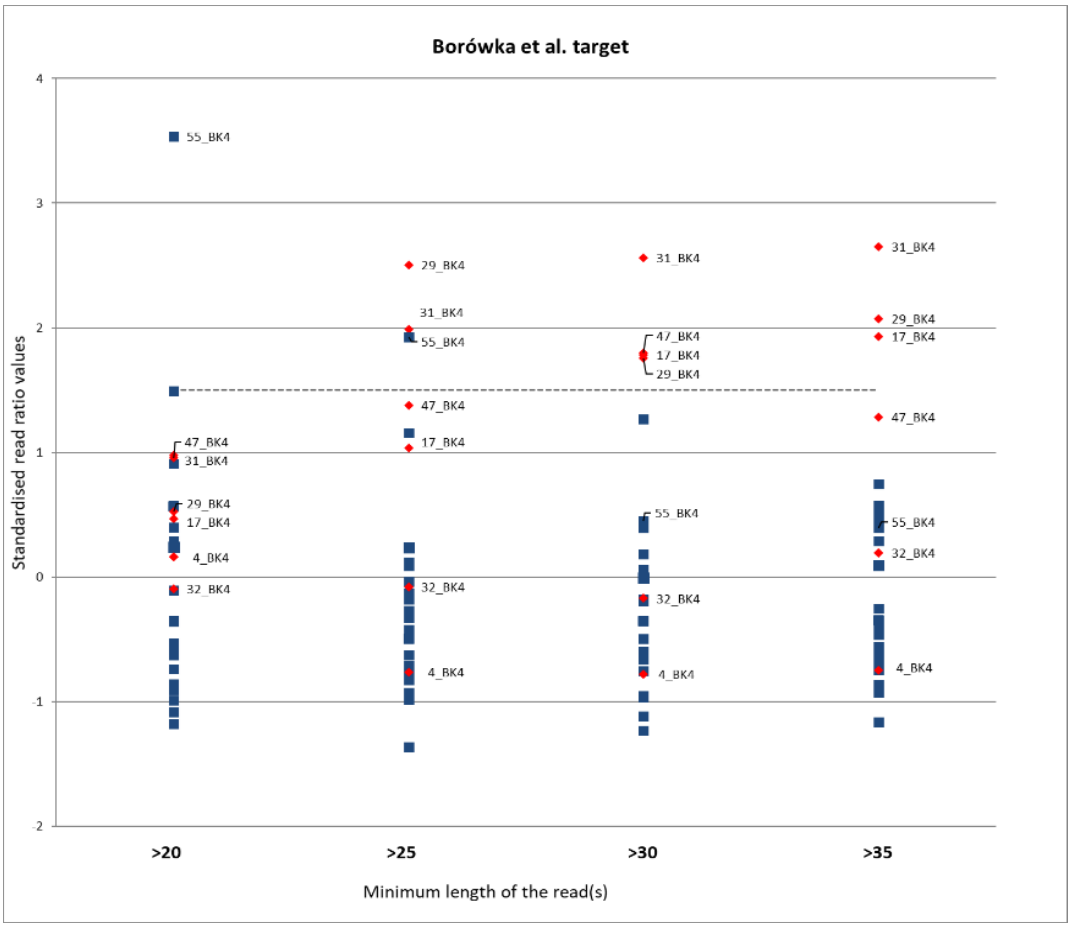

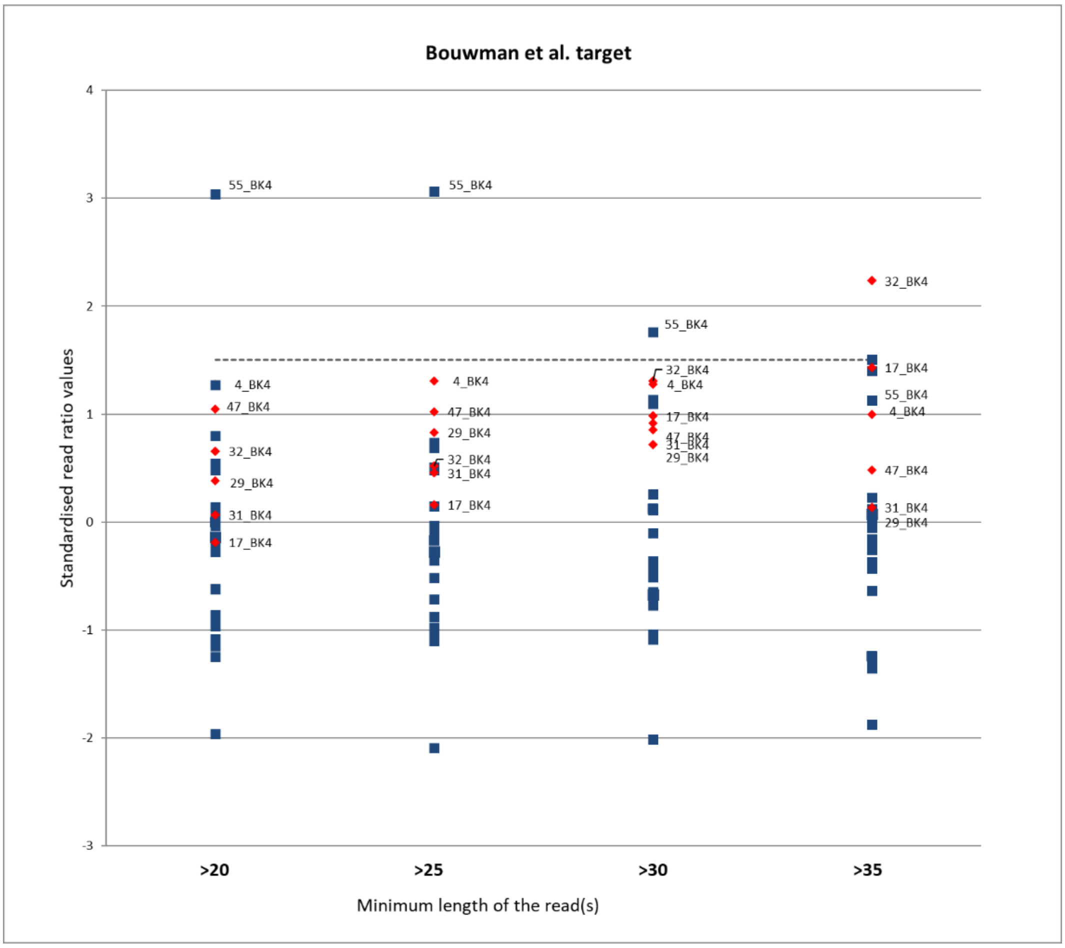

Fig 1. Changes in standardized ratio values in different read length bins and targets (red diamonds – outliers in *Mycobacterium tuberculosis* H37Rv and Borówka et al. targets in bin of reads equal or longer than 30). Dotted line present cutoff values based on 1.5×SD.

Click here to download Figure Fig 2.docx

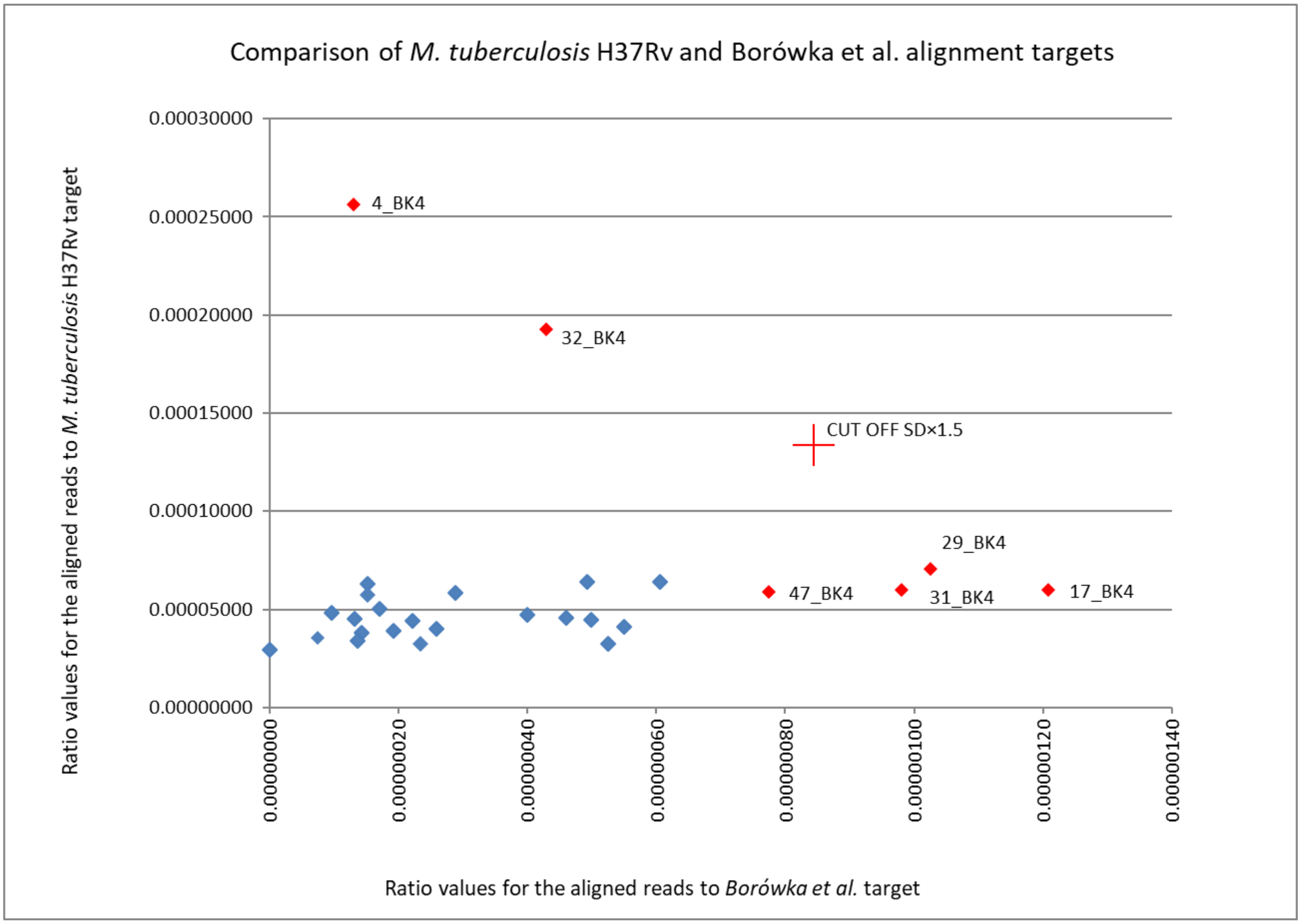

Fig 2. Comparison of alignment targets constructed with different assumptions (red diamonds indicate outliers in *Mycobacterium tuberculosis* H37Rv and Borówka et al. targets in bin of reads equal or longer than 35).

S Tables

Click here to access/download
Supplementary Material
renamed_c9c9d.xls

S Tables legend

Click here to access/download
Supplementary Material
renamed_66a18.docx

S Figures

Click here to access/download
Supplementary Material
renamed_07b58.docx

This manuscript was formerly submitted to GigaScience as GIGA-D-17-00350. It received three exhaustive reviews and as a result, the editor recommended us to “rethink the key messages” and to resubmit a substantially revised manuscript.

We thank the reviewers for the time and effort spent on suggesting ways to improve our manuscript. We used these suggestions to extensively rewrite the manuscript, perform new analyses and substantially modify both the rationale and the conclusions of the study. We hereby resubmit the manuscript and we attach a careful point-by-point explanation of modifications that were introduced in response to specific concerns of the reviewers. Therefore, we would appreciate a repetition of the peer review process and reconsideration of the possibility of publication of our study in GigaScience.

Sincerely,

Dominik Strapagiel

Reviewer 1

The Introduction (Background) gives an overview of diseases initiated in the Neolithic period and later. The cited references are not always appropriate and several do not cover points highlighted in the text.

We have now modified the Introduction to include more relevant citations and to phrase them so that the relation between our text and citations is unambiguous.

For example, the first paragraph mentions cholera, plague, leprosy, tuberculosis and malaria, yet the cited references deal only with plague (4 refs) and malaria (1). Ref 5 is a review that covers lineages and genotypes but there is nothing about pathology or lesions.

We have rephrased the introduction to remove the need to extensively cite references that would have very remote bearing on the rest of the manuscript and on the rationale of our studies, concentrating on the central justification of our research.

Ref 6 is cited with the description of the Neolithic transition yet it deals only with methods used for the MTBC. Refs 7 and 8 similarly do not mention lesions or morphology but discuss epidemiology and typing. Ref 26 deals with cattle movements rather than bovine TB. Cited ref 29 deals with venereal syphilis not TB. In paragraphs 2 & 3, ref 32 dates back to 1994 and our understanding has been updated since. Ref 33 is cited incorrectly as it describes genotypes and evolution.

These references have been replaced by more relevant ones.

The ‘often insurmountable difficulties’ in distinguishing members of the MTB complex is incorrect as this can readily be resolved on the basis of our knowledge of the specific and the shared DNA sequences.

The sentence has been rephrased to refer more specifically to knowledge gaps with regard to ancient pathogen genomes.

The controversy mentioned in line 51-52 relates to subsequent authors (e.g. refs 31 and 39) ignoring the information on the specificity of particular target sites in the repetitive sequences IS6110 and IS1081. It is clear from refs 56 and 57 that discrimination between the MTB complex and other mycobacteria is possible provided that the appropriate DNA target sequence is used. Indeed, the present authors, in their cited paper 47, state that the IS6110 site is highly discriminatory and reproducible. Paragraph 3, 2nd sentence, mentions one case when mass spectroscopy was interpreted incorrectly in one laboratory.

We have reformulated the statements referring to controversies about the sensitivity and discriminatory power of biochemical methods and IS PCR for ancient MTBC determination so that the current consensus is emphasised over past shortcomings of some published literature. We also placed more emphasis on the difficulty of specific discrimination between MTBC and MOTT.

Line 68 – suggest the paragraph heading is changed from 'Data Description' to 'Paleopathology of bone samples'.

Since the purpose of this paragraph goes beyond the paleopathological description of bone samples, including also the geographic and archeological context of the samples, we have changed the heading from "Sample description" to "Description of archeological samples".

Line 122 states that 'Fig. 1 presents the changes in standardised ratio values in different read length bins.' However, the legend in Figure 1 states that 'red diamonds – outliers in Mycobacterium tuberculosis H37Rv and Borówka et al. targets in bin of reads equal or longer than 30).' However, the figure appears to show that there are red diamonds indicating read ratios ≤ 20 and 25.

We rewrote the figure caption and the relevant paragraph in Results to explain more carefully that visual emphasis (red diamonds) is used to identify all datapoints belonging to those samples that registered as outliers in the long (relevant) read bins, even if these samples do not exceed the threshold for the length bins which include shorter reads. We chose this presentation form so that the conclusions drawn in text are more apparent at first sight, i.e. the distinction becomes apparent between samples where match ratio increases with read length and samples where match ratio stays consistently high even for shorter reads.

Line 198 – it is less confusing to the reader if the list of read numbers is the same in the text and the Figure legend.

This has now been corrected.

Lines 220-222 – again, this is an overall generalization that disregards the specificity obtained when appropriate PCR target regions are selected

Since we rewrote extensively this section of Discussion, we hope that the present phrasing does not include too sweeping generalizations on the limits of applicability of non-NGS MTBC identification methods.

In general, the Discussion is somewhat long and rambling and would have more impact if succinct.

We attempted to make the discussion more concise, while also introducing some additional aspects required by other reviewers.

All minor grammatical, spelling and style mistakes pointed out by the reviewer have been corrected in the new manuscript.

Reviewer 2

* To determine the minimum read length cut-off, the authors use 20, 25, 30, and 35 bp as thresholds and their results show that 30 bp is the optimum minimum read length for mapping. This is not a new discovery - 30 bp is a well-established minimal read length cut-off (see Schubert et al. 2012). Most aDNA studies do not use reads less than 30 bp for mapping, although some studies might use reads >= 25 bp. Hence, this finding that read length impacts specificity of mapping is not new and the authors' suggestion that reads less than 30 bp should not be used for mapping is not a significant contribution of this paper.

We agree with the reviewer that our original manuscript placed too much emphasis on the possibility of pathogen misidentification when shorter reads are used, since this risk is well-understood and mostly eliminated in literature. We have now rewritten numerous sections of the manuscript, including Introduction and Discussion, to deemphasize this element, since it is not central to the message of our study. We retain the analyses of read length bins that include shorter reads, since an important part of our reasoning is the quantitative analysis of changes that the inclusion of shorter reads cause (e.g. Supplementary Fig. 1), but we no longer claim that obtaining more specific results using reads >30 bp is novel or original to our method.

* To determine the optimum alignment target, the authors evaluate four genomic alignment targets - 1) the complete M. tuberculosis genome (strain H37Rv), 2) a 0.8 Mb-long generated alignment comprising coding sequences specific to the MTBC (designed by the authors for the purposes of this study), 3) a 0.046 Mb-long alignment (Bowman et al.), and 4) an alignment of the katG and mtp40 elements specific to the MTBC (Bos et al.). The fact that the Borówka et al. alignment is only 0.8 Mb in length means that phylogenetically important regions of the entire 4.4 Mb genome will not be covered. As such, this alignment can only be used as a first-pass approach for screening samples for the presence/absence of MTBC. However, should one desire to determine the percentage coverage of the entire genome or check for presence of lineage-specific SNPs, one would need to map the reads to the entire M. tuberculosis genome as well. As such, I am not sure whether the Borówka et al. alignment is by itself a significant contribution.

This is a very valid concern which we attempted to address already in the original manuscript, declaring that efficient phylogenetical analysis would probably require much deeper sequencing, possibly using targetted enrichment, as well as genome reconstruction; however, in the present study our goal was limited to increasing the reliability of first-pass screening with regard to identification of MTBC false positives. We consider especially important the fact that this is a relatively easy and cheap method that makes it possible to disregard unreliable osteopathological data when selecting poorly-preserved, very old samples for further, labor- and cost-intensive approaches, and we have now rewritten the manuscript to place more emphasis on this aspect of our study.

* The samples screened in this study range from 4000-6000 years old and as the authors state in Line 212, it is possible that "the preservation of MTBC aDNA was too poor to pass the sensitivity/specificity threshold of the method proposed". It is incorrect to use samples which may or may not contain MTBC DNA to test out the designed bioinformatics screening approach; furthermore, the authors do not have any positive controls. It is important to test this approach on other ancient samples which have been successful for MTBC genome recovery and reconstruction. To this end, the authors should test out this approach on publicly available data for the ancient human samples from Peru (Bos et al. 2014) and Hungarian mummies (Kay et al. 2015) to see if these are deemed as "positive outliers" by their

approach. These data are publicly available on the Sequence Read Archive and it is surprising that they were not used to test out the approach.

This very helpful suggestion led us to restructure our study, applying our analysis to the publicly available data from the Hungarian mummies study of Kay et al. (the Peruvian samples of Bos et al. were analysed exclusively by capture enrichment and thus a full NGS dataset that is required for our analysis is not available in this case). This analysis is now included in Results and Discussion and we feel it has improved the quality of our study tangibly by including a positive control and confirming the identification of “high-priority” samples for costly genome reconstruction.

*** One of the most important parameters in using BWA aln for mapping is the edit distance (defined by the -n parameter). As shown by Bos et al. 2014, using an edit distance of 0.04 (which is the default in BWA and what the authors seem to have used here), might lead to cross-mapping by closely related MOTT. Bos et al. suggest using a more stringent mapping with -n = 0.1. The authors should determine if changing this parameter affects the results of this study. To this end, I recommend re-doing the mapping with n=0.1 for all samples with the H37Rv genome and Borówka et al. alignment at least.**

This strategy was used in study Bos et al. and was appropriate for their method for library preparation (enrichment). With high number of reads that are capable for alignment this could lead to define single variation or genotyping in final consensus sequence. In our approach (without enrichment, low depth sequencing strategy) it could lead to lower number of aligned reads to reference. To obtain high specificity in our approach we have design appropriate target – Borówka et al.

* Line 2: Given the context, the "ancient host microbiome" in this sentence should be replaced by "ancient host metagenome".

* Line 7: Replace "in silico approach" by "bioinformatics approach". It is correct to say that the authors have used an in silico procedure for testing out their bioinformatics approach, as they state in Lines 171-173. However, the approach itself should be termed as a bioinformatics approach.

* In general, the Results section should be reworded to explicitly state the results of the study. The way it is written currently could lead readers to assume that the authors were able to unambiguously identify positive MTBC cases from their ancient samples, which is not the case.

We have now rephrased the Abstract to avoid the above-listed awkward expressions.

* Line 39: Given the context, the word "microbiome" should be replaced by "metagenome".

We disagree with the reviewer – in the phrase "presence of DNA and other metabolites from the whole microbiome", it is specifically the microbiome (the entire complex of microorganisms that inhabit an ecological niche, in this case the body of the deceased) that is meant and contains both DNA and metabolites, and not the metagenome (the collected genomes of these microorganisms), which contains genetic information, but certainly no metabolites.

* Lines 45-48 should be reworded. In the first sentence, the authors state that the use of MALDI-TOF to assess mycolic acids is a reliable method to identify presence of MTBC in archaeological samples. This remains a matter of contention, especially as the authors state in the next sentence that such methods have been "shown to be erroneous in some cases".

We have rephrased this sentence to be less categorical and to segue more smoothly into the subsequent one.

* In Lines 48-54, the authors talk about how PCR-based approaches are a commonly used tool for determining presence/absence of MTBC infection in the context of ancient DNA; however, the references used in this section are fairly dated. Newer aDNA studies have shown that quantitative PCR-based approaches can serve as an effective first-pass screening tool for MTBC, as a primer-probe approach is more specific and sensitive than PCR-based assays (Harkins et al. 2015). These approaches can help researchers select a subset of samples to be processed for further NGS analyses, especially since qPCR is much cheaper than high-throughput shotgun sequencing and not all researchers have the financial ability to shotgun-sequence a large number of samples at enough depth. The authors should add a sentence regarding the same to either this section or the Discussion section.

We have added a relevant sentence since it indeed makes the background presentation for our study more comprehensive.

* In Line 104, the authors suggest that the length thresholds, ranging from 17 bp to 35 bp, were chosen "to reflect the usual variability in ancient microbial sequence length used for phylogenetic analyses". aDNA researchers do not use reads less than 25-30 bp for mapping and by extension, for phylogenetic analyses. Can the authors provide any recent references which suggest that reads as small as 17 bp were used in such analyses?

We have now re-evaluated the main tenets of our study and de-emphasized the novelty inherent in mapping longer reads, since choosing them is indeed the current scientific consensus. The analysis of mapping results using reads of different length is still one of the main approaches used by us, because we found that the comparison of mapped reads (extent of changes in specificity with increasing read length) provides additional information which can be used (see e.g. Supplementary Fig. 1). We rewrote the whole manuscript to reflect this change in study rationale.

* Lines 104-105 read "Tab.1 presents the number of reads which satisfied these length criteria in DNA samples from each individual". I believe this is shown by Table 2. Hence, the tables should be interchanged.

* In Line 180, "Table 1" should be changed to "Table 2". As stated earlier, the tables should be interchanged in accordance with the order in which they appear in the Main Text.

* In Line 190, "Fig. 3" is referenced; however, there is no Figure 3 in the Main Text. I believe the authors are referencing "Supplementary Figure 3" here. The figure either needs to be moved from the Supplementary Materials to the Main Figures or the numbering of the Supplementary Figures needs to be changed accordingly.

In the new manuscript text, the order of tables and figures agrees with the order of their citation.

* In Lines 164-165, the authors state that "elimination of false positives is of paramount importance". What about false negatives? Since this is a screening approach, one can argue that false negatives are equally important, since one does not want to miss out on picking up a potentially positive sample of archaeological importance. If the workflow involves using this bioinformatics approach as a screening tool followed by target enrichment or deeper sequencing to recover whole MTBC genomes, then one need not worry about a potential false positive sample being carried through to the second stage, since these will likely drop out during the target enrichment process or further bioinformatic analyses of target-enriched data.

We now rewrote both this paragraph of Results and the Discussion to more precisely reflect our thesis that our screening approach with its inherent high specificity due to increased attention to possible MOTT contaminants is especially useful in studies with highly degraded samples (necessitating enrichment and/or high coverage sequencing) and limited means, where “dropping out” of false positive

samples at the costly deep sequencing stage would mean an unacceptable and unsustainable financial burden on the study.

* In Supplementary Figure 2, are the damage plots based on reads mapping to the entire M. tuberculosis genome? This needs to be explicitly stated. It would be interesting to see MapDamage plots for both mapping schemes (H37Rv and Borówka et al.) for all five samples.

We have done mapDamage analysis for selected six individuals (possibly MTBC positive or MOTT positive), for confirmation of ancient status of analyzed samples. In current version of manuscript deamination patterns are shown in Supplementary Fig. 3.

* In Lines 213-214, the authors state that "for diagnosis of ancient tuberculosis cases, biochemical methods based on mass spectrometry are very promising". I would reword this to say that these methods are very promising and useful in a contemporary epidemiological context. However, as the authors state in Lines 216-219, there are several hindrances to using these methods in the context of ancient DNA.

* In general, the Lines 213-223 seem repetitive since the content has already been mentioned in the Background section.

* In Lines 223-225, the authors mention how NGS has been used to diagnose cases of ancient tuberculosis. The Kay et al. 2015 paper in which a metagenomics approach was used to recover near-complete MTBC genomes from 18th century Hungarian mummies needs to be mentioned here, as it is the only study till date which has allowed for ancient MTBC genome reconstruction without the use of target enrichment techniques.

* In the paragraph ending on Line 227, I would recommend the authors also discuss the use of target enrichment methods to capture whole MTBC genomes from ancient DNA samples since target enrichment is now a widespread tool in the field of ancient pathogen genomics.

We now entirely rewrote the Discussion section to include all the above suggestions and remarks, and to reflect the changed overall rationale of the study.

* In Lines 246-248, the authors state that "If a highly specific method like the one we propose is used to identify likely ancient MTBC infection, potential lineage determination should proceed by other methods developed specifically for this purpose, e.g. presence of TbD1." Given the small size of the Borówka et al.

alignment, MTBC lineage-specific SNPs (Coll et al. 2014, Stucki et al. 2016) might not be covered. The TbD1 deletion can only be used to classify a strain into the "modern" and "ancient" lineages and does not give further information about the phylogenetic relationships. Currently, in both modern as well as ancient contexts, lineage-determining SNPs are an important and well-established method of classification, which the authors should note. Using only their alignment target might lead to the presence/absence of these SNPs not being determined in the ancient samples screened and the authors should not that this is a limitation of their approach.

Obviously, for a full phylogenetic study, a dataset which is both as broad and as deep as possible is a prerequisite. While preliminary lineage assignment can proceed on the basis of fragmentary data (e.g. TbD1 presence, individual lineage-specific SNPs etc.), this approach is inherently limited – instead, modern (albeit costly) genome reconstruction techniques allow a reliable full genome-based phylogenetic analysis. Our study should be meant as a cost-effective pre-screening algorithm for poorly preserved ancient samples, to be selected for the full deep sequencing pipeline. We have rewritten the Discussion to unequivocally reflect this approach.

* For Lines 252-253, is there a reference that can be cited for this statement?

Even though the original sentence is rephrased in this version of Discussion, we have now included a reference that deals with contamination with soil MOTT.

* In the context of Lines 261-263, the authors identify M. marinum as a potential infectious agent present in two samples in this data-set. M. marinum infection typically occurs opportunistically in individuals working with fish, such as in aquariums. Given the archaeological context of these individuals, can the authors expand on whether it is possible that these individuals were infected by M. marinum and what could potentially be a source of this infection?

We have now added a sentence that deals specifically with this topic.

* After reading the Ancient DNA Extractions section, it seems unclear to me how the authors can conclude that ancient DNA was successfully recovered from the samples. An easy way to assess this would be to map the shotgun data to the human genome (either whole or mitochondrial) and assess host endogenous DNA preservation and damage patterns. The authors should include MapDamage plots showing that the human DNA present in these samples shows patterns characteristic of aDNA.

* Line 276 states that "Ancient DNA was successfully isolated from all bone samples." The authors need to elaborate on how this was determined.

This was determined by performing MapDamage analysis on the human sequences in isolated DNA – this information is now included in the manuscript as a Supplementary Fig. 3.

* In lines 276-277, the authors state that "Illumina libraries were prepared in a separate facility according to Meyer et al." The authors do not specify that these samples were treated with UDG enzyme before library preparation. It would be helpful to explicitly state this. Since the presence of post-mortem damage was assessed using MapDamage, I am assuming that these are non-UDG treated libraries. When mapping the data from non-UDG treated libraries, certain BWA mapping parameters should be changed, such as disabling the seed (Schubert et al. 2012).

A more detailed description of this part of analysis protocol, clarifying the above-described ambiguities, is now included.

* In Lines 284-285, the authors do not state whether the FASTQ reads were quality filtered before mapping. What is the quality threshold of the reads that were used as input for mapping? Most aDNA studies use a cut-off of Q20 at this stage (Schubert et al. 2012).

* In Lines 286-287, the authors need to expand on how the mapping to the human genome was conducted, especially since many readers might be unfamiliar with the AGAT mapping tool. What parameters were used?

* For the BWA alignments to the four MTBC targets, was any post-mapping quality filtering conducted? For example, most aDNA studies would filter out reads at a threshold of at least Q30 (or even Q37). Was any duplicate removal conducted? It might be that too few reads mapped to the target to conduct quality filtering and/or duplicate removal, but in that case, the authors need to state this explicitly.

A more detailed description of this part of analysis protocol, clarifying the above-described ambiguities, are now included in methods section.

Reviewer 3

First, as authentication is the focus of this manuscript, I refer the authors to two relevant review papers that discuss this: Warriner et al (Annual Review of Genomics and Human Genetics, 2017) and Key et al. (Trends in Genetics, 2017). At least one of these works should be cited, and application of the authentication criteria therein to the current dataset would be valuable.

This has now been corrected. The Kay et al dataset was used for verification of proposed Borowka et al approach.

The criterion upon which they place the greatest focus is a bit surprising. The authors have chosen to direct their efforts toward read length filtering of metagenomic data. They perform several tests to assess potential false positives from several read length categories from 17 to >35bp, as determined by mapping statistics against three sets of TB complex references, one from Bouwman et al, a reduced set from Bos et al., and a new set established here. Ultimately, they conclude that read lengths of 30bp or greater are required for confident mapping. Such a result is not a surprise. To my knowledge, applying a length filter of 30 to read data before mapping is standard in ancient DNA work with NGS data. In this sense, I am not convinced of the relevance of the paper in its current form. I suggest they peruse the literature closely to determine how common the length filtering is. If I am incorrect, and length filtering is not common, then the authors should include a short justification for the importance of this filter and the necessity to investigate it. If length filtering is indeed common practice, I suggest the authors shift their focus to the selection of reference sequences for reliable mapping of pathogenic mycobacteria. This aspect of the paper is novel and the results are highly useful for screening metagenomic datasets, if sequenced to sufficient depth.

We agree with the reviewer that our original manuscript focused mostly on the estimation of the proper read length used for alignment in ancient DNA studies. In current version of manuscript this issue was rewritten, with emphasis that obtaining more specific results using reads >30 bp is not original to our method. Purpose of using reads of different length with comparison of obtained results could be informative on some point, and in our opinion still provides additional information about analyzed samples.

I found the order of the analyses counterintuitive. They first present authenticity tests on ancient metagenomic data for which they have no information on TB DNA survival. At the end of the results section, they describe an in-silico test using artificially fragmented modern genomes to test the reliability of their screening approach. This should be presented the other way around. It would strengthen the manuscript to first perform a series of tests using simulated short reads from modern MTBC and environmentally-derived genomes to establish their most reliable reference template, mapping parameters, and length filtering, and then use these parameters on an ancient DNA set as a

provisional screening. Also, I suggest the authors use Gargammel (Renaud et al. Bioinformatics, 2017) to give their modern reads a simulated ancient DNA damage profile.

Manuscript was rewritten due to comments and strengthen on developed approach. Current version of manuscript obtains additionally mapping results from Kay et al. dataset, with confirm usage of Borówka et al. approach in screening detection of *Mycobacterium tuberculosis* complex members in ancient samples.

I find the authors overconfident in their identifications of MTBC reads in the ancient data. As there are too few reads to perform mapdamage analysis, the authors should be clear that they have identified suggestive cases that await confirmation via other means. As the mapped reads extremely are few, it might be advantageous to assess their positions visually in an alignment viewer to see if they are clustered in short regions, or if they have SNPs that differ from the reference. With low coverage positive data, the expectation is that reads will be uniformly distributed across the reference template for non-repeated sections and have little sequence divergence. This would make a suitable figure for their supplementary, perhaps for the five samples that have the highest mapping reads for the >30 category (especially since two are suspected MOTT cases). Their repeated use of the term "positive" reads for those greater than 30bp mapping to a TB reference is presumptuous, and implies they are in fact derived from an ancient pathogen, which can't be determined at this stage.

The statement in their abstract that their methods "provide statistically supported identification of ancient disease cases" is too strong. This is again echoed in the authors' assertion that all samples with read ratio values of greater than 1.5SD are "confirmed cases of TB infection" (line 134). This is especially important when considering Figure S3, which reveals all samples to have a decent number of reads mapping to M. marinum. In this figure, the three samples regarded as the best candidates for ancient TB infections should be shown in addition to the two outliers that are suspected MOTT cases.

"The statement in their abstract that their methods "provide statistically supported identification of ancient disease cases" is too strong" - the sentence has been softener in the current version of manuscript. Supplementary Fig. 2 have marked candidates for ancient TB infection.

In short, I suggest the authors redirect their focus to in-silico based tests, and present the ancient data set as a test case that awaits downstream authentication.

Manuscript has been rewritten and these issues are now corrected.

Minor points:

Descriptions of non-NGS detection methods should be limited to the introduction and not revisited in the discussion unless they apply to the specific dataset presented in the manuscript.

This has now been corrected.

The authors should state in the results section the sequencing platform and the sequencing depth. While these are technically methods, knowing this is essential to properly interpreting their mapping results.

This information has been added in Analyses section.

Line 50 – remove the word "proven", especially since the next sentence explores problems with PCR-based approaches

This has now been corrected.

Line 92 – This sentence is confusing. I suggest the authors state "specific to the complex" defined as human lineages 1 – 7 and the animal lineages.

This has now been corrected.

Lines 106 – 114 – The authors should mention that reads mapping to hg19 were removed

This has now been corrected.

The caption for supplementary figure 3 needs greater explanation

This has now been corrected.

It would be helpful to present a table disclosing the results from the M. marinum mapping.

In current version of manuscript, we append supplementary table 10, with alignment results for read length >30 for this target.
