# Supplementary material for: Screening methods for detection of ancient Mycobacterium tuberculosis complex fingerprints in next-generation sequencing data derived from skeletal samples
Source: Gigascience. 2019 Jun 20;8(6):giz065. doi: 10.1093/gigascience/giz065 (PMC6586198; doi:10.1093/gigascience/giz065)
Supplement: giz065_Supplemental_Files [file giz065_supplemental_files.zip › renamed_42438.docx]

**Supplementary Table 1.**

Differential diagnosis of tuberculosis-like lesions of the spine in paleopathology (after [5, 6]).

**Supplementary Table 2.**

Summarized information about 28 individuals (BK4 and BK3 – Brześć Kujawski site 4 and 3; F- female, M – male), with detailed information about bone samples derived for aDNA analysis in column 5. Column 6 describe presence of observed lesions in the analyzed skeletons.

**Supplementary Table 3.**

Summarized information about alignment results for Kay et al. (2015) *M. tuberculosis* positive Individuals, used as a verification of Borówka et al. bioinformatical approach in comparision to the other used targets.

**Supplementary Table 4.**

Summarized information about number of reads within each read length bin that align to the Borówka et al. genomic target. AVG - arithmetic mean; STDEV - standard deviation; CUTOFF 1.5 - AVG×(1.5×STDEV).

**Supplementary Table 5.**

Summarized information about number of reads within each read length bin that align to the *Mycobacterium tuberculosis* H37Rv genomic target. AVG - arithmetic mean; STDEV - standard deviation; CUTOFF 1.5 - AVG×(1.5×STDEV).

**Supplementary Table 6.**

Summarized information about number of reads within each read length bin that align to the Bouwman et al. (2012) genomic target. AVG - arithmetic mean; STDEV - standard deviation; CUTOFF 1.5 - AVG×(1.5×STDEV).

**Supplementary Table 7.**

Summarized information about number of reads within each read length bin that align to the Bos et al. (2015) genomic target. AVG - arithmetic mean; STDEV - standard deviation; CUTOFF 1.5 - AVG×(1.5×STDEV).

**Supplementary Table 8.**

Arithmetic mean for calculated ratios (read ratios as percentage of average positive read ratio) for adjoining read length bins (≥25bp/≥20bp, ≥30bp/≥25bp and ≥35bp/≥30bp) for each individual.

**Supplementary Table 9.**

Summarized information about number of reads aligned to the *Mycobacterium tuberculosis* H37Rv and *Mycobacterium marinum* (NC010612.1) genomic target.

**Supplementary Table 10.**

Borówka et al. 1534 query sequences presented and used in this study for detection of *Mycobacterium tuberculosis* complex infected individuals.
