# Supplementary material for: Screening methods for detection of ancient Mycobacterium tuberculosis complex fingerprints in next-generation sequencing data derived from skeletal samples
Source: Gigascience. 2019 Jun 20;8(6):giz065. doi: 10.1093/gigascience/giz065 (PMC6586198; doi:10.1093/gigascience/giz065)
Supplement: giz065_Supplemental_Files [file giz065_supplemental_files.zip › renamed_c76da.docx]

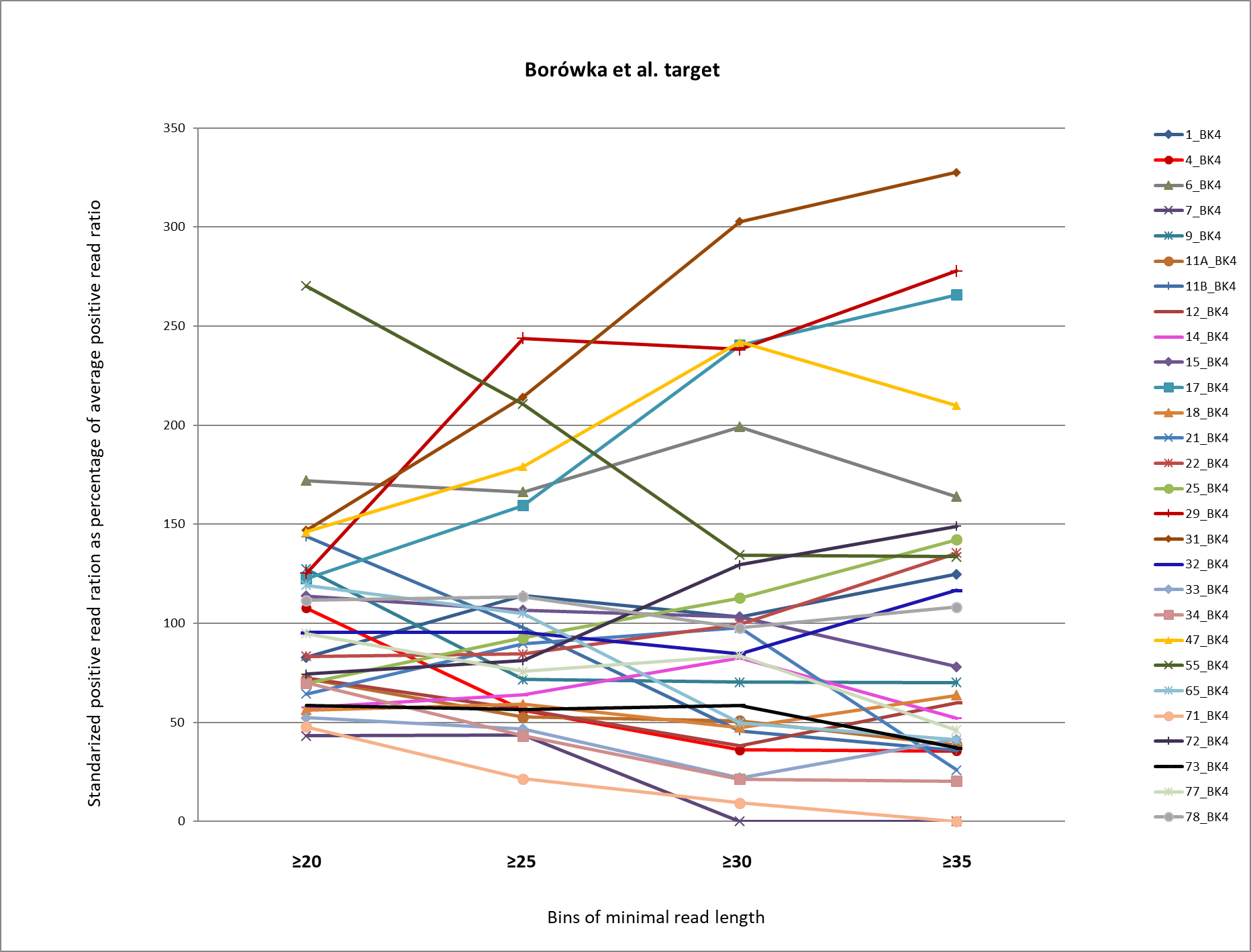


Supplementary Fig. 1 Standardized positive read ratios as percentage of average positive read ratio for Borówka et al. target.


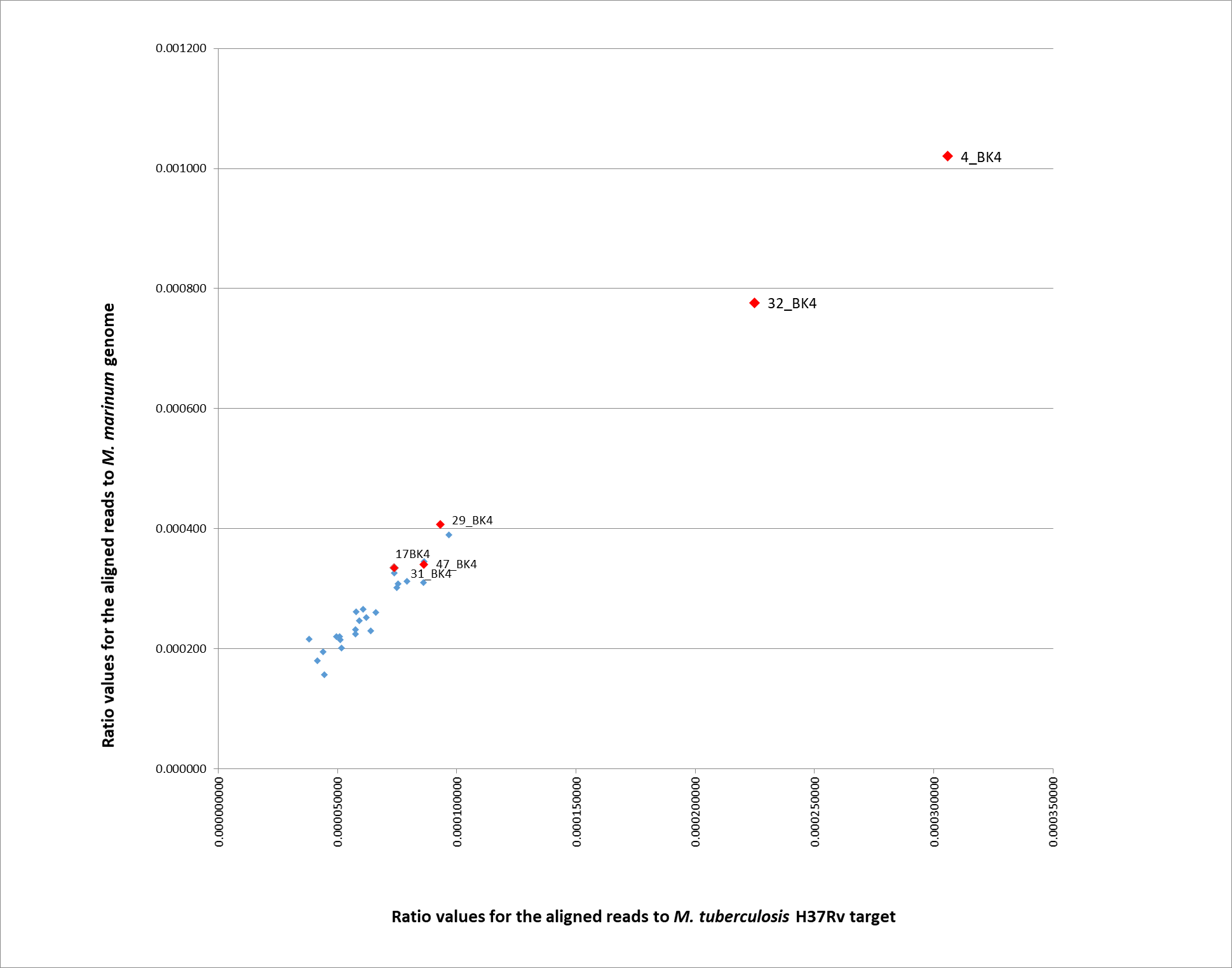


Supplementary Fig. 2. Comparison of alignment targets *M. tuberculosis* H37Rv and *M. marinum genomes* (red diamonds indicate outliers in *Mycobacterium tuberculosis* H37Rv and *M. marinum* all targets in bin of reads equal or longer than 30).

**a)**


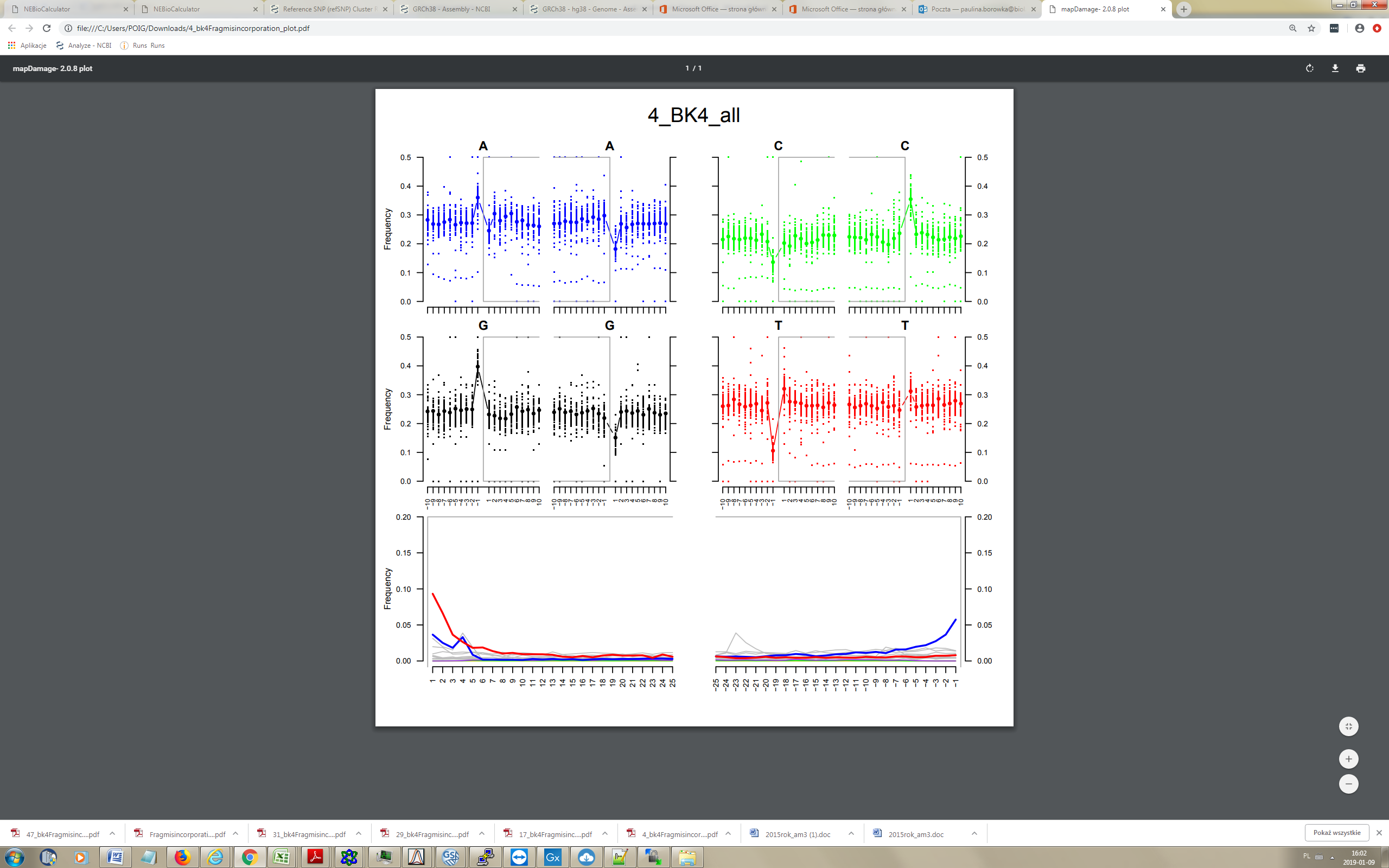


**b)**

**
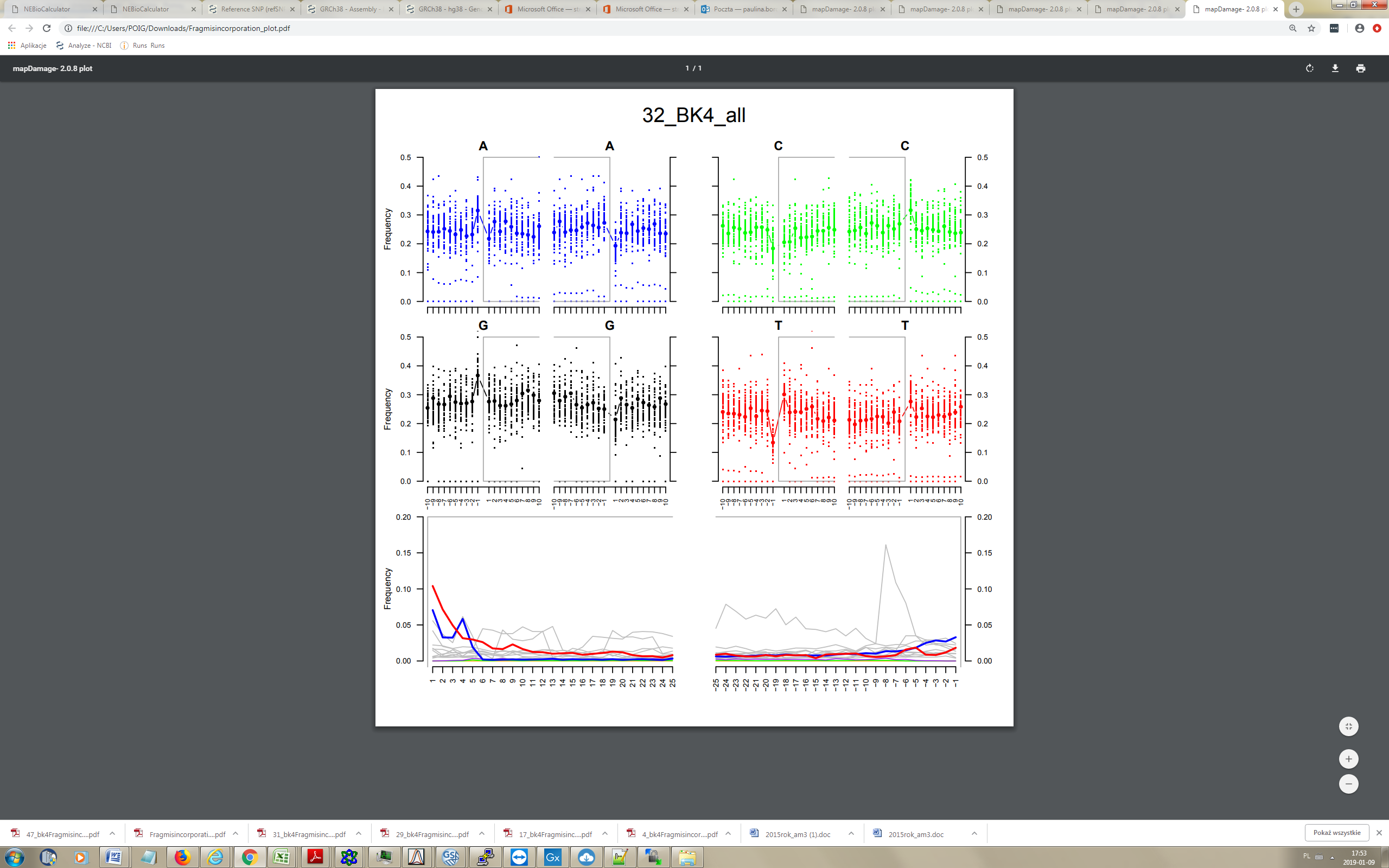
**

**c)**


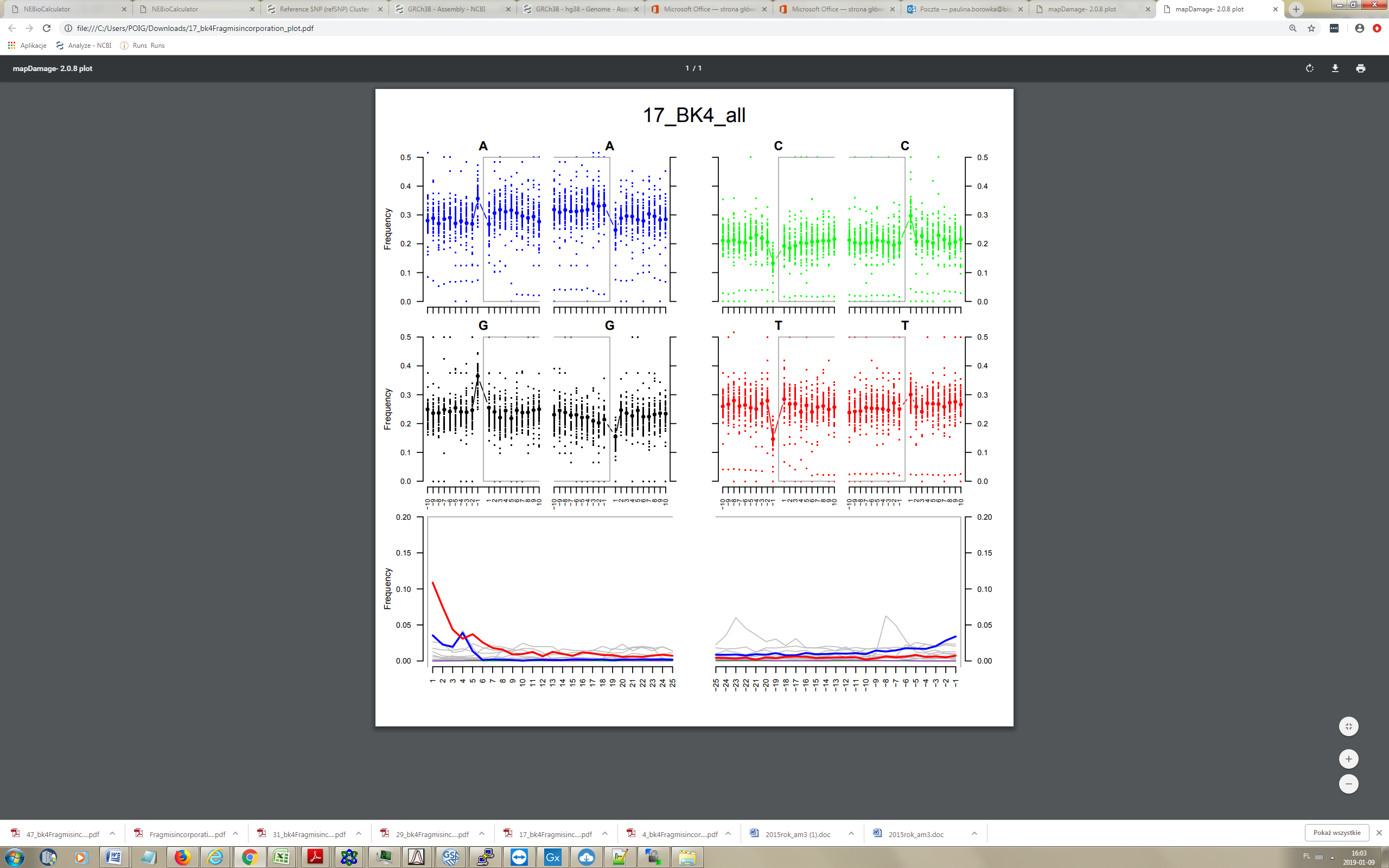


**d)**


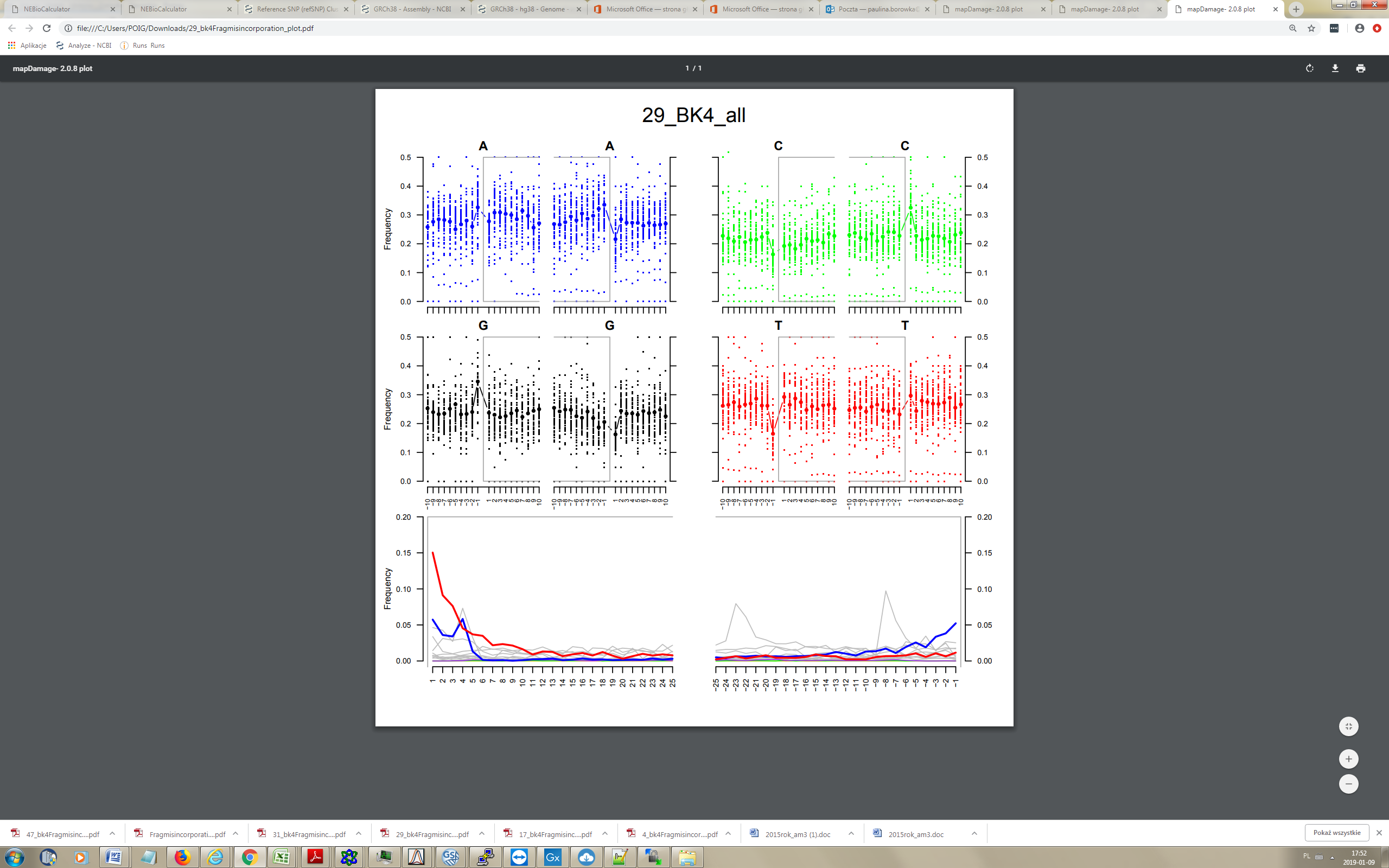


**e)**


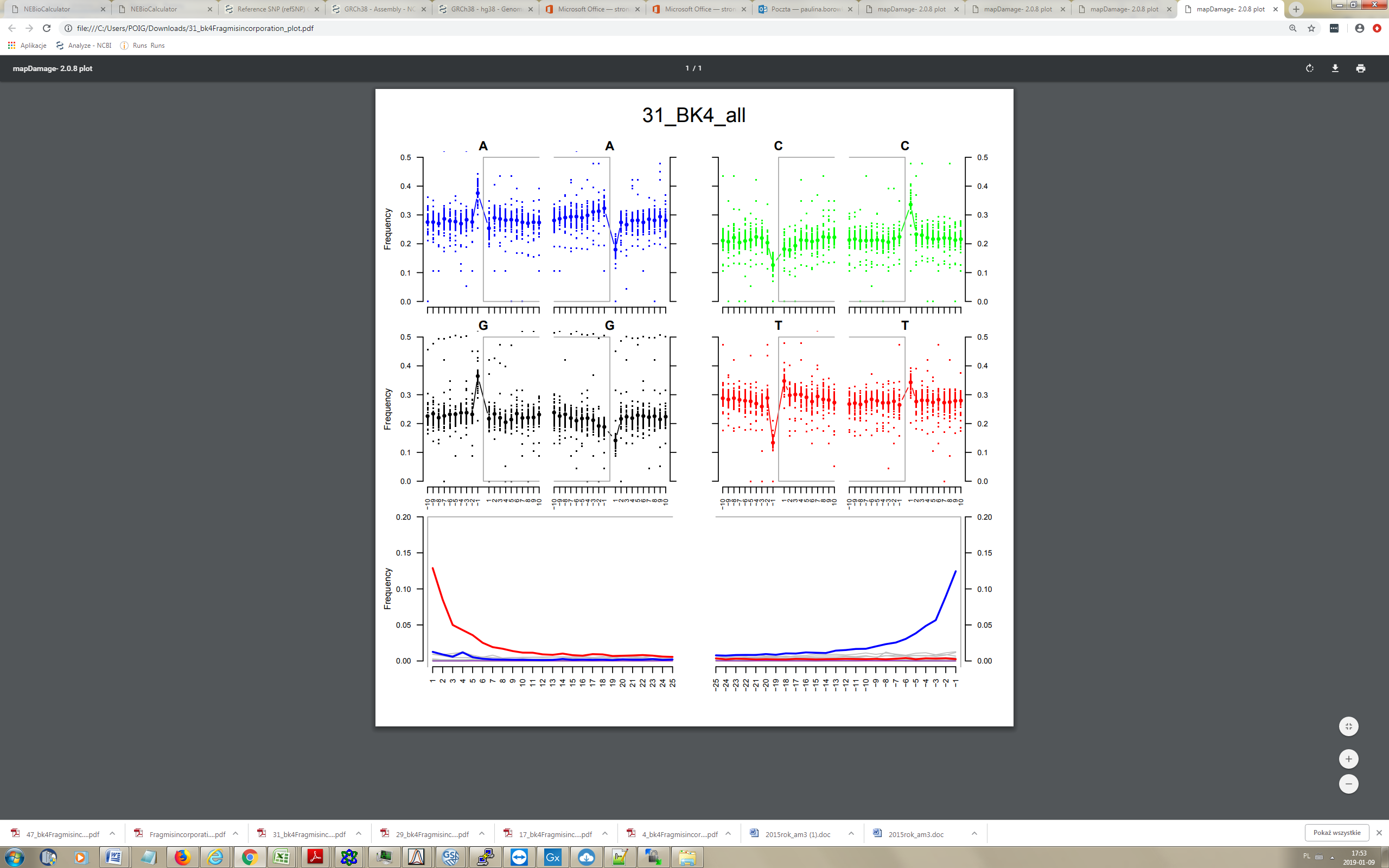


**f)**


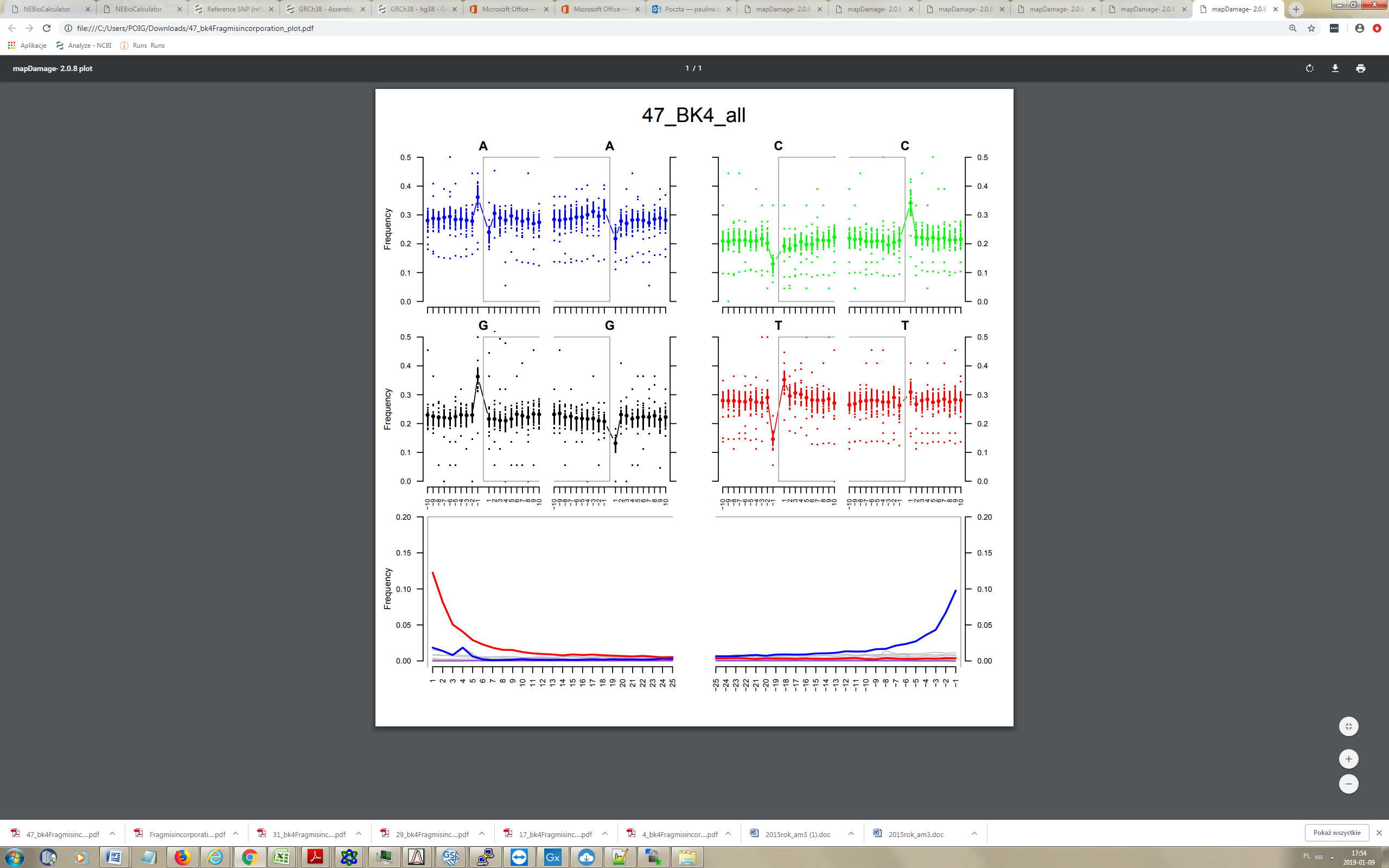


Supplementary Fig. 3. MapDamage patterns for samples indicated as a possible MOTT infection (^a^4_BK4, ^b^32_BK4), and potential *M. tuberculosis* sufferers (^c^17_BK4, ^d^29_BK4, ^e^31_BK4, with additional sample ^f^47_BK4) , based on human reads (aligning to human genome build 37) with length ≥30. Due to low number of non-human reads attributed to each sample after mapping step, it was not useful to analyse MapDamage patterns on reads aligning to *M. tuberculosis* genome. Blue color indicates G to A transitions, red color indicates C to T transitions per sample.

a)


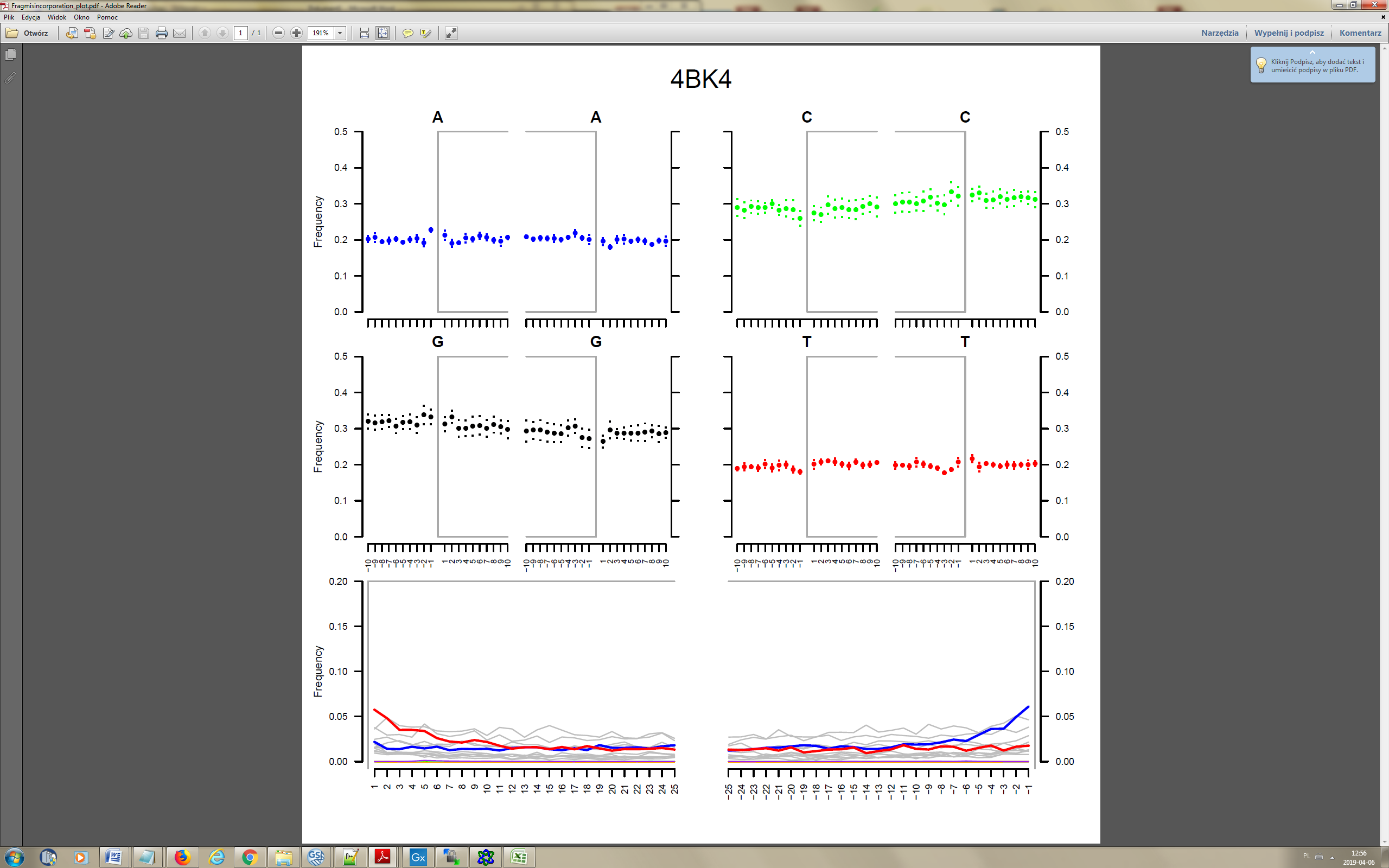


b)


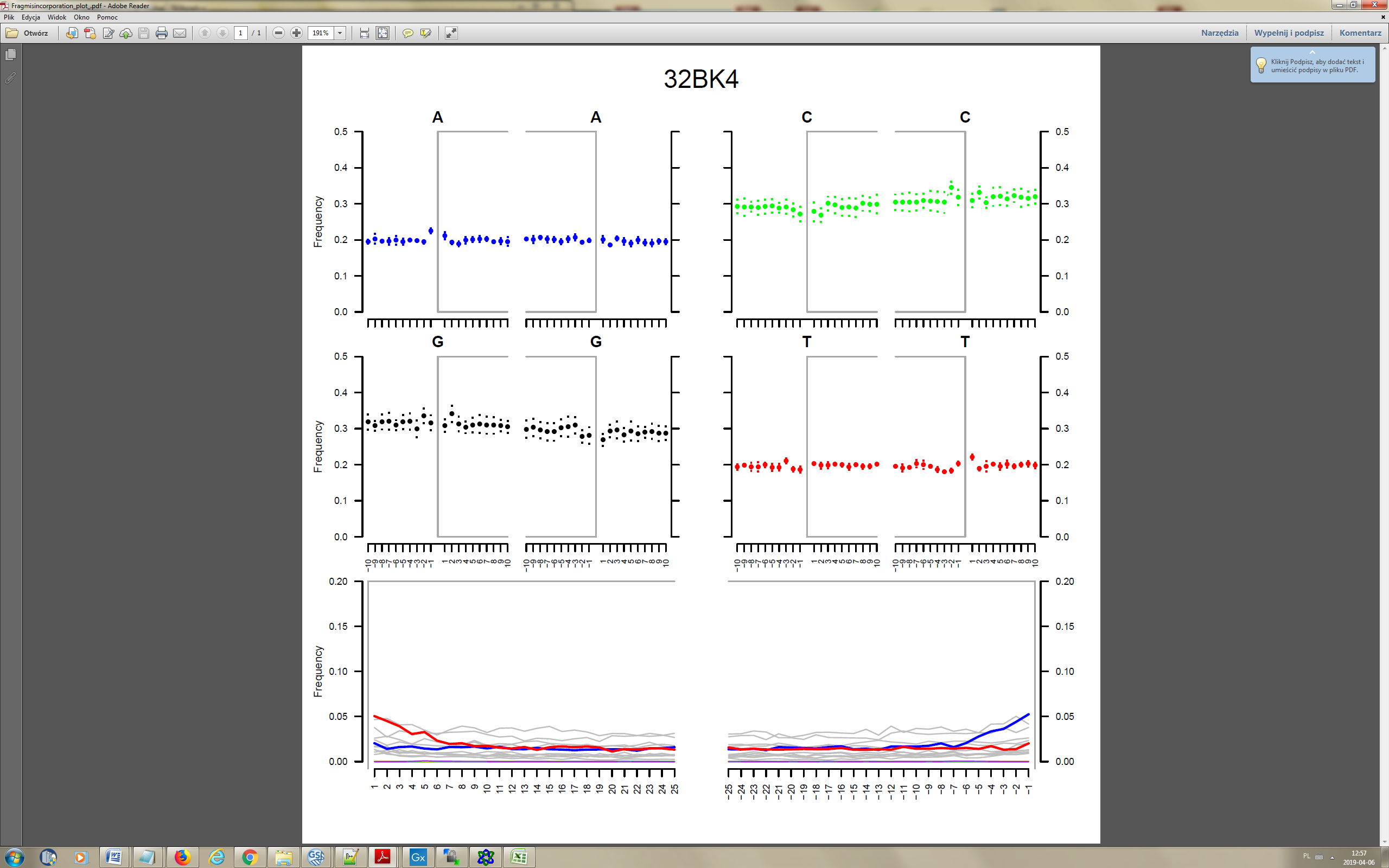


Supplementary Fig. 4. MapDamage patterns for samples indicated as a possible MOTT infection (^a^4_BK4, ^b^32_BK4), based on reads with length ≥30 aligning to the *M. marinum* genome. Blue color indicates G to A transitions, red color indicates C to T transitions per sample.
