# Supplementary material for: Screening methods for detection of ancient Mycobacterium tuberculosis complex fingerprints in next-generation sequencing data derived from skeletal samples
Source: Gigascience. 2019 Jun 20;8(6):giz065. doi: 10.1093/gigascience/giz065 (PMC6586198; doi:10.1093/gigascience/giz065)
Supplement: giz065_Response_to_Reviewer_Comments_Original_Submission [file giz065_response_to_reviewer_comments_original_submission.pdf]

Author's Response To Reviewer Comments

Close

Lodz, 7th of April, 2019
Dear GigaScience Editor,
We appreciate the insightful and detailed comments of the reviewers. We included all the minor language-related corrections in the resubmitted manuscript. Following is a point-by-point reply to the major points raised by the reviewers.
Reviewer 1
1. Our statistical approach, as we state directly in the manuscript, is aimed at finding positive outliers in a pool of samples with unknown presence of mycobacterial sequences. When applied to a dataset like Kay et al., which consists exclusively of individuals with previously confirmed ancient mycobacterial infection, its outcome is therefore necessarily limited to identifying the outliers with highest microbial load (in the case of tuberculosis, potentially those individuals who died during the active phase of the disease) - these outliers being by definition always a minority of analysed samples. This explanation is provided in the text of the manuscript.
2. We have performed the MapDamage analysis suggested by the reviewer and it indeed yielded a positive result - we thank the reviewer for this suggestion as this strengthened our conclusions significantly. We have now included a new Supplementary Fig. 4, and we have reworded both the legend to Supplementary Fig. 3 and the sentences in the manuscript that refer to it.
3. Libraries were build using Meyer et al. (2010) protocol with modifications proposed by Gamba et al. (2014). We have performed mapDamage analysis in the way which fit to double stranded libraries. Information about different Meyer protocol and single stranded libraries was incorrectly added to previous version of the manuscript.
4. We have expanded Supplementary Tab. 2 to include the absolute numbers of reads - both total and aligning to each alignment target.
Reviewer 2
1. We have now expanded the analysis of state of the art in biochemical detection of ancient mycobacteria by citing recent articles mentioning improvements in cell wall component analysis.
2. Indeed, Pott's disease is usually regarded as pathognomonic signature of TB. When we said that many pathological conditions of the spine can mimic Pott's disease thought that they can be diagnosed mistakenly as tuberculosis, especially in practice with poorly preserved skeletons. The present text has been appropriately modified (both in the Introduction and Discussion) to clarify this statement. Moreover, according to the Reviewer's suggestion, the list of pathological conditions has been replaced with the Table S1, which include a short description of basic differences between these lesions and bone tuberculosis.

Dear Editor, regarding to your comment:
“As your revised manuscript focuses more on a method, it may be more suitable as a "Technical Note" rather than a "research article" “
We would like to proceed this manuscript as research paper. In our work we present original dataset which allow us to present a novel bioinformatical approach, used for screening of ancient tuberculosis in sequencing data, derived from 28 individuals (dated 4400 - 4000 BC and 3100 - 2900 BC) from Central Poland. That dataset was not previously published elsewhere.

Sincerely,
Dominik Strapagiel

Close
