# Supplementary material for: Screening methods for detection of ancient Mycobacterium tuberculosis complex fingerprints in next-generation sequencing data derived from skeletal samples
Source: Gigascience. 2019 Jun 20;8(6):giz065. doi: 10.1093/gigascience/giz065 (PMC6586198; doi:10.1093/gigascience/giz065)
Supplement: giz065_Reviewer_1_Report_Original_Submission — Tanvi Honap -- 2/27/2019 Reviewed [file giz065_reviewer_1_report_original_submission.pdf]

Reviewer Report

Title: Screening methods for detection of ancient Mycobacterium tuberculosis complex fingerprints in NGS data derived from skeletal samples

Version: Original Submission **Date:** 2/27/2019

Reviewer name: Tanvi Honap

Reviewer Comments to Author:

In this manuscript, Borowka et al. present a strategy for analyzing ancient shotgun metagenomic data for presence of Mycobacterium tuberculosis complex (MTBC) DNA, to the exclusion of other environmental and/or pathogenic mycobacterial DNA which can confound the analysis. Their approach will be of interest to ancient DNA researchers working in the field of pathogen genomics, especially tuberculosis.

The authors have sufficiently addressed my concerns/comments on the earlier version of this manuscript. I do have a couple of questions, which should be addressed before the manuscript is accepted -

1. Given that nearly all the samples from Kay et al. yielded M. tuberculosis genomes, why does the authors' statistical approach when comparing the Kay et al. data to the H37Rv genome only show three samples as positive outliers? I understand that the four samples shown to be outliers with the Borowka et al. alignment are the ones with high microbial load, but when comparing to the entire H37Rv genome, shouldn't all (or at least most of) the samples be considered as positive outliers?
2. In Supplementary Figure 3, the authors show MapDamage plots for six samples - two of which are potentially positive for M. marinum and three others for MTBC. Yet, the MapDamage plots are based on mapping to human reads. Looking at Supplementary Tables 4 and 8, the two M. marinum samples (4_BK4 and 32_BK4) have approx. 17,000 and 25,000 reads mapping to the M. marinum genome. The MTBC samples have approx. 700 - 1,400 reads mapping to the H37Rv genome. The authors should at last be able to generate MapDamage profiles for the M. marinum samples. >1000 reads mapping to the M. tuberculosis or M. marinum genome should be sufficient to generate a damage plot. Without the MapDamage plots, it is difficult to ascertain whether the samples actually contain ancient mycobacterial DNA or not. Also, Lines 220-222 need to be reworded to specify that the MapDamage analysis was performed on reads mapping to the human genome.
3. Line 332 - If I am understanding this correctly, these libraries were not built using the Gansauge and Meyer (2013) single-stranded library build protocol. Hence, MapDamage should not be run using the --single-stranded parameter.
4. Lines 346 - 347 - Statistics of read mapping of the Kay et al. data to the four genomic alignment targets are not given in Supplementary Table 2. The table only shows which samples were considered as positive outliers by each method. Adding a column with the absolute number of reads >30 bp mapping to each target would be helpful. If this cannot be added, then the line should be modified so as to avoid confusion for the reader.
5. Minor changes -

Line 18 - Modify "Neolithic period" to "the Neolithic period"
Line 63 - Change "Main purpose" to "The main purpose".
Line 64 - A better segue is needed between this and the previous sentence.
Line 103 - Remove the word "since"
Line 140 - Define aTB as ancient tuberculosis the first time it is used.
Line 304 - Remove the word "e.g" or reword the sentence

Methods

Are the methods appropriate to the aims of the study, are they well described, and are necessary controls included? Choose an item.

Conclusions

Are the conclusions adequately supported by the data shown? Choose an item.

Reporting Standards

Does the manuscript adhere to the journal's guidelines on minimum standards of reporting? Choose an item.

Choose an item.

Statistics

Are you able to assess all statistics in the manuscript, including the appropriateness of statistical tests used? Choose an item.

Quality of Written English

Please indicate the quality of language in the manuscript: Choose an item.

Declaration of Competing Interests

Please complete a declaration of competing interests, considering the following questions:

- Have you in the past five years received reimbursements, fees, funding, or salary from an organisation that may in any way gain or lose financially from the publication of this manuscript, either now or in the future?
- Do you hold any stocks or shares in an organisation that may in any way gain or lose financially from the publication of this manuscript, either now or in the future?
- Do you hold or are you currently applying for any patents relating to the content of the manuscript?
- Have you received reimbursements, fees, funding, or salary from an organization that holds or has applied for patents relating to the content of the manuscript?

- Do you have any other financial competing interests?
- Do you have any non-financial competing interests in relation to this paper?

If you can answer no to all of the above, write 'I declare that I have no competing interests' below. If your reply is yes to any, please give details below.

I declare that I have no competing interests.

I agree to the open peer review policy of the journal. I understand that my name will be included on my report to the authors and, if the manuscript is accepted for publication, my named report including any attachments I upload will be posted on the website along with the authors' responses. I agree for my report to be made available under an Open Access Creative Commons CC-BY license (http://creativecommons.org/licenses/by/4.0/). I understand that any comments which I do not wish to be included in my named report can be included as confidential comments to the editors, which will not be published.

I agree to the open peer review policy of the journal

To further support our reviewers, we have joined with Publons, where you can gain additional credit to further highlight your hard work (see: https://publons.com/journal/530/gigascience). On publication of this paper, your review will be automatically added to Publons, you can then choose whether or not to claim your Publons credit. I understand this statement.

Yes Choose an item.
